# Supplementary material for: Prevalence and determinants of insulin resistance in recovered COVID-19 and uninfected residents of two regional capitals in Ghana: An observational study
Source: PLOS Glob Public Health. 2025 Apr 24;5(4):e0004506. doi: 10.1371/journal.pgph.0004506 (PMC12021235; doi:10.1371/journal.pgph.0004506)
Supplement: Attachment — Submitted filename: PGPH-D-24-00529_R3.pdf [file pgph.0004506.s006.pdf]

# PLOS Global Public Health


## PREVALENCE AND DETERMINANTS OF INSULIN RESISTANCE IN RESIDENTS OF TWO REGIONAL CAPITALS IN GHANA: AN OBSERVATIONAL STUDY

--Manuscript Draft--

| | |
|---|---|
| **Manuscript Number:** | PGPH-D-24-00529R3 |
| **Article Type:** | Research Article |
| **Full Title:** | PREVALENCE AND DETERMINANTS OF INSULIN RESISTANCE IN RESIDENTS OF TWO REGIONAL CAPITALS IN GHANA: AN OBSERVATIONAL STUDY |
| **Short Title:** | PREVALENCE AND DETERMINANTS OF INSULIN RESISTANCE IN GHANA |
| **Corresponding Author:** | Samuel Acquah, Ph.D.<br>University of Cape Coast<br>Cape Coast, GHANA |
| **Order of Authors:** | Ansumana Sandy Bockarie, BSc, MBChB, MSc, FWCP |
| | Leonard Derkyi-Kwarteng, BSc, MBChB, MBA, MGP, PhD |
| | Jeffrey Amankona Obeng, BSc, MBChB |
| | Richard Kujo Adatsi, BSc, MPhil |
| | Ebenezer Aniakwaa-Bonsu, BSc, MPhil |
| | Charles Apprey, BSc, MSc, PhD |
| | Jerry Ampofo-Asiamah, BSc, MSc, PhD |
| | Samuel Acquah, BSc, MSc, MSc, PhD |
| **Keywords:** | COVID-19; insulin resistance; beta-cell function; type 2 diabetes mellitus; diabetogenic risk |
| **Abstract:** | Background: In spite of the effective control of the acute devastative effects of the coronavirus disease 2019 (COVID-19), the long-term impact has not been fully investigated. The current study sought to examine the determining factors of insulin resistance in resident Ghanaians in the Cape Coast and Tamale metropolis with or without COVID-19 exposure.<br>Methods: Using a cross-sectional design involving 510 adult participants, body mass index, waist-to-hip ratio, systolic blood pressure, lipid profile, insulin, plasma glucose, C-reactive protein, beta-cell function and insulin resistance levels were assessed and compared between 255 COVID-19 exposed and an equal number of unexposed residents. Insulin resistance was determined by the homeostatic model and the triglyceride-glucose index. Logistic regression was applied to identify determinants of insulin resistance in the two study groups.<br>Results: Prevalence of insulin resistance of insulin resistance ranged from 74.31% to 89.61% depending on the surrogate marker used coupled with low (31.18%) and high (19.41%) beta-cell function were observed. Additionally, varying degrees of dyslipidemias (8.31 % - 44.71%) and low-grade inflammation (50.59 %) were observed. Tertiary education, age 50-59 years, high levels of fasting plasma glucose, insulin and being overweight increased the odds of developing insulin resistance, but high beta-cell function and residing in Tamale offered protection against the condition in the COVID-19 unexposed participants only.<br>Conclusion: The risk of development type 2 diabetes mellitus through insulin resistance is high in our setting. Efforts at educating the adult populace to adopt lifestyle measures that reduce insulin resistance should be intensified to minimize the diabetogenic risk. |
| **Additional Information:** | |
| **Question** | **Response** |
| **Financial Disclosure** | All instructions have been duly reviewed and strictly adhered to, please. |

Powered by Editorial Manager® and ProduXion Manager® from Aries Systems Corporation

| | |
|---|---|
| Enter a financial disclosure statement that describes the sources of funding for the work included in this submission and the role the funder(s) played. This includes grants and any commercial funding of the work or authors.<br><br>This statement will be typeset if the manuscript is accepted for publication.<br><br>*Please review the submission guidelines and the instructions link below for detailed requirements and guidance.* | |
| **Competing Interests**<br><br>On behalf of all authors, disclose any competing interests that could be perceived to bias this work.<br><br>This statement will be typeset if the manuscript is accepted for publication.<br><br>*Please review the instructions link below and PLOS Global Public Health's competing interests policy to determine what information must be disclosed at submission.* | On behalf of all authors, I disclose that there is no competing interests. |
| **Data Availability**<br><br>Before publication, Authors are required to make fully available and without restriction all data underlying their findings. Please see our PLOS Data Policy page for detailed information on this policy.<br><br>A **Data Availability Statement**, detailing where the data can be accessed, is required at first submission. Insert your Data Availability Statement in the box below. | Every data used in the manuscript have been included in the manuscript, please. |

| | |
|---|---|
| Please see the data reporting section of our submission guidelines for instructions on what you need to include in your Data Availability Statement.<br><br>This statement will be typeset if the manuscript is accepted for publication.<br><br>*PLOS allows rare exemptions to address legal and ethical concerns. If you have legal or ethical restrictions, please use the box below to detail these in full sentences for the Journal team to consider.* | |

*Powered by Editorial Manager® and ProduXion Manager® from Aries Systems Corporation*

Manuscript

Click here to access/download;Manuscript;PLOS ONE - UNMARKED---13-2-25.docx

**Prevalence and Determinants of Insulin Resistance in Recovered COVID-19 and Uninfected Residents of Two Regional Capitals in Ghana: An Observational Study**

**[1]Ansumana Sandy Bockarie, [2]Leonard Derkyi-Kwarteng, [3]Jeffrey Amankona Obeng, [4]Richard Kujo Adatsi, [5]Ebenezer Aniakwaa-Bonsu, [6]Charles Apprey, [7]Jerry Ampofo Asiamah, [8]Samuel Acquah**

1. Department of Internal Medicine and Therapeutics, School of Medical Sciences, College of Health and Allied Sciences, University of Cape Coast.
2. Department of Pathology, School of Medical Sciences, College of Health and Allied Sciences, University of Cape Coast.
3. Cape Coast Teaching Hospital, Cape Coast.
4. Public Health Reference Laboratory, Tamale Teaching Hospital, Tamale.
5. Department of Microbiology and Immunology, School of Medical Sciences, College of Health and Allied Sciences, University of Cape Coast.
6. Department of Biochemistry and Biotechnology, School of Biosciences, College of Science, Kwame Nkrumah University of Science and Technology, Kumasi, Ghana.
7. Department of Biochemistry, School of Biological Science, College of Agricultural and Natural Sciences, University of Cape Coast
8. Department of Medical Biochemistry, School of Medical Sciences, College of Health and Allied Sciences, University of Cape Coast.

***Corresponding author**, Samuel Acquah, Department of Medical Biochemistry, School of Medical Sciences, College of Health and Allied Sciences, University of Cape Coast.

Email: sacquah@ucc.edu.gh

**ABSTRACT**

**Background:** The long-term impact of the coronavirus disease 2019 (COVID-19) pandemic on metabolic risk factors in different populations has not been fully investigated. Insulin resistance (IR) is a cardinal risk factor for the development of type 2 diabetes mellitus. The current study sought to determine the prevalence and determinants of insulin resistance in selected Ghanaians with and without past COVID-19 status in the Cape Coast and Tamale metropolitan areas.

**Methods:** Using a cross-sectional study design involving 510 adult participants, body mass index (BMI), waist-to-hip ratio, systolic blood pressure, lipid profile, insulin, plasma glucose, C-reactive protein (CRP), beta-cell function and insulin resistance levels were measured and compared between participants with and without past COVID-19 status. Insulin resistance (IR) was determined by the homeostatic model (HOMA-IR) and the triglyceride-glucose index (TyG). Percentage prevalence and Poisson regression with prevalence ratio and 95% confidence intervals were applied.

**Results:** IR prevalence ranged from 70.69% to 79.09% (HOMA-IR) and 88.62% to 90.91% (TyG) respectively for Tamale and Cape Coast residents. IR prevalence values of 70.98% and 88% (HOMA-IR) and 89.02% and 90.2% (TyG) for without and with past COVID-19 groups respectively were observed. Irrespective of background, low (31.18%) and high (19.41%) levels of beta-cell function were detected. Additionally, high levels of very-low density lipoprotein cholesterol (8.31%), triglycerides (24.9%), total cholesterol (27.45%), low-density lipoprotein cholesterol (44.71%) and low level of high-density lipoprotein cholesterol (11.96%) coupled with low-grade inflammation (50.59 %) were observed. Irrespective of surrogate marker used or past COVID-19 status, age, educational level and triglycerides could significantly associate with IR. With HOMA-IR, fasting plasma glucose, insulin and total cholesterol predicted IR in participants without prior COVID-19 status. With TyG, age, BMI, triglycerides and CRP were the predictors of IR in participants with past COVID-19 status.

**Conclusion:** The risk of development of type 2 diabetes mellitus through insulin resistance is high in our setting. Measures to reduce the rising pace of IR are urgently needed in our setting.

**Key words:** COVID-19, Insulin resistance, beta-cell function, type 2 diabetes mellitus, diabetogenic risk.

## INTRODUCTION

Although the devastation of acute coronavirus disease 2019 (COVID-19) on various populations and world health systems appear to have abated, its long-term effects on the health and wellbeing of the affected is still persisting in various forms.  Even in the sub-Saharan African region where the effect of the COVID-19 pandemic was not deemed as severe in terms of morbidity and mortality, the long-term impact on the populace cannot be underestimated. According to the latest data from the Ghana Health Service, the COVID-19 had affected 171,889 since its outbreak in 2020, as at March 2, 2023 with 70.8% of the population vaccinated against the condition [1].

Recent data from the International Diabetes Federation [2] still maintains that the sub-Saran African region has the highest prevalence of undiagnosed cases of diabetes and it is expected to experience the highest incidence of the condition by 2045. This gloomy prediction is hinged on predicted increased trend of obesity, driven by sedentary lifestyle and consumption of a westernized diet dominated by refined carbohydrates and saturated fats [2].  These diabetogenic predictions do not factor the probable role of infection-driven inflammation in the development of diabetes, especially, in the sub-Saharan African region. Indeed, the sub-Saharan African region continues to remain the hub of global infectious diseases [3]. It was probably based on this notion that the region was erroneously predicted to suffer the highest prevalence of mortality associated with acute COVID-19 infection in the early stages of the pandemic. In spite of the relatively mild short-term impact of COVID-19, in terms of morbidity and mortality on the regional populace, the probable long-term effects on the development of chronic non-communicable diseases in the region, especially, type 2 diabetes mellitus (T2DM), remains to be fully ascertained.

Interestingly, some studies have found that COVID-19 in people with diabetes increases diabetic complications and adverse outcomes of the viral condition [4-6]. Other studies [6-8] have associated the COVID-19 condition with new-onset diabetes of type 1 phenotype. Although T2DM constitutes more than 90% of global cases of diabetes, studies on the impact of COVID-19 on the condition post-recovery are limited.

Chen et al. [9] observed that six months after recovery from COVID-19, the risk of insulin resistance could increase. COVID-19-associated insulin resistance is thought to develop at several points of viral-host interactions and involves the integrated stress response (ISR) system [10]. The ISR includes a series of signaling pathways that are activated in response to various factors including obesity and viral infection and can result in impaired insulin action. Another important biochemical phenomenon that is central to the development of COVID-19-associated insulin resistance is

inflammation. Inflammation is a defense mechanism but can be harmful if dysregulated. Inflammation is linked to various health conditions including insulin resistance, diabetes, obesity, cancer and other chronic disease conditions [10, 11]. Interestingly, these conditions can also reinforce the inflammatory phenomenon.

Considering the critical role of insulin resistance in the pathogenesis of several chronic diseases in general and T2DM in particular, it is important to examine the promoting factors in our setting in order to identify appropriate mitigating measures to moderate its impact. This is critical because, insulin resistance, which is a cardinal risk factor for development of T2DM, can be influenced by several factors that may vary with the setting, making it difficult to adopt universal mitigating measures without recourse to the specific local context. Therefore, the current study aims to determine the prevalence and determinants of insulin resistance using two surrogate markers in a Ghanaian population that had recovered completely from COVID-19 and their counterparts that never had the condition in the Cape Coast and Tamale metropolitan areas.

## METHODS

### Study site, design and population

The study was conducted among residents of Cape Coast and Tamale metropolitan areas with the laboratory measurements taking place at the teaching hospitals of the two cities. The Cape Coast Teaching Hospital (CCTH) and the Tamale Teaching Hospital (TTH) are located in the Cape Coast and Tamale metropolis respectively. Both are tertiary facilities in the Ghanaian health care system and played a critical role in the management of COVID-19 cases during the pandemic. Whereas the CCTH is situated in the Central region along the coast of the Gulf of Guinea, TTH is located in Northern region of Ghana just below the Sahel belt. The two sites differ in geography, climate, ethnicity and dietary norms. The target population was the apparently healthy adults in the two metropolis. This cross-sectional study involved a total of 510 participants with 220 from Cape Coast and 290 from Tamale. The inclusion criteria were adults aged 20 years and above, apparently healthy, vaccinated against COVID-19 and without any underlying health condition at the time of recruitment. The participants who had recovered from COVID-19 had been declared healed from the condition by a negative PCR test results coupled with a clinical determination at least eight months before recruitment and were residents of either Tamale or Cape Coast and their surrounding communities. Those without a

history of the COVID-19 condition originated from the same communities as their recovered counterparts but were never diagnosed of the condition. Individuals who were pregnant, breastfeeding, diabetic, or had any health condition that could affect the levels of any of the blood-based measured indices were excluded from the study. The assessment of the inclusion and exclusion criteria was by effective administration of the appropriate questionnaires by trained health professionals. In addition, available health records of recovered COVID-19 were cross-checked with the responses of participants where appropriate. In both towns, workers in the formal sector of the Ghanaian economy dominated the participants. All participants had been vaccinated against the COVID-19 virus. Figure 1 shows a map of Ghana (https://www.ghanamissionun.org/wp-content/uploads/2023/11/Ghana_Regional_Map.jpg) with its administrative regional capitals including Cape Coast and Tamale where the study was conducted.

**Figure 1: Map of Ghana with its administrative capitals including the Cape Coast and Tamale metropolis**

***Determination of sample size***

The sample size was determined for each study site from the prevalence of COVID-19 at the national and regional levels by assuming the regional prevalence to be specific for the health facility of interest. Therefore, the regional prevalence of COVID-19 for the Central was assumed to be for the CCTH with that of the Northern region for the TTH, due to the critical roles that these tertiary health facilities played in the management of COVID-19 cases in their respective regions. The formula, $N = \{P1(1-P1) + P2(1-P2)\}\left(\frac{Z\alpha+Z\beta}{E}\right)^2$, [12] was applied to determine the sample size for each of the study sites. The estimated regional prevalence (P1) of COVID-19 were 3% and 0.15% for the Central and Northern regions respectively coupled with a national prevalence (P2) of 0.5%. However, the Central regional prevalence of the condition was applied across board in estimating the sample size in order to get adequate numbers for the study. Using the 3% regional and 0.5% national prevalence of COVID-19, coupled with a $Z_\alpha$ of 1.96

for a two-tailed test, $Z_\beta$ at 0.84 for 80% power and a margin of error (E) of 5%, the sample size was estimated to be 106 for each study group giving rise to 212 participants for the two groups in each study site. This implies that for both CCTH and TTH, a total of 424 participants would have been adequate for the study. Ultimately, in the current analysis, data from 510 participants made up of 220 from Cape Coast and 290 from Tamale were used.

***Selection of study participants***

The simple random sampling technique was used to recruit 255 participants with past COVID-19 status and an equal number without past COVID-19 status, for the study. Figure 2 outlines the selection of study participants at the two study sites. Participants were recruited between 1st July, 2022 and 30th June 2023 for the CCTH site. However, for the TTH arm, recruitment started from 1st March 2023 to 30th November, 2023. Since management of COVID-19 in Ghana was reserved for selected teaching hospitals including the CCTH and TTH, records of such individuals could be assessed and contacted for the study. Therefore, 150 and 200 recovered COVID-19 participants were randomly selected from the CCTH and TTH respectively and contacted by telephone calls for participation in the study. Similarly, 150 and 200 control participants were selected from the Cape Coast and the Tamale metropolitan areas in a random manner in line with the study protocol for participation. After appropriate screening to ensure compliance with inclusion and exclusion criteria, 220 and 290 participants from the Cape Coast and Temale metropolitan areas respectively were recruited for the study. Details of the recruitment are shown below in figure 2.

**Figure 2: Flow Chart for Participants' Selection**

**Assessment of Study Variables**

To achieve the objectives of the study, the following variables of demographic, anthropometric and biochemical relevance were assessed.

***Demographic variables***

Demographic information including age, education, past COVID-19 status, smoking and alcohol consumption status were collected by a structured questionnaire designed for the study.

***Blood pressure and anthropometric measurements***

An experienced nurse measured the blood pressure of the participant with an electronic sphygmomanometer (Contec Medical Systems Co. Ltd, China) in a routine manner. The blood pressure was measured on the right arm of participants in sitting position after a minimum of five minutes rest. An average of two measurements that did not differ by >5 mmHg was recorded for each participant as the blood pressure.

Anthropometric indices such as weight (kg), height (m), waist circumference (cm) and hip circumference (cm) were measured for computation of waist-to-hip ratio and body mass index (BMI). Weight was measured to the nearest 0.1 kg with height to the nearest 0.1 cm. BMI was calculated by dividing the weight in kilogramme by the square of the height in metre ($kg/m^2$). Weight and height were measured in light clothing without footwear. Weight was measured by an electronic scale and the height by stadiometer (Seca® mechanical column scale with stadiometer, USA). Waist and hip circumferences were measured in centimetres with an inflexible tape measure. Waist circumference was measured at the midpoint between the lower margin of the last rib and the top of the iliac crest but the hip circumference was measured around the widest portion of the buttocks. Waist-to-hip ratio was obtained by dividing the waist circumference by the hip circumference.

***Measurement of glucose, lipid profile, insulin and C-reactive protein levels***

Measurements of plasma glucose and lipid profile including LDL, HDL, total cholesterol, triglyceride and VLDL were done with the Mindray BS240 automated chemistry analyser (Mindray Diagnostics, Nanshan Shenzhen, China) following a routine procedure.

However, serum insulin and C-reactive protein (CRP) levels were determined by immunoturbidimetric test kits purchased from Kamiya Biomedicals Company (K-ASSAY Seattle, USA) in accordance with the manufacturer's detailed instructions outlined by Adatsi et al. [13].

***Assessment of insulin resistance and beta-cell function***

Insulin resistance was assessed by the homeostatic model, $HOMA-IR = Glucose\left(\frac{mmol}{l}\right) * Insulin\,(m\frac{U}{l})/22.5$ [14] and the triglyceride-glucose (TyG) index, $TyG = In\left[FBS\left(\frac{mg}{dl}\right) * TG\left(\frac{mg}{dl}\right)\right]/2$ [15]. Additionally, beta-cell function was determined using the homeostatic model, $HOMAB = 20 * Fasting\ Insulin\,(\frac{mU}{l})/ Fasting\ Glucose\,(\frac{mmol}{l}) - 3.5$ developed by Matthews et al. [14].

**Determination of abnormal levels of blood-based parameters**

Dyslipidemias was assessed in accordance with the National Cholesterol Education Program Adult Treatment Panel (NCEP-ATP) III guidelines [16]. Specifically, abnormal levels of the lipid indices were defined as triglycerides ≥ 1.7 mmol/l; low-density lipoprotein cholesterol (LDL) ≥ 2.6 mmol/l; high-density lipoprotein cholesterol (HDL) < 1.03 mmol/l; total cholesterol ≥ 5.2 mmol/l. In addition, high levels of the other indices were defined as glucose > 6.4 mmol/l; insulin > 24.9 mIU/l; insulin resistance, HOMA-IR > 2.6 or TyG index > 4.0; beta-cell function, HOMA-B, high > 100 % or low < 25 %.

**Ethical Approval**

The Ethical Review Committee of the Tamale Teaching Hospital (TTHERC/24/02/23/01) granted approval for the study at Tamale as Cape Coast Teaching Hospital (CCTHERC/EC/2022/111) approved that for the Cape Coast arm. A written informed consent was provided by each study participant and at all times, the study protocols adhered strictly to the ethical standards of the Ghana Health Service and the World Medical Association Declaration of Helsinki.

**Data analysis**

Statistical Software for Data Science (STATA) version 15 Corp (StataCorp LLC, USA) software was used to analyze the data for this study. Demographic characteristics and other categorical variables

were presented in frequencies and percentages and compared with the chis-square goodness of fit test. Continuous variables were presented as mean and standard deviation if normally distributed or median and interquartile ranges if distribution deviated from normal distribution. The independent sample t-test was used to compare mean levels of measured indices between participants with and without past COVID-19 status for continuous variables that met assumptions for parametric test else the Mann Whitney test was used. Poisson regression with prevalence ratio was used to examine the relationship between insulin resistance, measured by HOMAIR or TyG, as the dependent variable and the other predictor variables. The univariate followed by the multivariate regression analyses were applied. Before fitting the multivariate regression model, the variance inflation factor (VIF) and tolerance (1/VIF) were used to assess multicollinearity among the independent variables, and no evidence of collinearity was found. The results of the regression analyses were presented as crude prevalence ratios (CPRs) and adjusted prevalence ratios (APRs) with 95% confidence intervals (CIs) and corresponding p-values. The determination of statistical significance for all analyses was considered at $p < 0.05$. Effect size (Cohen’s d) was computed for the adjusted models and the mean/median comparisons to examine the strengths of the relationships. The standard interpretations for effect size were adopted in interpreting our data.

## RESULTS

In the current study, two different surrogate markers (HOMA-IR and TyG) were used for the assessment of insulin resistance to allow for probable identification of variety of promoters of the condition in our context. Various demographic, anthropometric and blood-based indices known to be associated with insulin resistance in various populations were assessed in the current study that involved 510 residents of Tamale and Cape Coast metropolis.

Data from 290 participants from Tamale (150 females and 140 males) and 220 participants from Cape Coast (123 males and 97 females) were used in our analysis.

### *Sociodemographic factors*

The sociodemographic characteristics of participants (table 1) indicate that a significant ($P < 0.05$; Table 1) majority of the participants were residents of Tamale (56.86%), aged 20-39 years (69.02%), had tertiary education (65.29%),

non-smokers (95.88%) and did not drink alcohol (76.08%). In terms of gender, proportions of male and female were similar statistically (P = 0.48; Table 1).

**Table 1: Sociodemographic characteristics of participants**

| Parameter | Frequency (%) | P-value |
|---|---|---|
| **Location** | | 0.002* |
| Cape Coast | 220 (43.14) | |
| Tamale | 290 (56.86) | |
| **Gender** | | 0.48 |
| Female | 247 (48.43) | |
| Male | 263 (51.57) | |
| **Age** | | < 0.001* |
| 20-29 | 189 (37.06) | |
| 30-39 | 163 (31.96) | |
| 40-49 | 92 (18.04) | |
| 50-59 | 36 (7.06) | |
| 60 and above | 30 (5.88) | |
| **Education** | | < 0.001* |
| No education | 75 (14.71) | |
| Primary | 24 (4.71) | |
| Secondary | 81 (15.88) | |
| Tertiary | 330 (64.71) | |
| **Past COVID-19** | | 1.00 |
| No | 255 (50) | |
| Yes | 255 (50) | |
| **Smoking status** | | < 0.001* |
| No | 489 (95.88) | |
| Yes | 21 (4.12) | |
| **Alcohol consumption** | | < 0.001* |
| No | 388 (76.08) | |
| Yes | 122 (23.92) | |

**COVID-19:** Coronavirus disease 2019; ***:** Significant p-value for chi-square goodness of fit test

### *Mean/median levels of measured indices*

Compared to the uninfected group, the recovered COVID-19 participants had higher (P < 0.05) levels of waist circumference, hip circumference, fasting plasma glucose, insulin, low-density lipoprotein cholesterol, total cholesterol, HOMA-IR and C-reactive protein (Table 2) although the observed differences were generally small.

**Table 2: Levels of measured parameters by COVID-19 status**

| Parameter | Entire participants Mean ± SD | COVID-19 uninfected Mean ± SD | Recovered COVID-19 Mean ± SD | P-value | Effect size (Cohen's d) |
|---|---|---|---|---|---|
| Weight (kg) | 71.73 ± 14.28 | 70.64 ± 14.60 | 72.83 ± 13.89 | 0.08 | 0.15 |
| Height (m) | 1.68 ± 0.09 | 1.68 ± 0.10 | 1.68 ± 0.08 | 0.87 | 0.01 |
| Waist circumference (cm) | 82.61 ± 11.67 | 81.46 ± 11.92 | 83.75 ± 11.33 | 0.03* | 0.20 |
| Hip circumference (cm) | 95.80 ± 13.06 | 94.31 ± 0.85 | 97.28 ± 0.79 | 0.01* | 0.23 |
| WHR | 0.86 ± 0.06 | 0.87 ± 0.06 | 0.86 ± 0.06 | 0.52 | 0.06 |
| Systolic BP (mmHg) | 126.33 ± 14.83 | 126.24 ± 15.47 | 126.41 ± 14.19 | 0.9 | 0.01 |
| BMI (kg/m$^2$) | 25.43 ± 5.07 | 25.12 ± 4.98 | 25.75 ± 5.14 | 0.16 | 0.15 |
| FPG (mmol/L) | 4.74 ± 0.95 | 4.53 ± 0.81 | 4.94 ± 1.04 | <0.001* | 0.44 |
| | **Median (IQR)** | **Median (IQR)** | **Median (IQR)** | | |
| Insulin (mIU/L) | 18.45 (13.4-27.5) | 17.80 (12.40-23.40) | 19.4 (14.1-45.6) | <0.001* | 0.17 |
| HOMA-B (%) | 115.93 (68.9-295.60) | 111.71 (66.09-253.04) | 127.19 (69.57-372.50) | 0.12 | 0.07 |
| HOMAIR (mIU/L) | 3.72 (2.57-5.99) | 3.43 (2.47-4.74) | 4.25 (2.78-10.63) | <0.001* | 0.20 |
| TyG index | 4.46 (4.26-4.71) | 4.45 (4.25-4.66) | 4.49 (4.33-4.73) | 0.26 | 0.05 |
| HDL (mmol/L) | 1.73 (1.32-2.45) | 1.70 (1.32-2.33) | 1.74 (1.29-2.59) | 0.39 | 0.04 |
| LDL (mmol/L) | 2.46 (1.62-3.48) | 2.30 (1.48-3.38) | 2.81 (1.78-3.54) | 0.03* | 0.10 |
| VLDL(mmol/L) | 0.43 (0.33-0.7) | 0.39 (0.33-0.66) | 0.46 (3.13-72.4) | 0.9 | 0.07 |
| Total cholesterol (mmol/L) | 4.42 (3.24-5.32) | 4.21 (3.02-5.32) | 4.56 (3.67-5.32) | 0.04* | 0.10 |
| Triglyceride (mmol/L) | 0.99 (0.71-1.62) | 1.01 (0.72-1.52) | 0.97 (0.71-1.69) | 0.74 | 0.02 |
| CRP (mg/dL) | 1.00 (0.4-1.4) | 0.80 (0.30-1.20) | 1.20 (0.70-1.78) | <0.001* | 0.26 |

**CRP:** C-reactive protein; **BMI:** Body mass index; **FPG:** Fasting plasma glucose; **HDL:** High-density lipoprotein cholesterol; **VLDL:** Very low-density lipoprotein cholesterol; **HOMA-IR:** Homeostatic model assessment of insulin resistance; **HOMAB:** Homeostatic model of beta-cell function; **BP:** Blood pressure; **WHR:** Weight-to-height ratio; **SD:** Standard deviation; **IQR:** Inter-quartile range; **COVID-19:** Coronavirus disease 2019; *****: Significant p-value for independent sample t-test (mean) or Mann Whitney test (median).

***Abnormal levels of measured variables***

Varied proportions (Figure 3) of abnormal levels of the measured indices were observed among the study participants. For example, the prevalence of insulin resistance (IR) ranged from 70.69% (Tamale) to 79.09% (Cape Coast) for the HOMA-IR index with an overall rate of 74.31% (Table 3 and figure 3). With TyG index, the observed IR prevalence were 88.62% and 90.91% (Table 4) respectively for the Tamale and Cape Coast residents with an overall rate of 89.61% (figure 3). However, the respective IR prevalence of 70.98% and 88% (HOMA-IR; Table 3) and 89.02% and 90.2% (TyG; Table 4) for participants without past COVID-19 status and those with past COVID-19 status were observed. Irrespective of location or COVID-19 status, low (31.18%) and high (19.41%) levels of beta-cell function were also observed (figure 3). In terms of dyslipidemias, prevalence values of 8.43 % for very low-density lipoprotein

cholesterol, high levels of very-low density lipoprotein cholesterol (8.31%), triglycerides (24.9%), total cholesterol (27.45%), low-density lipoprotein cholesterol (44.71%) and low level of high-density lipoprotein cholesterol (11.96%) and low-grade inflammation (50.59 %) were noted (figure 3).

**Figure 3: Prevalence of abnormal levels of blood-based indices**

*Except otherwise indicated, all the proportions represent percentage of individuals with levels of measured indices above the upper threshold of the reference range apart from HDL which represents a level below the lower threshold of its reference range.* ***CRP:*** *C-reactive protein;* ***BMI:*** *Body mass index;* ***FPG:*** *Fasting plasma glucose;* ***HDL:*** *High-density lipoprotein cholesterol;* ***VLDL:*** *Very low-density lipoprotein cholesterol;* ***HOMA-IR:*** *Homeostatic model assessment of insulin resistance;* ***HOMAB:*** *Homeostatic model of beta-cell function;* ***LDL:*** *low-density lipoprotein cholesterol;* ***TyG:*** *triglyceride-glucose index*

***Predictors of insulin resistance determined by HOMA-IR***

In a regression analysis to identify predictors of insulin resistance using HOMA-IR as the dependent variable in the entire sample irrespective of past COVID-19 status, age, education and triglycerides came out as the only predictors of insulin resistance (Table 3). Residing in Tamale decreased the prevalence of insulin resistance by 11% (PR: 0.89; CI: 0.81-0.99; P = 0.03; Table 3) compared to being a resident of Cape Coast in a univariate analysis. However, this reduction could not be sustained after adjusting for confounders (PR: 0.96; CI: 0.91-1.02; P = 0.23; Table 3**)** in a multivariate analysis although 79.09% (174/220) of Cape Coast residents and 70.69% (205/290) of Tamale residents were insulin resistant. Compared with age 20-29 years, participants in age group 30-39 and 50-59 years had respective increased prevalence of 18% (PR: 1.18; CI: 1.03-1.33; P = 0.01; Table 3) and 27% (PR: 1.27; CI: 1.07-1.50; P = 0.004; Table 3) in having insulin resistance with no such effect observed for those in age group 40-49 or 60 years and above. After controlling for confounders, only the participants in age group 50-59 years demonstrated 11% increase in prevalence of insulin resistance (PR: 1.11; CI: 1.02-1.21; P = 0.01; Table 3). The strength of the observed associations ranged from small (30-39 years group) to medium (50-59 years group). Having high glucose level did not demonstrate statistically significant association with insulin resistance although high triglycerides increased the prevalence by 5% (PR: 1.05; CI: 1.00-1.11; P = 0.04; Table 3) with medium strength of the observed association. Interestingly, the prevalence of insulin resistance increased by 13% (PR: 1.13; CI: 1.01-1.27; P = 0.03; Table 3) in participants with secondary education but decreased by 98% (PR: 0.02; CI: 0.002-0.14; P < 0.001; Table 3) in those with tertiary education in our setting. Indeed, across levels, the strength of the association between insulin resistance and formal education in general, was large in our context. Statistically, neither participants with high insulin levels (PR: 1.18; CI: 0.75-1.85; P = 0.46; Table 3) nor past

COVID-19 status (PR: 0.98; CI: 0.92-1.04; P = 0.56; Table 3) demonstrated increased prevalence of insulin resistance compared to their counterparts with normal levels of the parameter or without past COVID-19 status in our context.

**Table 3: Predictors of insulin resistance determined by HOMA-IR**

| Parameter | No insulin resistance, N (%) | Insulin resistance, N (%) | CPR (95% CI) | P-value | APR (95%CI) | P-value | Effect size (Cohen's d) |
|---|---|---|---|---|---|---|---|
| **Location** | | | | | | | |
| Cape Coast (ref.) | 46 (35.11) | 174 (45.91) | 1 | | 1 | | |
| Tamale | 85 (64.89) | 205 (54.09) | 0.89(0.81-0.99) | 0.03* | 0.96(0.91-1.02) | 0.23 | -0.24 |
| Gender | | | | | | | |
| Female (ref.) | 57 (43.51) | 190 (50.13) | 1 | | 1 | | |
| Male | 74 (56.49) | 189 (49.87) | 0.93(0.84-1.03) | 0.19 | 1.04(0.99-1.10) | 0.11 | -0.18 |
| **Age** | | | | | | | |
| 20-29 (ref) | 61 (46.57) | 128 (33.77) | 1 | | 1 | | |
| 30-39 | 33 (25.19) | 130 (34.30) | 1.18(1.03-1.33) | 0.01* | 1.03(0.96-1.10) | 0.35 | 0.29 |
| 40-49 | 22 (16.79) | 70 (18.47) | 1.12(0.97-1.31) | 0.15 | 1.06(0.98-1.15) | 0.2 | 0.21 |
| 50-59 | 5 (3.82) | 31 (8.18) | 1.27(1.07-1.50) | 0.004* | 1.11(1.02-1.21) | 0.01* | 0.64 |
| 60 and above | 10 (7.63) | 20 (5.28) | 0.98(0.75-1.29) | 0.91 | 0.97(0.84-1.12) | 0.71 | -0.09 |
| **Education** | | | | | | | |
| No education (ref.) | 1 (0.76) | 75 (19.79) | 1 | | 1 | | |
| Primary | 3 (2.29) | 20 (5.28) | 0.73(0.51-1.06) | 0.11 | 1.03(0.85-1.26) | 0.73 | -1.08 |
| Secondary | 9 (6.87) | 72 (18.99) | 0.83(0.69-1.00) | 0.06 | 1.13(1.01-1.27) | 0.03* | -1.17 |
| Tertiary | 118 (90.08) | 212 (55.94) | 0.95(0.83-1.08) | 0.44 | 0.02(0.002-0.14) | <0.001* | -2.16 |
| **Smoke** | | | | | | | |
| No (ref.) | 126 (96.18) | 363 (95.78) | 1 | | 1 | | |
| Yes | 5 (3.82) | 16 (4.22) | 1.02(0.80-1.31) | 0.84 | 0.96(0.83-1.11) | 0.61 | 0.07 |
| **Past COVID-19** | | | | | | | |
| No (ref.) | 74 (56.49) | 181 (47.76) | 1 | | 1 | | |
| Yes | 57 (43.51) | 198 (52.24) | 1.09(0.98-1.21) | 0.09 | 0.98(0.92-1.04) | 0.56 | 0.23 |
| **HOMA-B** | | | | | | | |
| Normal (ref.) | 7 (5.34) | 245 (64.64) | 1 | | 1 | | |
| High | 38 (29.01) | 61 (16.09) | 1.45(0.76-1.56) | 0.39 | 1.08(0.18-1.29) | 0.15 | -0.39 |
| Low | 86 (65.65) | 73 (19.26) | 0.99(0.89-1.20) | 0.67 | 0.98(0.70-1.14) | 0.23 | -1.65 |

| | | | | | | | |
|---|---|---|---|---|---|---|---|
| **Waist-to-hip ratio** | | | | | | | |
| Normal (ref.) | 71 (54.20) | 219 (57.78) | 1 | | 1 | | |
| High | 60 (45.80) | 160 (42.22) | 0.91 (0.61-1.36) | 0.63 | 0.86(0.57-1.31) | 0.49 | -0.08 |
| **Waist circumference** | | | | | | | |
| Normal (ref.) | 108 (82.44) | 321 (84.70) | 1 | | 1 | | |
| High | 23 (17.56) | 58 (15.30) | 0.96(0.82-1.11) | 0.59 | 0.93(0.85-1.02) | 0.14 | -0.13 |
| **BMI** | | | | | | | |
| Normal (ref.) | 100 (76.34) | 210 (55.41) | 1 | | 1 | | |
| Underweight | 10 (7.63) | 15 (3.96) | 0.91(0.64-1.30) | 0.62 | 1.01(0.79-1.31) | 0.87 | 0.18 |
| Overweight | 16 (12.21) | 115 (30.34) | 1.02(0.71-1.46) | 0.90 | 1.09(0.85-1.40) | 0.47 | 0.22 |
| Obese | 5 (3.82) | 39 (10.29) | 0.94(0.63-1.39) | 0.74 | 1.16(0.90-1.51) | 0.23 | 0.06 |
| **FPG** | | | | | | | |
| Normal (ref.) | 129 (92.37) | 363 (94.72) | 1 | | 1 | | |
| High | 2 (1.53) | 16 (4.22) | 2.67 (1.18-6.03) | 0.02* | 1.32(0.90-1.94) | 0.15 | 0.68 |
| **Insulin** | | | | | | | |
| Normal (ref.) | 130 (99.24) | 231 (60.95) | 1 | | 1 | | |
| High | 1 (0.76) | 148 (39.05) | 1.23(0.78-1.89 | 0.45 | 1.18(0.75-1.85) | 0.46 | 2.43 |
| **LDL** | | | | | | | |
| Normal (ref) | 82 (62.60) | 200 (52.77) | 1 | | 1 | | |
| High | 50 (37.40) | 179 (47.23) | 1.04 (0.93-1.17) | 0.48 | 0.96(0.89-1.02) | 0.24 | 0.07 |
| **HDL** | | | | | | | |
| Normal | 114 (87.02) | 336 (88.65) | 1 | | 1 | | |
| Low | 17 (12.98) | 43 (11.35) | 1.15 (0.63-2.09) | 0.65 | 1.14(0.61-2.13) | 0.67 | 0.07 |
| **VLDL** | | | | | | | |
| Normal (ref.) | 120 (91.60) | 347 (91.56) | 1 | | 1 | | |
| High | 11 (8.40) | 32 (8.44) | 1.00(0.83-1.20) | 0.96 | 0.97(0.91-1.04) | 0.43 | -0.02 |
| **Cholesterol** | | | | | | | |
| Normal (ref.) | 96 (73.28) | 274 (72.30) | 1 | | 1 | | |
| High | 35 (26.72) | 105 (27.70) | 1.01 (0.91-1.14) | 0.77 | 1.04(0.98-1.10) | 0.18 | 0.02 |
| **Triglyceride** | | | | | | | |
| Normal (ref.) | 103 (78.63) | 17 (4.49) | 1 | | 1 | | |
| High | 28 (21.37) | 362 (95.51) | 1.28(0.79-2.06) | 0.32 | 1.05(1.00-1.11) | 0.04* | 0.51 |
| **CRP** | | | | | | | |
| Normal (ref.) | 70 (53.44) | 182 (48.02) | 1 | | 1 | | |
| High | 61 (46.56) | 197 (51.19) | 1.05(0.95-1.17) | 0.32 | 0.95(0.88-1.03) | 0.24 | 0.13 |

**CRP:** C-reactive protein; **BMI:** Body mass index; **FPG:** Fasting plasma glucose; **HDL:** High-density lipoprotein cholesterol; **VLDL:** Very low-density lipoprotein cholesterol; **HOMA-IR:** Homeostatic model assessment of insulin resistance; **HOMA-B:** Homeostatic model of beta-cell function; **WHR:** Weight-to-height ratio; **N:** Number of participants; **COVID-19:** Coronavirus disease 2019; **CPR:** Crude prevalence ratio (univariate); **APR:** Adjusted prevalence ratio (multivariate); ***:** Significant p-value

***Predictors of insulin resistance determined by TyG***

In a similar analysis with TyG (triglyceride-glucose index) as the measure of insulin resistance, age, education and triglycerides (P < 0.05; Table 4) were the only parameters that could predict insulin resistance with the strength of the association ranging from small (education) to medium (age and triglycerides). Participant in age group 50-59 years showed a 12% increased prevalence of insulin resistance (PR: 1.12; CI: 1.02-1.21; P = 0.01; Table 4) with medium strength of the observed association. Similarly, those with tertiary level educational attainment had a 12% increased prevalence (PR: 1.12; CI: 1.01-1.26; P = 0.04; Table 4) of insulin resistance but the strength of the observed association was small. The prevalence of insulin resistance increased by only 6% (PR: 1.06; CI: 1.00-1.11; P = 0.04; Table 4) for participants with high triglycerides levels although the association demonstrated a medium strength. Interestingly, as shown in Table 4, high levels of beta-cell function (PR: 0.97; CI: 0.942-1.76; P = 0.8), FPG (PR: 1.32; CI: 0.91-1.94; P = 0.15), CRP (PR: 0.96; CI: 0.88-1.03; P = 0.24); insulin (PR: 1.16; CI: 0.74-1.83; P = 0.52), and past COVID-19 status (PR: 0.98; CI: 0.92-1.05; 0.56) did not reveal any statistically significant association with insulin resistance.

**Table 4: Predictors of insulin resistance determined by triglyceride-glucose (TyG) index**

| **Parameter** | **No insulin resistance, N (%)** | **Insulin resistance, N (%)** | **CPR (95%CI)** | **P-value** | **APR (95%CI)** | **P-value** | **Effect size (Cohen's d)** |
|---|---|---|---|---|---|---|---|
| **Location** | | | | | | | |
| Cape Coast (ref.) | 20 (37.74) | 200 (43.76) | 1 | | 1 | | |
| Tamale | 33 (62.26) | 257 (56.24) | 0.95(0.90-1.01) | 0.08 | 0.96(0.91-1.02) | 0.23 | -0.47 |
| **Sex** | | | | | | | |
| Female (ref.) | 30 (56.60) | 217 (47.48) | 1 | | 1 | | |
| Male | 23 (43.40) | 240 (52.52) | 1.02(0.97-1.09) | 0.30 | 1.05(0.91-1.10) | 0.11 | 0.27 |
| **Age** | | | | | | | |
| 20-29 (ref) | 22 (41.51) | 167 (36.54) | 1 | | 1 | | |
| 30-39 | 12 (22.64) | 151 (33.04) | 1.06(0.99-1.13) | 0.10 | 1.03(0.96-1.11) | 0.96 | 0.19 |
| 40-49 | 7 (13.21) | 85 (18.60) | 1.07(0.99-1.16) | 0.06 | 1.07(0.98-1.16) | 0.18 | 0.25 |
| 50-59 | 8 (15.09) | 28 (6.13) | 1.11(1.02-1.19) | 0.01* | 1.12(1.02-1.21) | 0.01* | 0.50 |
| 60 and above | 4 (7.55) | 26 (5.69) | 0.98(0.84-1.15) | 0.85 | 0.97(0.84-1.13) | 0.71 | -0.33 |
| **Education** | | | | | | | |
| No education (ref.) | 7 (13.21) | 69 (15.10) | 1 | | 1 | | |
| Primary | 7 (13.21) | 16 (3.50) | 1.02(0.84-1.23) | 0.87 | 1.03(0.85-1.26) | 0.73 | 0.03 |
| Secondary | 12 (22.64) | 69 (15.10) | 1.12(1.00-1.25) | 0.0.04* | 1.13(1.01-1.27) | 0.23 | 0.45 |
| Tertiary | 27 (50.94) | 303 (66.30) | 1.09(0.98-3.02) | 0.11 | 1.12(1.01-1.26) | 0.04* | 0.07 |

| | | | | | | | |
|---|---|---|---|---|---|---|---|
| **Smoke** | | | | | | | |
| No (ref.) | 51 (96.23) | 438 (95.84) | 1 | | 1 | | |
| Yes | 2 (3.77) | 19 (4.16) | 0.99(0.89-1.14) | 0.77 | 0.96(0.83-1.11) | 0.61 | -0.06 |
| **Past Covid-19** | | | | | | | |
| No (ref.) | 25 (47.17) | 230 (50.33) | 1 | | 1 | | |
| Yes | 28 (52.83) | 227 (49.67) | 1.00(0.95-1.06) | 0.87 | 0.98(0.92-1.05) | 0.56 | -0.33 |
| **HOMA-B** | | | | | | | |
| Normal (ref.) | 31 (58.49) | 221 (48.36) | 1 | | 1 | | |
| High | 10 (18.87) | 89 (19.47) | 1.11(0.99-1.24) | 0.06 | 0.97(0.942-1.76) | 0.8 | -0.49 |
| Low | 12 (22.64) | 147 (32.17) | 1.09(0.97-1.22) | 0.14 | 0.96(0.94-1.21) | 0.32 | -0.11 |
| **Waist-to-hip ratio** | | | | | | | |
| Normal (ref.) | 35 (66.04) | 261 (57.11) | 1 | | 1 | | |
| High | 18 (33.96) | 196 (42.89) | 1.12(0.98-1.09) | 0.20 | 0.92(0.91-1.21) | 0.61 | 0.01 |
| **Waist circumference** | | | | | | | |
| Normal (ref.) | 48 (90.57) | 381 (83.37) | 1 | | 1 | | |
| High | 5 (9.43) | 76 (16.63) | 1.03(0.96-1.10) | 0.38 | 0.93(0.85-1.02) | 0.14 | -0.68 |
| **BMI** | | | | | | | |
| Normal (ref.) | 15 (2.56) | 295 (64.55) | 1 | | 1 | | |
| Underweight | 14 (74.36) | 11 (2.41) | 0.99(0.78-1.25) | 0.93 | 1.02(0.80-1.31) | 0.88 | 0.2 |
| Overweight | 19 (17.95) | 109 (23.85) | 1.07(0.85-1.36) | 0.55 | 1.09(0.85-1.40) | 0.48 | -0.01 |
| Obese | 5 (5.13) | 42 (9.19) | 1.07(0.84-1.36) | 0.57 | 1.17(0.90-1.51) | 0.24 | 0.56 |
| **FPG** | | | | | | | |
| Normal (ref.) | 53 (92.45) | 439 (96.06) | 1 | | 1 | | |
| High | 0 (0.00) | 18 (3.94) | 1.42(0.93-2.15) | 0.10 | 1.32(0.91-1.94) | 0.15 | 0.47 |
| **Insulin** | | | | | | | |
| Normal (ref.) | 52 (98.11) | 309 (67.62) | 1 | | 1 | | |
| High | 1 (1.89) | 148 (32.38) | 1.21(0.68-2.13) | 0.65 | 1.16(0.74-1.83) | 0.52 | 1.21 |
| **LDL** | | | | | | | |
| Normal (ref) | 39 (73.59) | 243 (72.87) | 1 | | 1 | | |
| High | 14 (26.41) | 214 (27.13) | 1.03(0.97-1.09) | 0.36 | 0.96(0.89-1.03) | 0.25 | 0.22 |
| **HDL** | | | | | | | |
| Normal | 47 (88.68) | 402 (87.96) | 1 | | 1 | | |
| Low | 6 (11.32) | 55 (12.04) | 0.74 (0.30-1.14) | 0.48 | 0.45 (0.21-1.10) | 0.28 | -0.32 |
| **VLDL** | | | | | | | |
| Normal (ref.) | 42 (71.79) | 425 (93.00) | 1 | | 1 | | |
| High | 11 (28.21) | 32 (7.00) | 1.04(0.97-1.13) | 0.22 | 1.04(0.91-1.04) | 0.43 | 0.02 |
| **Total cholesterol** | | | | | | | |

| | | | | | | | |
|---|---|---|---|---|---|---|---|
| Normal (ref.) | 18 (10.26) | 352 (70.46) | 1 | | 1 | | |
| High | 35 (89.74) | 105 (29.54) | 1.07(1.03-1.13) | 0.002 | 1.04(0.98-1.11) | 0.18 | 0.68 |
| **Triglyceride** | | | | | | | |
| Normal (ref.) | 39 (73.59) | 344 (75.27) | 1 | | 1 | | |
| High | 14 (26.41) | 113 (24.73) | 1.07(1.02-1.12) | 0.03* | 1.06(1.00-1.11) | 0.04* | 0.63 |
| **CRP** | | | | | | | |
| Normal (ref.) | 19 (35.85) | 233 (50.98) | 1 | | 1 | | |
| High | 34 (64.15) | 224 (49.02) | 1.07(1.03-1.010) | <0.001 | 0.96(0.88-1.03) | 0.24 | -0.49 |

**CRP:** C-reactive protein; **BMI:** Body mass index; **FPG:** Fasting plasma glucose; **HDL:** High-density lipoprotein cholesterol; **VLDL:** Very low-density lipoprotein cholesterol; **HOMA-IR:** Homeostatic model assessment of insulin resistance; **HOMA-B:** Homeostatic model of beta-cell function; **WHR:** Weight-to-height ratio; **N:** Number of participants; **COVID-19:** Coronavirus disease 2019; **CPR:** Crude prevalence ratio (univariate); **APR:** Adjusted prevalence ratio (multivariate); ***:** Significant p-value

***Predictors of insulin resistance measured by HOMA-IR according to past COVID-19 status***

In a sub-group analysis based on past COVID-19 status, only the levels of fasting plasma glucose, insulin and total cholesterol could significantly (P < 0.05; Table 5) associate with insulin resistance in participants without past COVID-19 status. Participants with high levels of plasma glucose (PR: 2.06; 1.03-4.10; P = 0.03; Table 5) and insulin (PR: 1.57; CI: 1.38-1.79; P < 0.001; Table 5) exhibited 106% and 57% respective increased prevalence of insulin resistance compared with their counterparts with normal levels of the parameters. The strengths of the observed associations ranged from medium (insulin) to high (FPG). However, participants with high total cholesterol level showed a 24% reduction in prevalence of insulin resistance (PR: 0.76; CI: 0.61-0.95; P = 0.01; Table 5) with a small strength of the observed association. The seeming 19% reduction in prevalence of insulin resistance by residing in Tamale (PR: 0.81; CI: 0.70-0.95; P = 0.01) observed in the univariate analysis in participants without past COVID-19 status could not be sustained after controlling for confounders in the multivariate analysis (PR: 0.87; CI: 0.72-1.07; P = 0.19; Table 5). Compared to age 20-29 years, age 30-39 years had a 124% increased prevalence of insulin resistance (PR: 2.24, CI: 1.12-4.49; P = 0.02; Table 5) in participants without past COVID-19 status but this effect vanished after adjustment (APR: 1.13; CI: 0.94-1.30; P = 0.25; Table 5). Interestingly, none of the measured indices exhibited a statistically significant (P > 0.05; Table 5) association with insulin resistance in the participants with past COVID-19 status.

**Table 5: Predictors of insulin resistance measured by HOMA-IR according to past COVID-19 status**

| Parameter | PAST COVID-19 STATUS | | | | | NO PAST COVID-19 STATUS | | | | |
|---|---|---|---|---|---|---|---|---|---|---|
| | CPR (95%CI) | P-value | APR (95%CI) | P-value | ES | CPR (95%CI) | P-value | APR (95%CI) | P-value | ES |
| **Location** | | | | | | | | | | |
| Cape Coast **(ref.)** | 1 | | 1 | | | 1 | | 1 | | |
| Tamale | 0.96(0.84-1.10) | 0.63 | 0.99(0.87-1.13) | 0.95 | 0.20 | 0.81(0.70-0.95) | 0.01* | 0.87(0.72-1.07) | 0.19 | 0.35 |
| **Sex** | | | | | | | | | | |
| Females (ref.) | 1 | | 1 | | | 1 | | 1 | | |
| Males | 0.92(0.81-1.05) | 0.23 | 0.97(0.85-1.11) | 0.69 | 0.34 | 0.93(0.80-1.09) | 0.41 | 0.89(0.76-1.04) | 0.14 | 0.17 |
| **Age** | | | | | | | | | | |
| 20-29 **(**ref) | 1 | | 1 | | | 1 | | 1 | | |
| 30-39 | 1.50(0.75-3.02) | 0.25 | 1.10(0.93-1.30) | 0.38 | 0.10 | 2.24(1.12-4.49) | 0.02* | 1.13(0.94-1.30) | 0.25 | 0.39 |
| 40-49 | 1.10(0.93-1.33) | 0.30 | 1.08(0.90-1.30) | 0.36 | 0.24 | 1.42(0.65-3.11) | 0.38 | 1.10(0.91-1.33) | 0.34 | 0.17 |
| 50-59 | 1.18(0.92-1.52) | 0.18 | 1.11(0.84-1.47) | 0.84 | 0.10 | 3.61(0.99-13.00) | 0.05 | 1.18(0.92-1.52) | 0.18 | 0.78 |
| 60 and above | 1.10(0.83-1.46) | 0.49 | 1.02(0.77-1.35) | 0.88 | -0.07 | 0.65(0.22-1.94) | 0.44 | 1.10(0.83-1.46) | 0.49 | 0.49 |
| **Education** | | | | | | | | | | |
| No education (ref.) | 1 | | 1 | | | 1 | | 1 | | |
| Primary | 0.91(0.34-3.25) | 0.32 | 1.01(0.63-4.73) | 0.45 | 0.01 | 9.75(0.78-120.95) | 0.08 | 0.73(0.51-1.06) | 0.10 | 1.29 |
| Secondary | 0.30(0.11-1.82) | 0.51 | 0.46(0.21-2.34) | 0.32 | -0.43 | 4.10(0.44-38.42) | 0.22 | 0.83(0.68-1.00) | 0.06 | 0.79 |
| Tertiary | 0.28(0.17-2.13) | 0.21 | 0.33(0.18-3.00) | 0.12 | | 25.07(3.36-187.12 | 0.002* | 0.87(0.72-1.05) | 0.16 | 1.82 |
| **Smoke** | | | | | | | | | | |
| No **(ref.)** | 1 | | 1 | | | 1 | | 1 | | |
| Yes | 0.87(0.62-1.23) | 0.38 | 0.88(0.64-2.22) | 0.46 | -0.29 | 0.89(0.20-1.84) | 0.38 | 0.78(0.25-1.90) | 0.41 | -0.14 |
| **HOMA-B** | | | | | | | | | | |
| Normal (ref.) | 1 | | 1 | | | 1 | | 1 | | |
| High | 0.74(0.56-1.06) | 0.34 | 0.78(0.05-1.56) | 0.10 | -0.14 | 0.91(0.32-2.62) | 0.87 | 1.20(0.45-1.56) | 0.46 | 0.11 |
| Low | 0.56(0.4-1.24) | 0.64 | 0.06(0.03-1.10) | 0.13 | 0.11 | 0.12(0.04-1.35) | 0.56 | 0.56(0.06-1.11) | 0.34 | 0.01 |
| **WHR** | | | | | | | | | | |
| Normal **(ref.)** | 1 | | 1 | | | 1 | | 1 | | |
| High | 1.54(0.48-2.92) | 0.46 | 0.98(0.49-1.97) | 0.96 | -0.01 | 0.75(0.51-1.08) | 0.27 | 0.90(0.38-2.56) | 0.98 | -0.01 |
| **Waist circumference** | | | | | | | | | | |
| Normal (ref.) | 1 | | 1 | | | 1 | | 1 | | |
| High | 1.08(0.93-1.26) | 0.28 | 0.94(0.81-1.09) | 0.43 | 0.36 | 0.76(0.56-1.04) | 0.04* | 0.76(0.56-1.04) | 0.09 | -0.25 |
| **BMI** | | | | | | | | | | |

| | | | | | | | | | | |
|---|---|---|---|---|---|---|---|---|---|---|
| Normal **(ref.)** | 1 | | 1 | | | 1 | | 1 | | |
| Underweight | 0.94(0.53-1.68) | 0.84 | 0.92(0.54-1.57) | 0.77 | 0.38 | 0.89(0.56-1.39) | 0.61 | 0.91(0.58-1.44) | 0.71 | -0.23 |
| Overweight | 1.15(0.65-2.04) | 0.62 | 1.05(0.62-1.79) | 0.62 | 0.25 | 0.89(0.56-1.41) | 0.62 | 0.90(0.56-1.43) | 0.66 | -0.34 |
| Obese | 1.06(0.57-4.71) | 0.83 | 0.99(0.56-1.75) | 0.98 | 0.19 | 0.78(0.45-1.37) | 0.40 | 0.99(0.53-1.84) | 0.98 | -0.58 |
| **FPG** | | | | | | | | | | |
| Normal (ref) | 1 | | 1 | | | 1 | | 1 | | |
| High | 0.91(0.73-1.14) | 0.44 | 1.50(0.42-4.87) | 0.34 | 0.22 | 1.42(0.71-1.87) | 0.32 | 2.06(1.03-4.10) | 0.03* | 0.86 |
| **Insulin** | | | | | | | | | | |
| Normal (ref.) | **1** | | 1 | | | 1 | | 1 | | |
| High | 0.94(0.74-1.22) | 0.34 | 1.92(0.98-3.98) | 0.09 | 0.06 | 1.54(1.37-1.72) | <0.001* | 1.57(1.38-1.79) | <0.001* | 0.67 |
| **LDL** | | | | | | | | | | |
| Normal (ref.) | 1 | | 1 | | | 1 | | 1 | | |
| High | 0.94(0.80-1.10) | 0.48 | 1.08(0.87-2.32) | 0.17 | -0.02 | 1.15(0.98-1.35) | 0.08 | 1.23(0.98-1.55) | 0.06 | 0.14 |
| **HDL** | | | | | | | | | | |
| Normal | 1 | | 1 | | | | | | | |
| Low | 0.93(0.38-2.30) | 0.88 | 0.91(0.34-2.38) | 0.84 | -0.05 | 0.83(0.37-1.87) | 0.66 | 0.86(0.38-1.97) | 0.73 | -0.08 |
| **VLDL** | | | | | | | | | | |
| Normal (ref.) | 1 | | 1 | | | 1 | | 1 | | |
| High | 0.88(0.33-2.35) | 0.81 | 0.97(0.76-1.23) | 0.81 | 0.09 | 1.03(0.78-1.37) | 0.79 | 0.92(0.66-1.28) | 0.63 | 0.25 |
| **Cholesterol** | | | | | | | | | | |
| Normal **(ref.)** | 1 | | 1 | | | 1 | | 1 | | |
| High | 1.04(0.90-1.21) | 0.51 | 1.00(0.87-1.16) | 0.92 | 0.19 | 0.98(0.82-1.17) | 0.86 | 0.76(0.61-0.95) | 0.01* | -0.30 |
| **Triglyceride** | | | | | | | | | | |
| Normal **(ref.)** | **1** | | 1 | | | 1 | | 1 | | |
| High | 1.10(0.73-1.66) | 0.59 | 0.93(0.77-1.13) | 0.50 | 0.03 | 0.94(0.77-1.15) | 0.50 | 1.01(0.83-1.23) | 0.88 | 0.17 |
| **CRP** | | | | | | | | | | |
| Normal **(ref.)** | 1 | | 1 | | | 1 | | 1 | | |
| High | 1.08(0.94-1.25) | 0.21 | 1.18(0.90-1.54) | 0.21 | 0.25 | 0.98(0.83-1.15) | 0.85 | 0.96(0.82-1.12) | 0.65 | 0.04 |

**CRP:** C-reactive protein; **BMI:** Body mass index; **FPG:** Fasting plasma glucose; **HDL:** High-density lipoprotein cholesterol; **VLDL:** Very low-density lipoprotein cholesterol; **HOMA-IR:** Homeostatic model assessment of insulin resistance; **HOMA-B:** Homeostatic model of beta-cell function; **WHR:** Weight-to-height ratio; **COVID-19:** Coronavirus disease 2019; **CPR:** Crude prevalence ratio (univariate); **APR:** Adjusted prevalence ratio (multivariate); ***:** Significant p-value**; ES:** Effect size (Cohen's d)

***Predictors of insulin resistance measured by TyG according to past COVID-19 status***

In a sub-group analysis with TyG as the index for insulin resistance based on past COVID-19 status, age, BMI, triglycerides and CRP turned out as the statistically significant (P < 0.05; Table 6) predictors of insulin resistance in participants with past COVID-19 status. For participants without past COVID-19 status, age emerged as the sole predictor of insulin resistance in our setting (P < 0.05; Table 6). In general, the strength of the observed associations was small. Compared to age group 20-29 years , age 40-49 years demonstrated a 16% increased prevalence of insulin resistance (PR: 1.16; CI: 1.05-1.28; P = 0.003; Table 6) in participants with past COVID-19 status but in participants without past COVID-19 status, 11% increased prevalence of insulin resistance was observed (PR: 1.11; CI: 1.01-1.23; P = 0.03; Table 6) in age group 50-59 years. In the group with past COVID-19 status, participants with high triglycerides level compared with normal level showed a 9% increase in prevalence of insulin resistance (PR: 1.09; CI: 1.03-1.18; P = 0.01; Table 6). However, being underweight (PR: 0.86; CI: 0.77-0.95; P = 0.002; Table 6); overweight (PR: 0.88; CI: 0.78-0.99; P = 0.03; Table 6) or having high level of CRP (PR: 0.88; CI: 0.79-0.99; P = 0.03; Table 6), rather decreased the prevalence of insulin resistance compared with their counterparts with normal levels of these parameters in this group.

**Table 6: Predictors of insulin resistance measured by triglyceride-glucose index according to past COVID-19 status**

| | **PAST COVID-19 STATUS** | | | | | **NO PAST COVID-19 STATUS** | | | | |
|---|---|---|---|---|---|---|---|---|---|---|
| **Variable** | **CPR (95%CI)** | **P-value** | **APR (95%CI)** | **P – value** | **ES** | **CPR (95%CI)** | **P-value** | **APR (95%CI)** | **P-value** | **ES** |
| **Location** | | | | | | | | | | |
| Cape Coast **(**ref.) | 1 | | 1 | | | 1 | | 1 | | |
| Tamale | 0.93(0.87-1.00) | 0.1 | 0.93(0.85-1.00) | 0.06 | -0.79 | 0.64(0.23-1.76) | 0.49 | 1.00(0.89-1.13) | 0.97 | -0.06 |
| **Sex** | | | | | | | | | | |
| Females (ref.) | 1 | | 1 | | | 1 | | 1 | | |
| Males | 1.01(0.93-1.09) | 0.81 | 1.02(0.94-1.16) | 0.58 | 0.18 | 1.05(0.97-1.13) | 0.22 | 1.07(0.99-1.16) | 0.0.06 | 0.47 |
| **Age** | | | | | | | | | | |
| 20-29 **(ref)** | 1 | | 1 | | | 1 | | 1 | | |
| 30-39 | 1.07(0.97-1.18) | 0.21 | 1.04(0.95-1.16) | 0.38 | 0.39 | 1.05(00.96-1.16) | 0.30 | 1.03(0.94-1.14) | 0.54 | 0.06 |
| 40-49 | 1.15(1.06-1.24) | 0.001 | 1.16(1.05-1.28) | 0.003* | -0.03 | 0.99(0.87-1.14) | 0.94 | 0.98(0.86-1.11) | 0.73 | -0.39 |
| 50-59 | 1.06(0.90-1.26) | 0.46 | 1.09(0.93-1.29) | 0.27 | 0.38 | 1.13(1.05-1.21) | 0.001* | 1.11(1.01-1.23) | 0.03* | -0.01 |
| 60 and above | 0.91(0.70-1.20) | 0.53 | 0.91(0.70-1.19) | 0.49 | 0.21 | 1.05(0.90-1.23) | 0.50 | 0.98(0.85-1.13) | 0.82 | -0.03 |
| **Education** | | | | | | | | | | |

| | | | | | | | | | | |
|---|---|---|---|---|---|---|---|---|---|---|
| No education (ref.) | 1 | | 1 | | | 1 | | 1 | | |
| Primary | 0.95(0.78-4.18) | 0.15 | 0.98(0.70-4.00) | 0.11 | -0.01 | 1.02(0.84-1.23) | 0.87 | 1.03(0.85-1.25) | 0.76 | -0.08 |
| Secondary | 0.97(0.69-3.67) | 0.09 | 0.96(0.61-3.11) | 0.06 | -0.02 | 1.12(1.00-1.25) | 0.29 | 1.12(0.99-1.27) | 0.07 | 0.37 |
| Tertiary | 0.99(0.37-1.76) | 0.08 | 0.98(0.49-2.10) | 0.12 | -0.01 | 1.11(0.99-1.244) | 0.06 | 1.11(0.99-1.24) | 0.06 | 0.10 |
| **Smoke** | | | | | | | | | | |
| No **(ref.)** | 1 | | 1 | | | 1 | | 1 | | |
| Yes | 0.95(0.79-1.15) | 0.62 | 0.93(0.76-1.13) | 0.45 | -0.27 | 1.09(1.06-1.14) | <0.001* | 1.08(0.95-1.23) | 0.23 | |
| **HOMA-B** | | | | | | | | | | |
| Normal (ref.) | 1 | | 1 | | | 1 | | 1 | | |
| High | 0.98(0.88-2.13) | 0.12 | 0.87(0.80-1.59) | 0.18 | -0.48 | 0.22(0.10-1.69) | 0.12 | 0.34(0.21-1.20) | 0.20 | -0.86 |
| Low | 0.88(079-1.15) | 0.98 | 0.78(0.67-1.52) | 0.40 | | 0.58(0.20-1.73) | 0.33 | 0.51(0.15-1.64) | 0.28 | -0.39 |
| **WHR** | | | | | | | | | | |
| Normal **(ref.)** | 1 | | 1 | | | 1 | | 1 | | |
| High | 0.88(0.67-1.34) | 0.41 | 0.78(0.20-1.56) | 0.34 | -0.33 | 0.92(0.31-1.75) | 0.34 | 1.32(0.41-2.40) | 0.58 | 0.02 |
| **Waist circumference** | | | | | | | | | | |
| Normal (ref.) | 1 | | 1 | | | 1 | | | | |
| High | 0.99(0.90-1.09) | 0.89 | 0.90(0.78-1.03) | 0.12 | -0.65 | 1.08(0.99-1.16) | 0.05 | 0.96(0.86-1.07) | 0.47 | -0.37 |
| **BMI** | | | | | | | | | | |
| Normal (ref.) | 1 | | 1 | | | 1 | | 1 | | |
| Underweight | 0.87(0.83-0.94) | <0.001* | 0.86(0.77-0.95) | 0.002* | -0.03 | 1.09(0.70-1.70) | 0.69 | 1.15(0.73-1.79) | 0.55 | -0.63 |
| Overweight | 0.95(0.90-0.99) | 0.03* | 0.88(0.78-0.99) | 0.03* | 0.43 | 1.20(0.77-1.87) | 0.41 | 1.28(0.82-1.99) | 0.28 | 0.87 |
| Obese | 0.92(0.81-1.03) | 0.16 | 0.98(0.82-1.17) | 0.80 | 0.34 | 1.25(0.81-1.94) | 0.32 | 1.31(0.83-2.06) | 0.25 | -0.02 |
| **FPG** | | | | | | | | | | |
| Normal (ref.) | 1 | | 1 | | | 1 | | 1 | | |
| High | 1.33(0.76-2.35) | 0.32 | 1.31(0.83-2.05) | 0.24 | 0.75 | 1.19(0.55-2.62) | 0.46 | 1.17(0.57-2.34) | 0.67 | 1.11 |
| **Insulin** | | | | | | | | | | |
| Normal (ref.) | 1 | | 1 | | | 1 | | 1 | | |
| High | 1.26(0.71-2.22) | 0.42 | 1.22(0.78-1.89) | 0.39 | 0.79 | 1.02(0.93-1.11) | 0.72 | 1.00(0.91-1.09) | 0.99 | -0.03 |
| **LDL** | | | | | | | | | | |
| Normal (ref.) | 1 | | 1 | | | 1 | | 1 | | |
| High | 1.02(0.94-1.10) | 0.65 | 0.92(0.85-1.02) | 0.13 | 0.24 | 1.04(0.96-1.12) | 0.39 | 0.95(0.88-1.05) | 0.32 | 0.40 |
| **HDL** | | | | | | | | | | |
| Normal (ref.) | 1 | | 1 | | | 1 | | 1 | | |
| Low | 0.78(0.21-2.82) | 0.70 | 0.37(0.09-1.54) | 0.17 | -0.55 | 0.70(0.19-2.56) | 0.59 | 0.83(0.22-3.20) | 0.79 | -0.10 |

| | | | | | | | | | | |
|---|---|---|---|---|---|---|---|---|---|---|
| **VLDL** | | | | | | | | | | |
| Normal (ref.) | 1 | | 1 | | | 1 | | 1 | | |
| High | 1.10(1.06-1.15) | <0.001* | 0.99(0.93-1.05) | 0.71 | 0.01 | 0.97(0.82-1.15) | 0.75 | 0.96(0.81-1.13) | 0.62 | -0.54 |
| **Cholesterol** | | | | | | | | | | |
| Normal (ref.) | 1 | | 1 | | | 1 | | 1 | | |
| High | 1.06(0.99-1.14) | 0.09 | 1.02(0.92-1.10) | 0.49 | 0.47 | 1.09(1.02-1.17) | 0.01* | 1.10(0.98-1.24) | 0.08 | 1.01 |
| **Triglyceride** | | | | | | | | | | |
| Normal (ref.) | 1 | | 1 | | | 1 | | 1 | | |
| High | 1.13(1.07-1.18) | <0.001* | 1.09(1.03-1.18) | 0.01* | -0.01 | 1.02(0.93-1.11) | 0.69 | 1.00(0.91-1.10) | 0.63 | -0.01 |
| **CRP** | | | | | | | | | | |
| Normal (ref.) | 1 | | 1 | | | 1 | | 1 | | |
| High | 1.05(1.01-1.11) | 0.03* | 0.88(0.79-0.99) | 0.03* | -0.43 | 0.94(0.87-1.02) | 0.17 | 0.95(0.88-1.03) | 0.28 | -0.42 |

**CRP:** C-reactive protein; **BMI:** Body mass index; **FPG:** Fasting plasma glucose; **HDL:** High-density lipoprotein cholesterol; **VLDL:** Very low-density lipoprotein cholesterol; **HOMA-IR:** Homeostatic model assessment of insulin resistance; **HOMA-B:** Homeostatic model of beta-cell function; **WHR:** Weight-to-height ratio; **COVID-19:** Coronavirus disease 2019; **CPR:** Crude prevalence ratio (univariate); **APR:** Adjusted prevalence ratio (multivariate); ***:** Significant p-value**; ES:** Effect size (Cohen's d)

## DISCUSSION

Results from our analyses show that irrespective of the surrogate marker of insulin resistance or past COVID-19 status, age, educational level and triglycerides could significantly associate with insulin resistance.

In participants without past COVID-19 status, fasting plasma glucose, insulin and total cholesterol could significantly associate with insulin resistance measured by HOMA-IR. In these participants, high levels of plasma glucose and insulin exhibited 106% and 57% respective increased prevalence of insulin resistance compared with their counterparts with normal levels of the parameters. However, high total cholesterol level showed a 24% reduction in prevalence of insulin resistance. Irrespective of past COVID-19 status and residential location, 74.31% prevalence of insulin resistance was observed with HOMA-IR as the surrogate marker.

Using TyG as the index for insulin resistance, age, BMI, triglycerides and CRP were the significant predictors of insulin resistance in participants with past COVID-19 status. In these participants, being in age 40-49 years compared to age 20-29 years and having high triglycerides level compared to the normal level increased the prevalence of insulin resistance. However, being underweight or overweight compared to normal weight and having high level of CRP compared to the normal level decreased the prevalence of insulin resistance. For participants without past COVID-19

status, age group 50-59 years compared with 20-29 years increased the prevalence of insulin resistance as measured by TyG. Irrespective of past COVID-19 status and residential location, TyG-determined insulin resistance prevalence was 89.61%.

Insulin resistance remains a cardinal risk factor for the development of T2DM. The gold standard for the assessment of insulin resistance is the glucose clamp technique developed by DeFronzo et al. [17]. However, the method is expensive, laborious and requires high level of technical expertise, making it unsuitable for application in large-sample studies and routine clinical practice. Therefore, various less expensive and simple techniques that rely on surrogate markers have been developed for effective assessment of insulin resistance. The homeostatic model developed by Matthews et al. [14] to assess insulin resistance (HOMA-IR) and beta-cell function (HOMAB) and the triglyceride-glucose index (TyG) [15] are some of such methods that have been validated against the gold standard and found to be appropriate for assessing insulin resistance under fasting steady state [18, 19]. Insulin resistance is affected by race and other environmental factors, which differ from place to place. In the current study, we sought to examine determinants of insulin resistance, assessed by two surrogate markers, in non-diabetic participants with past COVID-19 status compared with their counterparts without any past COVID-19 status in two regional capitals of Ghana. The insulin resistance prevalence of 74.31% and 89.61% as determined by HOMA-IR and TyG respectively in our sample are higher than 38% reported in Lebanon [20], 46.4% in Venezuela [21], and 60.2% in Bangladesh [22]. This extremely high prevalence of insulin resistance exemplifies the risk of development of T2DM in our setting under favourable conditions. Considering that insulin resistance cuts across the various weight groups in our setting, with majority (52.25%) of participants within the normal weight category, other factors beyond being overweight or obese could be accounting for our observation. Indeed, irrespective of the surrogate marker used or past COVID-19 status, the prevalence of insulin resistance could not be predicted by body mass index, waist circumference, waist-to-hip ratio, C-reactive protein, smoking and alcohol intake in our setting although high level of circulating triglycerides increased the observed prevalence. Our findings appear contrary to those of previous studies [23-29] that associated indices of obesity and inflammation with insulin resistance. However, the observed positive association of insulin resistance with triglycerides level irrespective of past COVID-19 status or surrogate marker used in the current study is in support of studies [25, 29] that associated insulin resistance with lipid metabolism in furtherance of the established diabetogenic role of adiposity [30]. Indeed, white adipose tissue has long been implicated in the development of insulin resistance through production of adipocytokines and other lipid derivatives that impair insulin signaling [31-33].

A recent large-sample comprehensive epidemiological study which included 5340 participants and quantified 78 metabolic measures reported that non-diabetic individuals with insulin resistance are exposed to similar adverse metabolic environment in non-fasting state as their diabetic counterparts [34]. This observation implies that the metabolic response of the insulin resistant individual in the fed state may be even more important to the overall health than that of the fasting state used in various diagnostic decisions. Molecular and cellular evidence of IR show that the phenomenon results from altered phosphorylation in the insulin-signaling pathway in different cells and organs with overall adverse effects on carbohydrate, protein and lipid metabolism and associated health risk [35, 36]. Therefore, the failure of the above indices of obesity and inflammation to predict insulin resistance assessed under fasting state in our setting irrespective of past COVID-19 status or surrogate marker of insulin resistance does not imply that those biochemical processes are not involved at the molecular level with respect to the development of the condition. The difference in findings between the current study and the previous ones [23-29] could be ascribed to variations in participants characteristics. Indeed, none of these studies [23-29] involved individuals who had recovered from COVID-19. Indeed, sub-group analyses based on past COVID-19 status revealed a seeming protection against insulin resistance prevalence in both underweight and overweight participants with past COVID-19 status only. This finding portrays the complexity of the role of adiposity in the development of insulin resistance in our context.

However, the observation of insulin resistance across the various weight groups corroborates a number of previous studies [20-27] and implies that the development of insulin resistance probably precedes weight gain. Indeed, in spite of variations in mean or median levels of some of the measured biochemical indices between the two study groups, the observed levels were generally within the normal range, further supporting the notion that the development of insulin resistance does not necessarily require high levels of such indices. High levels of such indices may rather worsen an already existing condition. For instance, low-grade inflammation, is known to be associated with insulin resistance [37, 38] implying that a high grade version may aggravate the already established condition. Interestingly, the seeming promotion of insulin resistance by high levels of C-reactive protein, which could not be sustained when TyG was the surrogate marker, in the entire sample, irrespective of COVID-19 status, was rather sustained only in the sub-group analysis in participants with past COVID-19 status. This observation, together with the various predictors of insulin resistance identified by the two surrogate markers points to the complexity of the phenomenon and its associated factors.

Interestingly, a seeming protection from HOMA-IR-determined insulin resistance by tertiary education in the entire participants irrespective of past COVID-19 status changed to facilitation with TyG index as the surrogate marker although age 50-59 years was identified as a facilitator of insulin resistance by both surrogate markers in our setting. Moreover, high insulin level could only be found to be an important promoter of HOMA-IR-determined insulin resistance in participants without prior COVID-19 status in our context. Our observations in respect of the association of insulin resistance with high insulin level and advanced age appear to support previous studies [39, 40]. High insulin level can be a consequence of insulin resistance but it can also be an independent primary defect indicative of an unfavourable metabolic health [41, 42]. High insulin level is influenced by several factors including age, race, genetics, adiposity, free fatty acid levels, insulin secretion and clearance and it is self-perpetuating [41, 43]. A cell-based study [44] has long demonstrated that chronic exposure of insulin receptors to high levels of insulin impairs insulin signaling with time through altered autophosphorylation without affecting the quantity of receptors. This finding, which has been corroborated by other studies in humans [41, 43] as well as the current one, suggests that once hyperinsulinemic-insulin resistance cycle is established, metabolic health is adversarially affected if allowed to persist. Although a recent study by Stephens et al. [40] associated metabolic health with age and higher education, the nature of the reported association, notably, for education, differs from that of the current study in the context of TyG-determined insulin resistance. In the current study, tertiary education was positively associated with TyG-determined insulin resistance as opposed to the previous study [40] that reported a negative relationship. However, in terms of HOMA-IR-determined insulin resistance, our findings corroborate those of Stephens et al. [40]. As reported in previous studies [45, 46], the attainment of a tertiary education qualification is virtually a pre-requisite for decent employment notably in the formal sector of the Ghanaian economy. Moreover, with the retirement age being 60 years in Ghana, age 50-59 years corresponds with the age range at which participants will likely be at the peak of their career where various incentives that promote job-related sedentariness is prevalent. Such a situation can create a conducive metabolic environment for the development of insulin resistance as observed in the current study.

Interestingly, with TyG index as the surrogate marker, a sub-group analyses showed that in participants with past COVID-19 status, apart from age, formal education and BMI that associated with the observed insulin resistance, high triglycerides level promoted but high CRP level protected against the phenomenon. Whereas the protection offered

by high CRP level deviates from the expected facilitation role [37, 38], it further demonstrates the complexity of the relationship between CRP and the insulin resistance phenomenon.

Fasting plasma glucose (FPG) and beta-cell function (HOMA-B) are critical factors that are known to be associated with insulin resistance [47-49]. High FPG could be a consequence of insulin resistance or reduced beta-cell function or both although glucose-induced insulin resistance [47, 48] is possible, and can create a hyperglycaemia-insulin resistance cycle that requires an intervention to ensure a favourable metabolic health. Therefore, in the current study where insulin resistance is highly prevalent, participants with high fasting plasma glucose are expected to have a heightened risk of developing T2DM. High beta-cell function may demonstrate some protection against development of insulin resistance in the short-term but increases the diabetogenic risk in the long-term due to its probable unsustainable nature through exhaustion [49]. Irrespective of past COVID-19 status, neither FPG nor HOMA-B was associated with insulin resistance in our setting when TyG was used as the index for insulin resistance. However, with HOMA-IR as the surrogate marker, FPG in addition to insulin and total cholesterol levels associated with insulin resistance in participants without past COVID-19 status. This identification of different predictive variables of insulin resistance under different surrogate markers reflects the complexity of the phenomenon and its contributory factors [50].

Our study is not without limitations. Being a cross-sectional study, causal relationships could not be established. Also, detailed information on other lifestyle factors such as exercise and dietary information which can affect insulin resistance were not available. Above all, the use of fasting samples that are subject to fluctuations call for caution in undue extrapolation of our findings to populations of different characteristics. Therefore, longitudinal studies with wider coverage and larger sample size involving additional biomarkers that assess both fasting and postprandial insulin and glucose indices may be necessary to better understand the promoting factors of insulin resistance in our context. In spite of the above limitations, the current study has provided evidence of factors that affect the development of insulin resistance in participants with and without past COVID-19 status in our setting.

## CONCLUSION

The extremely high prevalence of insulin resistance in both groups of participants in addition to dyslipidemias and abnormal beta-cell function connotes increased diabetogenic risk. Irrespective of surrogate marker or past COVID-19

status, age, education and triglycerides levels associated with insulin resistance. In participants without prior COVID-19 status, FPG, insulin and total cholesterol associated with HOMA-IR-determined insulin resistance. However, with TyG index, age, BMI, triglycerides and CRP were the significant determinants of insulin resistance in participants with past COVID-19 status. The use of HOMA-IR and TyG as surrogate markers for insulin resistance allowed for the identification of a wide range of predictors of the condition in our context, which could not have been possible if only one surrogate marker had been used. Further studies are needed to unravel additional factors that influence the development of insulin resistance under fasting and postprandial states in apparently healthy adults in our setting even as health educational measures on lifestyle changes that improve insulin sensitivity are intensified.

**Authors' contribution**

**ASB, LDK, CA, JAA and SA** contributed to conception, design, analysis and interpretation, drafting of the manuscript and critical revision for important intellectual content. **EAB** contributed to data analysis and interpretation, drafting of the manuscript and critical revision for important intellectual content. **JAO and RKA** were involved in the design, data collection, analysis and interpretation. All authors read and approved the final manuscript for publication.

**Declaration of interests**

We declare that we have no known competing financial interests or personal relationships that could have appeared to influence the work reported in this paper.

**Acknowledgement**

The authors are very grateful to the study participants for their unique contribution in ensuring the success of this research work. Additionally, we are grateful to the staff and management of the Cape Coast and Tamale Teaching Hospitals for allowing their facilities to be used as sites for the study. Above are we are thankful to the staff and management of the Public Health Reference Laboratory of the Tamale Teaching Hospital for their support in the conduct of the study.

## REFERENCES

1. Ghana Health Service. COVID-19: Ghana's outbreak response and management updates [cited 15th February 2024]. Available from www.ghs.gov.gh/covid19/
2. International Diabetes Federation. IDF diabetes atlas. 10th ed. Brussels: International Diabetes Federation; 2021.

3. Acquah S. Implications of COVID-19 pandemic on evolution of diabetes in malaria-endemic African region. J Diabetes Res. 2020;2020:8205261. doi:10.1155/2020/8205261.
4. d'Annunzio G, Maffeis C, Cherubini V, Rabbone I, Scaramuzza A, Schiaffini R, et al. Caring for children and adolescents with type 1 diabetes mellitus: Italian Society for Pediatric Endocrinology and Diabetology (ISPED) statements during COVID-19 pandemia. Diabetes Res Clin Pract 2020;168:108372. doi:10.101/j.diabres.2020.108372.
5. Rabbone I, Schiaffini R, Cherubini V, Maffeis C, Scaramuzza A; Diabetes Study Group of the Italian Society for Pediatric Endocrinology and Diabetes.: has COVID-19 delayed the diagnosis and worsened the presentation of type 1 diabetes in children? Diabetes Care. 2020;43(11): 2870–2872. doi: 10.2337/dc20-1321.
6. Qeadan F, Tingey B, Egbert J, Pezzolesi MG, Burge MR, Peterson KA, et al. The associations between COVID-19 diagnosis, type 1 diabetes, and the risk of diabetic ketoacidosis: a nationwide cohort from the US using the Cerner Real-World Data. PLoS One. 2022;17(4):e0266809. doi: 10.1371/journal.pone.0266809.
7. Nassar M, Nso N, Baraka B, Alfishawy M, Mohamed M, Nyabera A, et al. The association between COVID-19 and type 1 diabetes mellitus: a systematic review. Diabetes Metab Syndr. 2021;15(1):447–454. doi: 10.1016/j.dsx.2021.02.009.
8. Mastromauro C, Blasetti A, Primavera M, Ceglie L, Mohn A, Chiarelli F, et al. Peculiar characteristics of new-onset type 1 diabetes during COVID-19 pandemic. Ital J Pediatr. 2022;48(1):26. doi: 10.1186/s13052-022-01223-8.
9. Chen M, Zhu B, Chen D, Hu X, Xu X, Shen WJ, et al. COVID-19 may increase the risk of insulin resistance in adult patients without diabetes: a 6-month prospective study. Endocr Pract. 2021;27(8): 834-841. doi: 10.1016/j.eprac.2021.04.004.
10. Santos A, Magro DO, Evangelista-Poderoso R, Saad MJA. Diabetes, obesity, and insulin resistance in COVID-19: molecular interrelationship and therapeutic implications. Diabetol Metab Syndr. 2021;13(1):23. doi: 10.1186/s13098-021-00639-2.
11. Cohen DH, LeRoith D. Obesity, type 2 diabetes, and cancer: the insulin and IGF connection. Endocr-Relat. Cancer 2012;19(5): F27–F45. doi: 10.1530/ERC-11-0374.
12. Kim HY. Statistical notes for clinical researchers: sample size calculation 1. comparison of two independent sample means. Restor Dent Endod. 2016;41:74-78. doi: 10.5395/rde.2016.41.2.154.
13. Adatsi RK., Bockarie AS, Derkyi-Kwarteng L, Pappoe F, Nsiah P, Dankwa K, et al. Increased insulin resistance with reduced beta cell function in recovered COVID-19 Ghanaians. Endocri Metab Sci. 2023;13: 100150. doi: 10.1016/j.endmts.2023.100150.
14. Matthews DR, Hosker JP, Rudenski AS, Naylor BA, Treacher DF, Turner RC. Homeostasis model assessment: insulin resistance and $\beta$-cell function from fasting plasma glucose and insulin concentrations in man. Diabetologia 1985; 28(7): 412–419. doi: 10.1007/BF00280883.
15. Simental-Mendía LE, Rodríguez-Morán M, Guerrero-Romero F. The product of fasting glucose and triglycerides as surrogate for identifying insulin resistance in apparently healthy subjects. Metab Syndr Relat Disord 2008;6(4): 299-304. doi: 10.1089/met.2008.0034.
16. Expert Panel on Detection, Evaluation, and Treatment of High Blood Cholesterol in Adults. Executive summary of the third report of the National Cholesterol Education Program (NCEP) Expert Panel on Detection, Evaluation, and Treatment of High Blood Cholesterol in Adults (Adult Treatment Panel III). JAMA. 2001;285(19): 2486-2497. doi: 10.1001/jama.285.19.2486.
17. DeFronzo RA, Tobin JD, Andres R. Glucose clamp technique: a method for quantifying insulin secretion and resistance. Am J Physiol. 1979;237(3): E214-223. doi: 10.1152/ajpendo.1979.237.3.E214.
18. Guerrero-Romero F, Simental-Mendía LE, González-Ortiz M, Martínez-Abundis E, Ramos-Zavala MG, Hernández-González SO, et al. The product of triglycerides and glucose, a simple measure of insulin sensitivity. Comparison with the euglycemic-hyperinsulinemic clamp. J Clin Endocrinol Metab. 2010;95(7):3347-3351. doi: 10.1210/jc.2010-0288.
19. Luo P, Cao Y, Li P, Li W, Song Z, Fu Z, et al. TyG index performs better than HOMA-IR in Chinese type 2 diabetes mellitus with a BMI < 35 kg/m$^2$: a hyperglycemic clamp validated study. Medicina (Kaunas). 2022;58(7):876. doi: 10.3390/medicina58070876.
20. Fahed M, Abou Jaoudeh MG, Merhi S, Mosleh JMB, Ghadieh R, Al Hayek S, et al. Evaluation of risk factors for insulin resistance: a cross sectional study among employees at a private university in Lebanon. BMC Endocr Disord. 2020;20(1):85. doi: 10.1186/s12902-020-00558-9.

21. Bermudez V, Salazar J, Martínez MS, Chávez-Castillo M, Olivar LC, Calvo MJ, et al. Prevalence and associated factors of insulin resistance in adults from Maracaibo City, Venezuela. Adv Prev Med. 2016;2016:9405105. doi: 10.1155/2016/9405105.
22. Sejooti SS, Naher S, Hoque MM, Zaman MS, Aminur Rashid HM. Frequency of insulin resistance in nondiabetic adult Bangladeshi individuals of different obesity phenotypes. Diabetes Metab Syndr. 2019;13(1):62-67. doi: 10.1016/j.dsx.2018.08.022.
23. Ozdemir O. Evaluation of waist circumference, waist-hip ratio and waist-length ratio in insulin resistance: which is a powerful predictor? Ann of Med Res 2023;30(5):533–537.
24. Cho SH, Jeong SH, Shin J, Park S, Jang SI. Short-term smoking increases the risk of insulin resistance. Sci Rep. 2022;12(1):3550. doi: 10.1038/s41598-022-07626-1.
25. Elrayess MA, Rizk NM, Fadel AS, Kerkadi A. Prevalence and predictors of insulin resistance in non-obese healthy young females in Qatar. Int J Environ Res Public Health. 2020;17(14):5088. doi: 10.3390/ijerph17145088.
26. Kim GR, Choi DW, Nam CM, Jang SI, Park EC. Synergistic association of high-sensitivity C-reactive protein and body mass index with insulin resistance in non-diabetic adults. Sci Rep. 2020;10(1):18417. doi: 10.1038/s41598-020-75390-1.
27. Jamar G, Almeida FR, Gagliardi A, Sobral MR, Ping CT, Sperandio E, et al. Evaluation of waist-to-height ratio as a predictor of insulin resistance in non-diabetic obese individuals. A cross-sectional study. Sao Paulo Med J. 2017;135(5): 462-468. doi: 10.1590/1516-3180.2016.0358280417.
28. Oumer A, Ale A, Tariku Z, Hamza A, Abera L, Seifu A. Waist-to-hip circumference and waist-to-height ratio could strongly predict glycemic control than body mass index among adult patients with diabetes in Ethiopia: ROC analysis. PLoS One. 2022;17(11):e0273786. doi: 10.1371/journal.pone.0273786.
29. Chen W, Ding S, Tu J, Xiao G, Chen K, Zhang Y, Huang R, Liao Y. Association between the insulin resistance marker TyG index and subsequent adverse long-term cardiovascular events in young and middle-aged US adults based on obesity status. Lipids Health Dis. 2023 May 18;22(1):65. doi: 10.1186/s12944-023-01834-y.
30. Gastaldelli A, Gaggini M, DeFronzo RA. Role of adipose tissue insulin resistance in the natural history of type 2 diabetes: Results from the San Antonio Metabolism Study. Diabetes. 2017;66(4): 815-822. doi: 10.2337/db16-1167.
31. McArdle MA, Finucane OM, Connaughton RM, McMorrow AM, Roche HM. Mechanisms of obesity-induced inflammation and insulin resistance: insights into the emerging role of nutritional strategies. Front Endocrinol (Lausanne). 2013 ;4:52. doi: 10.3389/fendo.2013.00052.
32. Sethi JK, Vidal-Puig AJ. Thematic review series: adipocyte biology. Adipose tissue function and plasticity orchestrate nutritional adaptation. J Lipid Res. 2007;48(6): 1253-1262. doi: 10.1194/jlr.R700005-JLR200.
33. Gual P, Le Marchand-Brustel Y, Tanti JF. Positive and negative regulation of insulin signaling through IRS-1 phosphorylation. Biochimie. 2005;87(1):99-109. doi: 10.1016/j.biochi.2004.10.019.
34. Wang Q, Jokelainen J, Auvinen J, Puukka K, Keinanen-Kiukaanniemi S, Jarvelin MR, et al. Insulin resistance and systemic metabolic changes in oral glucose tolerance test in 5340 individuals: an interventional study. BMC Med 2019;17(1):217. doi: 10.1186/s12916-019-1440-4.
35. da Silva Rosa S, Nayak N, Caymo AM, Gordon JW. Mechanisms of muscle insulin resistance and the cross-talk with liver and adipose tissue. Physiol Rep. 2020;8:e14607. doi: 10.14814/phy2.14607.
36. Lee SH, Park SY, Choi CS. Insulin resistance: from mechanisms to therapeutic strategies. Diabetes Metab J. 2022;46(1):15-37. doi: 10.4093/dmj.2021.0280.
37. Kawai T., Autieri M.V., Scalia R. Adipose tissue inflammation and metabolic dysfunction in obesity. Am J Physiol Cell Physiol. 2021;320:C375–C391. doi: 10.1152/ajpcell.00379.2020.
38. Püschel GP, Klauder J, Henkel J. Macrophages, low-grade inflammation, insulin resistance and hyperinsulinemia: a mutual ambiguous relationship in the development of metabolic diseases. J Clin Med. 2022;11(15):4358. doi: 10.3390/jcm11154358.
39. Vaidya RA, Desai S, Moitra P, Salis S, Agashe S, Battalwar R, et al. Hyperinsulinemia: an early biomarker of metabolic dysfunction. Front Clin Diabetes Healthc. 2023;4:1159664. doi: 10.3389/fcdhc.2023.1159664
40. Stephens CR, Easton JF, Robles-Cabrera A, Fossion R, de la Cruz L, Martínez-Tapia R, et al. The impact of education and age on metabolic disorders. Front Public Health. 2020;8:180. doi: 10.3389/fpubh.2020.00180.
41. Thomas DD, Corkey BE, Istfan NW, Apovian CM. Hyperinsulinemia: an early indicator of metabolic dysfunction. J Endocr Soc. 2019;3(9): 1727-1747. doi: 10.1210/js.2019-00065.

42. Tricò D, Natali A, Arslanian S, Mari A, Ferrannini E. Identification, pathophysiology, and clinical implications of primary insulin hypersecretion in nondiabetic adults and adolescents. JCI Insight. 2018;3(24):e124912. doi: 10.1172/jci.insight.124912.
43. Fryk E, Olausson J, Mossberg K, Strindberg L, Schmelz M, Brogren H, et al. Hyperinsulinemia and insulin resistance in the obese may develop as part of a homeostatic response to elevated free fatty acids: A mechanistic case-control and a population-based cohort study. EBioMedicine. 2021;65:103264. doi: 10.1016/j.ebiom.2021.103264.
44. Catalano KJ, Maddux BA, Szary J, Youngren JF, Goldfine ID, Schaufele F. Insulin resistance induced by hyperinsulinemia coincides with a persistent alteration at the insulin receptor tyrosine kinase domain. PLoS One. 2014;9(9):e108693. doi: 10.1371/journal.pone.0108693.
45. Quadlin N, VanHeuvelen T, Ahearn CE. Higher education and high-wage gender inequality. Soc Sci Res. 2023 May;112:102873. doi: 10.1016/j.ssresearch.2023.102873.
46. Ali MS, Jalal H. Higher Education as a Predictor of Employment: The World of Work Perspective. Bull Educ Res. 2018; 40(2): 79-90.
47. Zhang W, Liu J, Tian L, Liu Q, Fu Y, Garvey WT. TRIB3 mediates glucose-induced insulin resistance via a mechanism that requires the hexosamine biosynthetic pathway. Diabetes. 2013;62(12): 4192–4200. doi: 10.2337/db13-0312.
48. Kawanaka K, Han D-H, Gao J, Nolte LA, Holloszy JO. Development of glucose-induced insulin resistance in muscle requires protein synthesis. J Biol Chem. 2001;276(23): 20101–20107. doi: 10.1074/jbc.M010599200.
49. Esser N, Utzschneider KM, Kahn SE. Early beta cell dysfunction vs insulin hypersecretion as the primary event in the pathogenesis of dysglycaemia. Diabetologia. 2020;63(10):2007-2021. doi: 10.1007/s00125-020-05245-x.
50. Detopoulou P, Demopoulos CA, Antonopoulou S. Micronutrients, phytochemicals and Mediterranean diet: A potential protective role against COVID-19 through modulation of PAF actions and metabolism. Nutrients. 2021; 13(2): 462. doi: 10.3390/nu13020462.

Figure Click here to access/download;Figure;Plos Pace Figures.docx

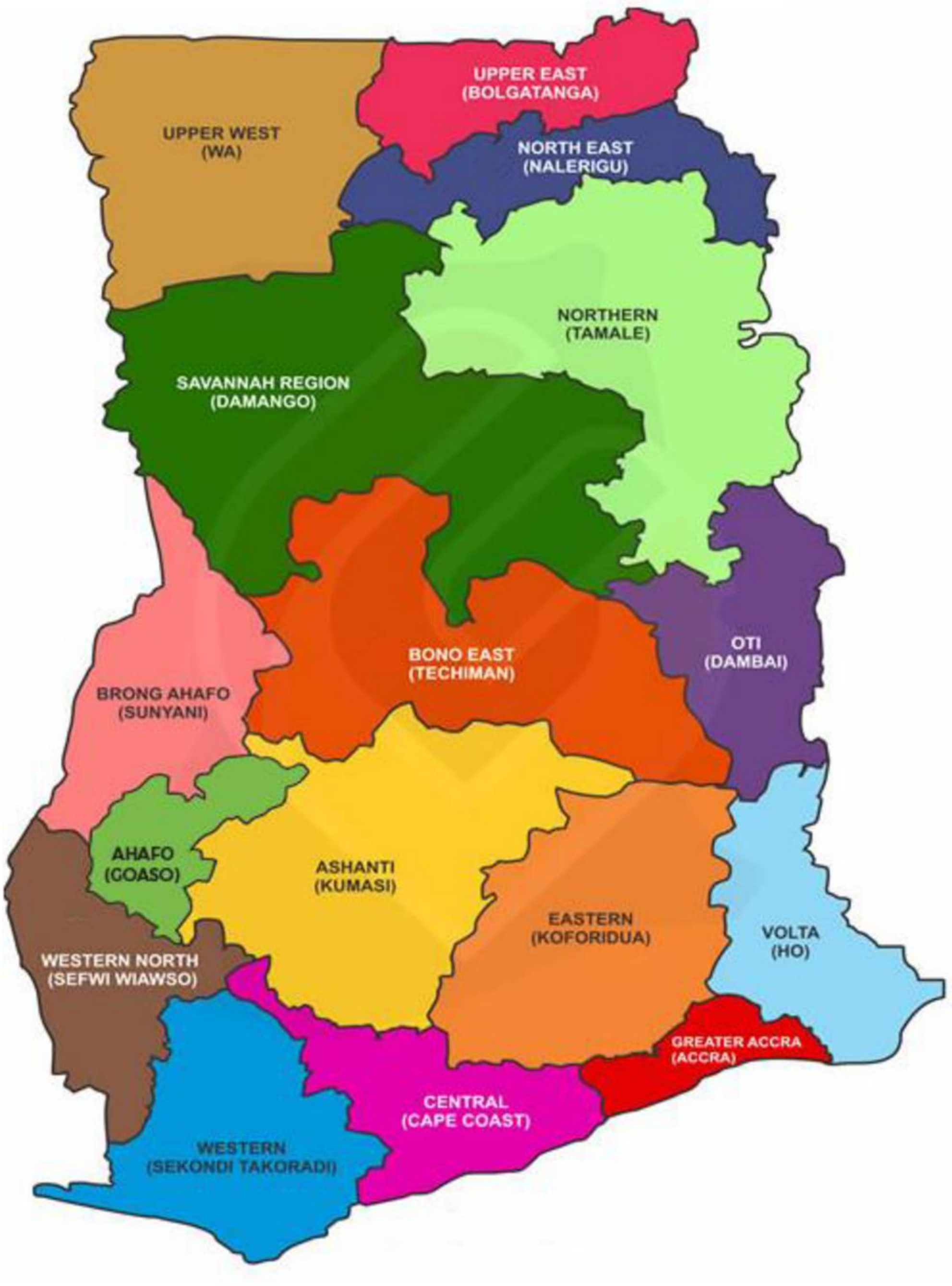


Fig. 1.

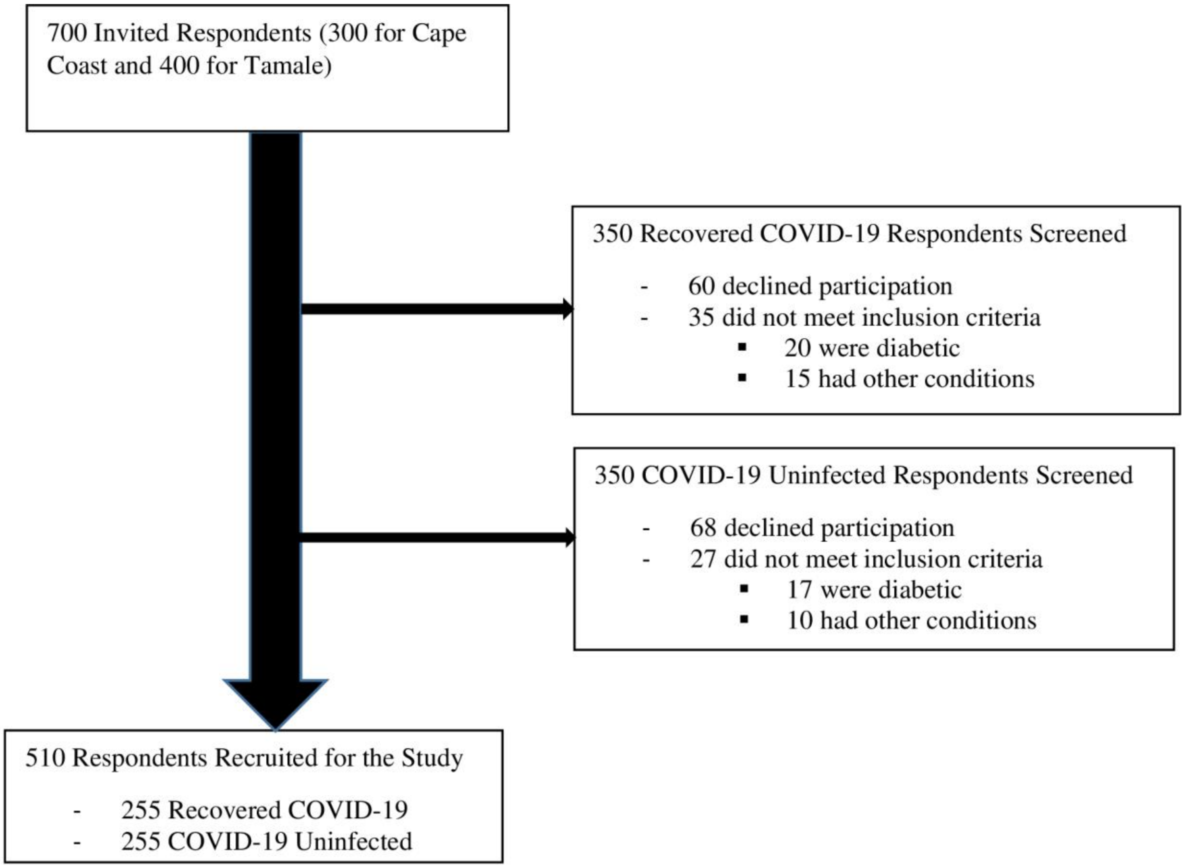
700 Invited Respondents (300 for Cape Coast and 400 for Tamale)
350 Recovered COVID-19 Respondents Screened
- 60 declined participation
- 35 did not meet inclusion criteria
▪ 20 were diabetic
▪ 15 had other conditions
350 COVID-19 Uninfected Respondents Screened
- 68 declined participation
- 27 did not meet inclusion criteria
▪ 17 were diabetic
▪ 10 had other conditions
510 Respondents Recruited for the Study
- 255 Recovered COVID-19
- 255 COVID-19 Uninfected

Fig. 2.

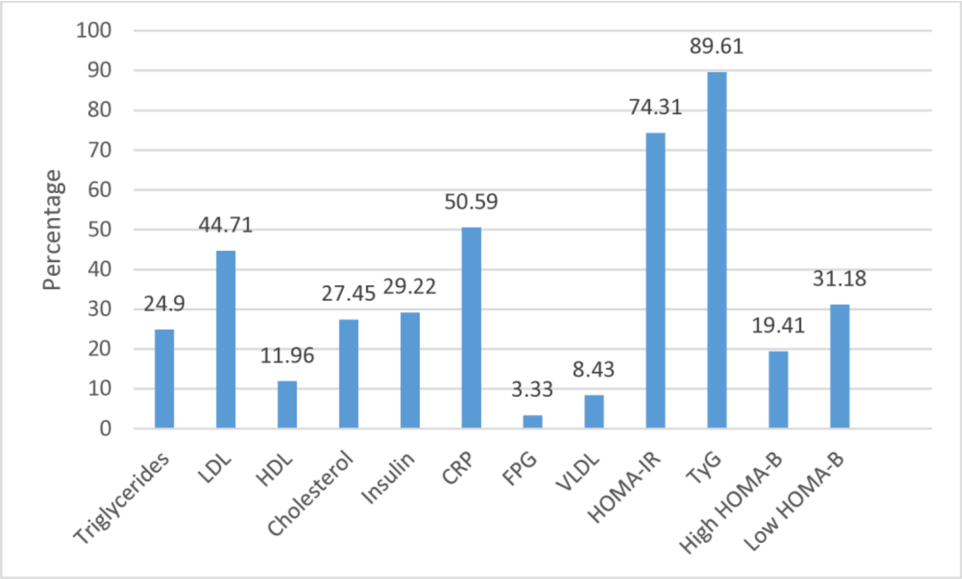
100
90
80
70
60
50
40
30
20
10
0
Percentage
24.9
44.71
11.96
27.45
29.22
50.59
3.33
8.43
74.31
89.61
19.41
31.18
Triglycerides
LDL
HDL
Cholesterol
Insulin
CRP
FPG
VLDL
HOMA-IR
TyG
High HOMA-B
Low HOMA-B

Fig. 3.

Click here to access/download
**Supporting Information**
Inclusivity-in-global-research-questionnaire.docx

**Prevalence and Determinants of Insulin Resistance in Recovered COVID-19 and Uninfected Residents of Two Regional Capitals in Ghana: An Observational Study**

**[1]Ansumana Sandy Bockarie, [2]Leonard Derkyi-Kwarteng, [3]Jeffrey Amankona Obeng, [4]Richard Kujo Adatsi, [5]Ebenezer Aniakwaa-Bonsu, [6]Charles Apprey, [7]Jerry Ampofo Asiamah, [8]Samuel Acquah**

1. Department of Internal Medicine and Therapeutics, School of Medical Sciences, College of Health and Allied Sciences, University of Cape Coast.
2. Department of Pathology, School of Medical Sciences, College of Health and Allied Sciences, University of Cape Coast.
3. Cape Coast Teaching Hospital, Cape Coast.
4. Public Health Reference Laboratory, Tamale Teaching Hospital, Tamale.
5. Department of Microbiology and Immunology, School of Medical Sciences, College of Health and Allied Sciences, University of Cape Coast.
6. Department of Biochemistry and Biotechnology, School of Biosciences, College of Science, Kwame Nkrumah University of Science and Technology, Kumasi, Ghana.
7. Department of Biochemistry, School of Biological Science, College of Agricultural and Natural Sciences, University of Cape Coast
8. Department of Medical Biochemistry, School of Medical Sciences, College of Health and Allied Sciences, University of Cape Coast.

***Corresponding author**, Samuel Acquah, Department of Medical Biochemistry, School of Medical Sciences, College of Health and Allied Sciences, University of Cape Coast.

Email: sacquah@ucc.edu.gh

**ABSTRACT**

**Background:** The long-term impact of the coronavirus disease 2019 (COVID-19) pandemic on metabolic risk factors in different populations has not been fully investigated. Insulin resistance (IR) is a cardinal risk factor for the development of type 2 diabetes mellitus. The current study sought to determine the prevalence and determinants of insulin resistance in selected Ghanaians with and without past COVID-19 status in the Cape Coast and Tamale metropolitan areas.

**Methods:** Using a cross-sectional study design involving 510 adult participants, body mass index (BMI), waist-to-hip ratio, systolic blood pressure, lipid profile, insulin, plasma glucose, C-reactive protein (CRP), beta-cell function and insulin resistance levels were measured and compared between participants with and without past COVID-19 status. Insulin resistance (IR) was determined by the homeostatic model (HOMA-IR) and the triglyceride-glucose index (TyG). Percentage prevalence and Poisson regression with prevalence ratio and 95% confidence intervals were applied.

**Results:** IR prevalence ranged from 70.69% to 79.09% (HOMA-IR) and 88.62% to 90.91% (TyG) respectively for Tamale and Cape Coast residents. IR prevalence values of 70.98% and 88% (HOMA-IR) and 89.02% and 90.2% (TyG) for uninfected and recovered COVID-19 groups respectively were observed. Irrespective of background, low (31.18%) and high (19.41%) levels of beta-cell function were detected. Additionally, high levels of very-low density lipoprotein cholesterol (8.31%), triglycerides (24.9%), total cholesterol (27.45%), low-density lipoprotein cholesterol (44.71%) and low level of high-density lipoprotein cholesterol (11.96%) coupled with low-grade inflammation (50.59 %) were observed. Irrespective of surrogate marker used or past COVID-19 status, age, educational level and triglycerides could significantly associate with IR. With HOMA-IR, fasting plasma glucose, insulin and total cholesterol predicted IR in participants without prior COVID-19 status. With TyG, age, BMI, triglycerides and CRP were the predictors of IR in participants with past COVID-19 status.

**Conclusion:** The risk of development of type 2 diabetes mellitus through insulin resistance is high in our setting. Measures to reduce the rising pace of IR are urgently needed in our setting.

**Key words:** COVID-19, Insulin resistance, beta-cell function, type 2 diabetes mellitus, diabetogenic risk.

## INTRODUCTION

Although the devastation of acute coronavirus disease 2019 (COVID-19) on various populations and world health systems appear to have abated, its long-term effects on the health and wellbeing of the affected is still persisting in various forms. Even in the sub-Saharan African region where the effect of the COVID-19 pandemic was not deemed as severe in terms of morbidity and mortality, the long-term impact on the populace cannot be underestimated. According to the latest data from the Ghana Health Service, the COVID-19 had affected 171,889 since its outbreak in 2020, as at March 2, 2023 with 70.8% of the population vaccinated against the condition [1].

Recent data from the International Diabetes Federation [2] still maintains that the sub-Saran African region has the highest prevalence of undiagnosed cases of diabetes and it is expected to experience the highest incidence of the condition by 2045. This gloomy prediction is hinged on predicted increased trend of obesity, driven by sedentary lifestyle and consumption of a westernized diet dominated by refined carbohydrates and saturated fats [2]. These diabetogenic predictions do not factor the probable role of infection-driven inflammation in the development of diabetes, especially, in the sub-Saharan African region. Indeed, the sub-Saharan African region continues to remain the hub of global infectious diseases [3]. It was probably based on this notion that the region was erroneously predicted to suffer the highest prevalence of mortality associated with acute COVID-19 infection in the early stages of the pandemic. In spite of the relatively mild short-term impact of COVID-19, in terms of morbidity and mortality on the regional populace, the probable long-term effects on the development of chronic non-communicable diseases in the region, especially, type 2 diabetes mellitus (T2DM), remains to be fully ascertained.

Interestingly, some studies have found that COVID-19 in people with diabetes increases diabetic complications and adverse outcomes of the viral condition [4-6]. Other studies [6-8] have associated the COVID-19 condition with new-onset diabetes of type 1 phenotype. Although T2DM constitutes more than 90% of global cases of diabetes, studies on the impact of COVID-19 on the condition post-recovery are limited.

Chen et al. [9] observed that six months after recovery from COVID-19, the risk of insulin resistance could increase. COVID-19-associated insulin resistance is thought to develop at several points of viral-host interactions and involves the integrated stress response (ISR) system [10]. The ISR includes a series of signaling pathways that are activated in response to various factors including obesity and viral infection and can result in impaired insulin action. Another important biochemical phenomenon that is central to the development of COVID-19-associated insulin resistance is

inflammation. Inflammation is a defense mechanism but can be harmful if dysregulated. Inflammation is linked to various health conditions including insulin resistance, diabetes, obesity, cancer and other chronic disease conditions [10, 11]. Interestingly, these conditions can also reinforce the inflammatory phenomenon.

Considering the critical role of insulin resistance in the pathogenesis of several chronic diseases in general and T2DM in particular, it is important to examine the promoting factors in our setting in order to identify appropriate mitigating measures to moderate its impact. This is critical because, insulin resistance, which is a cardinal risk factor for development of T2DM, can be influenced by several factors that may vary with the setting, making it difficult to adopt universal mitigating measures without recourse to the specific local context. Therefore, the current study aims to determine the prevalence and determinants of insulin resistance using two surrogate markers in a Ghanaian population that had recovered completely from COVID-19 and their counterparts that never had the condition in the Cape Coast and Tamale metropolitan areas.

## METHODS

### Study site, design and population

The study was conducted among residents of Cape Coast and Tamale metropolitan areas with the laboratory measurements taking place at the teaching hospitals of the two cities. The Cape Coast Teaching Hospital (CCTH) and the Tamale Teaching Hospital (TTH) are located in the Cape Coast and Tamale metropolis respectively. Both are tertiary facilities in the Ghanaian health care system and played a critical role in the management of COVID-19 cases during the pandemic. Whereas the CCTH is situated in the Central region along the coast of the Gulf of Guinea, TTH is located in Northern region of Ghana just below the Sahel belt. The two sites differ in geography, climate, ethnicity and dietary norms. The target population was the apparently healthy adults in the two metropolis. This cross-sectional study involved a total of 510 participants with 220 from Cape Coast and 290 from Tamale. The inclusion criteria were adults aged 20 years and above, apparently healthy, vaccinated against COVID-19 and without any underlying health condition at the time of recruitment. The participants who had recovered from COVID-19 had been declared healed from the condition by a negative PCR test results coupled with a clinical determination at least eight months before recruitment and were residents of either Tamale or Cape Coast and their surrounding communities. Those without a

history of the COVID-19 condition originated from the same communities as their recovered counterparts but were never diagnosed of the condition. Individuals who were pregnant, breastfeeding, diabetic, or had any health condition that could affect the levels of any of the blood-based measured indices were excluded from the study. The assessment of the inclusion and exclusion criteria was by effective administration of the appropriate questionnaires by trained health professionals. In addition, available health records of recovered COVID-19 were cross-checked with the responses of participants where appropriate. In both towns, workers in the formal sector of the Ghanaian economy dominated the participants. All participants had been vaccinated against the COVID-19 virus. Figure 1 shows a map of Ghana (https://www.ghanamissionun.org/wp-content/uploads/2023/11/Ghana_Regional_Map.jpg) with its administrative regional capitals including Cape Coast and Tamale where the study was conducted.

**Figure 1: Map of Ghana with its administrative capitals including the Cape Coast and Tamale metropolis**

***Determination of sample size***

The sample size was determined for each study site from the prevalence of COVID-19 at the national and regional levels by assuming the regional prevalence to be specific for the health facility of interest. Therefore, the regional prevalence of COVID-19 for the Central was assumed to be for the CCTH with that of the Northern region for the TTH, due to the critical roles that these tertiary health facilities played in the management of COVID-19 cases in their respective regions. The formula, $N = \{P1(1 - P1) + P2(1 - P2)\}\left(\frac{Z\alpha + Z\beta}{E}\right)^2$, [12] was applied to determine the sample size for each of the study sites. The estimated regional prevalence (P1) of COVID-19 were 3% and 0.15% for the Central and Northern regions respectively coupled with a national prevalence (P2) of 0.5%. However, the Central regional prevalence of the condition was applied across board in estimating the sample size in order to get adequate numbers for the study. Using the 3% regional and 0.5% national prevalence of COVID-19, coupled with a $Z_\alpha$ of 1.96

for a two-tailed test, $Z_\beta$ at 0.84 for 80% power and a margin of error (E) of 5%, the sample size was estimated to be 106 for each study group giving rise to 212 participants for the two groups in each study site. This implies that for both CCTH and TTH, a total of 424 participants would have been adequate for the study. Ultimately, in the current analysis, data from 510 participants made up of 220 from Cape Coast and 290 from Tamale were used.

***Selection of study participants***

The simple random sampling technique was used to recruit 255 participants with past COVID-19 status and an equal without past COVID-19 status, for the study. Figure 2 outlines the selection of study participants at the two study sites. Participants were recruited between 1st July, 2022 and 30th June 2023 for the CCTH site. However, for the TTH arm, recruitment started from 1st March 2023 to 30th November, 2023. Since management of COVID-19 in Ghana was reserved for selected teaching hospitals including the CCTH and TTH, records of such individuals could be assessed and contacted for the study. Therefore, 150 and 200 recovered COVID-19 participants were randomly selected from the CCTH and TTH respectively and contacted by telephone calls for participation in the study. Similarly, 150 and 200 control participants were selected from the Cape Coast and the Tamale metropolitan areas in a random manner in line with the study protocol for participation. After appropriate screening to ensure compliance with inclusion and exclusion criteria, 220 and 290 participants from the Cape Coast and Temale metropolitan areas respectively were recruited for the study. Details of the recruitment are shown below in figure 2.

**Figure 2: Flow Chart for Participants' Selection**

**Assessment of Study Variables**

To achieve the objectives of the study, the following variables of demographic, anthropometric and biochemical relevance were assessed.

***Demographic variables***

Demographic information including age, education, past COVID-19 status, smoking and alcohol consumption status were collected by a structured questionnaire designed for the study.

***Blood pressure and anthropometric measurements***

An experienced nurse measured the blood pressure of the participant with an electronic sphygmomanometer (Contec Medical Systems Co. Ltd, China) in a routine manner. The blood pressure was measured on the right arm of participants in sitting position after a minimum of five minutes rest. An average of two measurements that did not differ by >5 mmHg was recorded for each participant as the blood pressure.

Anthropometric indices such as weight (kg), height (m), waist circumference (cm) and hip circumference (cm) were measured for computation of waist-to-hip ratio and body mass index (BMI). Weight was measured to the nearest 0.1 kg with height to the nearest 0.1 cm. BMI was calculated by dividing the weight in kilogramme by the square of the height in metre ($kg/m^2$). Weight and height were measured in light clothing without footwear. Weight was measured by an electronic scale and the height by stadiometer (Seca® mechanical column scale with stadiometer, USA). Waist and hip circumferences were measured in centimetres with an inflexible tape measure. Waist circumference was measured at the midpoint between the lower margin of the last rib and the top of the iliac crest but the hip circumference was measured around the widest portion of the buttocks. Waist-to-hip ratio was obtained by dividing the waist circumference by the hip circumference.

***Measurement of glucose, lipid profile, insulin and C-reactive protein levels***

Measurements of plasma glucose and lipid profile including LDL, HDL, total cholesterol, triglyceride and VLDL were done with the Mindray BS240 automated chemistry analyser (Mindray Diagnostics, Nanshan Shenzhen, China) following a routine procedure.

However, serum insulin and C-reactive protein (CRP) levels were determined by immunoturbidimetric test kits purchased from Kamiya Biomedicals Company (K-ASSAY Seattle, USA) in accordance with the manufacturer's detailed instructions outlined by Adatsi et al. [13].

***Assessment of insulin resistance and beta-cell function***

Insulin resistance was assessed by the homeostatic model, $HOMA - IR = Glucose\ \left(\frac{mmol}{l}\right) * Insulin\ (m\frac{U}{l})/22.5$ [14] and the triglyceride-glucose (TyG) index, $TyG = In\left[FBS\left(\frac{mg}{dl}\right) * TG\left(\frac{mg}{dl}\right)\right]/2$ [15]. Additionally, beta-cell function was determined using the homeostatic model, $HOMAB = 20 * Fasting\ Insulin\ (\frac{mU}{l})/$ $Fasting\ Glucose\ (\frac{mmol}{l}) - 3.5$ developed by Matthews et al. [14].

**Determination of abnormal levels of blood-based parameters**

Dyslipidemias was assessed in accordance with the National Cholesterol Education Program Adult Treatment Panel (NCEP-ATP) III guidelines [16]. Specifically, abnormal levels of the lipid indices were defined as triglycerides ≥ 1.7 mmol/l; low-density lipoprotein cholesterol (LDL) ≥ 2.6 mmol/l; high-density lipoprotein cholesterol (HDL) < 1.03 mmol/l; total cholesterol ≥ 5.2 mmol/l. In addition, high levels of the other indices were defined as glucose > 6.4 mmol/l; insulin > 24.9 mIU/l; insulin resistance, HOMA-IR > 2.6 or TyG index > 4.0; beta-cell function, HOMA-B, high > 100 % or low < 25 %.

**Ethical Approval**

The Ethical Review Committee of the Tamale Teaching Hospital (TTHERC/24/02/23/01) granted approval for the study at Tamale as Cape Coast Teaching Hospital (CCTHERC/EC/2022/111) approved that for the Cape Coast arm. A written informed consent was provided by each study participant and at all times, the study protocols adhered strictly to the ethical standards of the Ghana Health Service and the World Medical Association Declaration of Helsinki.

**Data analysis**

Statistical Software for Data Science (STATA) version 15 Corp (StataCorp LLC, USA) software was used to analyze the data for this study. Demographic characteristics and other categorical variables

were presented in frequencies and percentages and compared with the chis-square goodness of fit test. Continuous variables were presented as mean and standard deviation if normally distributed or median and interquartile ranges if distribution deviated from normal distribution. The independent sample t-test was used to compare mean levels of measured indices between participants with and without past COVID-19 status for continuous variables that met assumptions for parametric test else the Mann Whitney test was used. Poisson regression with prevalence ratio was used to examine the relationship between insulin resistance, measured by HOMAIR or TyG, as the dependent variable and the other predictor variables. The univariate followed by the multivariate regression analyses were applied. Before fitting the multivariate regression model, the variance inflation factor (VIF) and tolerance (1/VIF) were used to assess multicollinearity among the independent variables, and no evidence of collinearity was found. The results of the regression analyses were presented as crude prevalence ratios (CPRs) and adjusted prevalence ratios (APRs) with 95% confidence intervals (CIs) and corresponding p-values. The determination of statistical significance for all analyses was considered at $p < 0.05$. Effect size (Cohen's d) was computed for the adjusted models and the mean/median comparisons to examine the strengths of the relationships. The standard interpretations for effect size were adopted in interpreting our data.

## RESULTS

In the current study, two different surrogate markers (HOMA-IR and TyG) were used for the assessment of insulin resistance to allow for probable identification of variety of promoters of the condition in our context. Various demographic, anthropometric and blood-based indices known to be associated with insulin resistance in various populations were assessed in the current study that involved 510 residents of Tamale and Cape Coast metropolis.

Data from 290 participants from Tamale (150 females and 140 males) and 220 participants from Cape Coast (123 males and 97 females) were used in our analysis.

### *Sociodemographic factors*

The sociodemographic characteristics of participants (table 1) indicate that a significant ($P < 0.05$; Table 1) majority of the participants were residents of Tamale (56.86%), aged 20-39 years (69.02%), had tertiary education (65.29%),

non-smokers (95.88%) and did not drink alcohol (76.08%). In terms of gender, proportions of male and female were similar statistically (P = 0.48; Table 1).

**Table 1: Sociodemographic characteristics of participants**

| Parameter | Frequency (%) | P-value |
|---|---|---|
| **Location** | | 0.002* |
| Cape Coast | 220 (43.14) | |
| Tamale | 290 (56.86) | |
| **Gender** | | 0.48 |
| Female | 247 (48.43) | |
| Male | 263 (51.57) | |
| **Age** | | < 0.001* |
| 20-29 | 189 (37.06) | |
| 30-39 | 163 (31.96) | |
| 40-49 | 92 (18.04) | |
| 50-59 | 36 (7.06) | |
| 60 and above | 30 (5.88) | |
| **Education** | | < 0.001* |
| No education | 75 (14.71) | |
| Primary | 24 (4.71) | |
| Secondary | 81 (15.88) | |
| Tertiary | 330 (64.71) | |
| **Past COVID-19** | | 1.00 |
| No | 255 (50) | |
| Yes | 255 (50) | |
| **Smoking status** | | < 0.001* |
| No | 489 (95.88) | |
| Yes | 21 (4.12) | |
| **Alcohol consumption** | | < 0.001* |
| No | 388 (76.08) | |
| Yes | 122 (23.92) | |

**COVID-19:** Coronavirus disease 2019; ***:** Significant p-value for chi-square goodness of fit test

***Mean/median levels of measured indices***

Compared to the uninfected group, the recovered COVID-19 participants had higher (P < 0.05) levels of waist circumference, hip circumference, fasting plasma glucose, insulin, low-density lipoprotein cholesterol, total cholesterol, HOMA-IR and C-reactive protein (Table 2) although the observed differences were generally small.

**Table 2: Levels of measured parameters by COVID-19 status**

| Parameter | Entire participants Mean ± SD | COVID-19 uninfected Mean ± SD | Recovered COVID-19 Mean ± SD | P-value | Effect size (Cohen's d) |
|---|---|---|---|---|---|
| Weight (kg) | 71.73 ± 14.28 | 70.64 ± 14.60 | 72.83 ± 13.89 | 0.08 | 0.15 |
| Height (m) | 1.68 ± 0.09 | 1.68 ± 0.10 | 1.68 ± 0.08 | 0.87 | 0.01 |
| Waist circumference (cm) | 82.61 ± 11.67 | 81.46 ± 11.92 | 83.75 ± 11.33 | 0.03* | 0.20 |
| Hip circumference (cm) | 95.80 ± 13.06 | 94.31 ± 0.85 | 97.28 ± 0.79 | 0.01* | 0.23 |
| WHR | 0.86 ± 0.06 | 0.87 ± 0.06 | 0.86 ± 0.06 | 0.52 | 0.06 |
| Systolic BP (mmHg) | 126.33 ± 14.83 | 126.24 ± 15.47 | 126.41 ± 14.19 | 0.9 | 0.01 |
| BMI ($kg/m^2$) | 25.43 ± 5.07 | 25.12 ± 4.98 | 25.75 ± 5.14 | 0.16 | 0.15 |
| FPG (mmol/L) | 4.74 ± 0.95 | 4.53 ± 0.81 | 4.94 ± 1.04 | <0.001* | 0.44 |
| | **Median (IQR)** | **Median (IQR)** | **Median (IQR)** | | |
| Insulin (mIU/L) | 18.45 (13.4-27.5) | 17.80 (12.40-23.40) | 19.4 (14.1-45.6) | <0.001* | 0.17 |
| HOMA-B (%) | 115.93 (68.9-295.60) | 111.71 (66.09-253.04) | 127.19 (69.57-372.50) | 0.12 | 0.07 |
| HOMAIR (mIU/L) | 3.72 (2.57-5.99) | 3.43 (2.47-4.74) | 4.25 (2.78-10.63) | <0.001* | 0.20 |
| TyG index | 4.46 (4.26-4.71) | 4.45 (4.25-4.66) | 4.49 (4.33-4.73) | 0.26 | 0.05 |
| HDL (mmol/L) | 1.73 (1.32-2.45) | 1.70 (1.32-2.33) | 1.74 (1.29-2.59) | 0.39 | 0.04 |
| LDL (mmol/L) | 2.46 (1.62-3.48) | 2.30 (1.48-3.38) | 2.81 (1.78-3.54) | 0.03* | 0.10 |
| VLDL(mmol/L) | 0.43 (0.33-0.7) | 0.39 (0.33-0.66) | 0.46 (3.13-72.4) | 0.9 | 0.07 |
| Total cholesterol (mmol/L) | 4.42 (3.24-5.32) | 4.21 (3.02-5.32) | 4.56 (3.67-5.32) | 0.04* | 0.10 |
| Triglyceride (mmol/L) | 0.99 (0.71-1.62) | 1.01 (0.72-1.52) | 0.97 (0.71-1.69) | 0.74 | 0.02 |
| CRP (mg/dL) | 1.00 (0.4-1.4) | 0.80 (0.30-1.20) | 1.20 (0.70-1.78) | <0.001* | 0.26 |

**CRP:** C-reactive protein; **BMI:** Body mass index; **FPG:** Fasting plasma glucose; **HDL:** High-density lipoprotein cholesterol; **VLDL:** Very low-density lipoprotein cholesterol; **HOMA-IR:** Homeostatic model assessment of insulin resistance; **HOMAB:** Homeostatic model of beta-cell function; **BP:** Blood pressure; **WHR:** Weight-to-height ratio; **SD:** Standard deviation; **IQR:** Inter-quartile range; **COVID-19:** Coronavirus disease 2019; ***:** Significant p-value for independent sample t-test (mean) or Mann Whitney test (median).

***Abnormal levels of measured variables***

Varied proportions (Figure 3) of abnormal levels of the measured indices were observed among the study participants. For example, the prevalence of insulin resistance (IR) ranged from 70.69% (Tamale) to 79.09% (Cape Coast) for the HOMA-IR index with an overall rate of 74.31% (Table 3 and figure 3). With TyG index, the observed IR prevalence were 88.62% and 90.91% (Table 4) respectively for the Tamale and Cape Coast residents with an overall rate of 89.61% (figure 3). However, the respective IR prevalence of 70.98% and 88% (HOMA-IR; Table 3) and 89.02% and 90.2% (TyG; Table 4) for participants without past COVID-19 status and those with past COVID-19 status were observed. Irrespective of location or COVID-19 status, low (31.18%) and high (19.41%) levels of beta-cell function were also observed (figure 3). In terms of dyslipidemias, prevalence values of 8.43 % for very low-density lipoprotein

cholesterol, high levels of very-low density lipoprotein cholesterol (8.31%), triglycerides (24.9%), total cholesterol (27.45%), low-density lipoprotein cholesterol (44.71%) and low level of high-density lipoprotein cholesterol (11.96%) and low-grade inflammation (50.59 %) were noted (figure 3).

**Figure 3: Prevalence of abnormal levels of blood-based indices**

*Except otherwise indicated, all the proportions represent percentage of individuals with levels of measured indices above the upper threshold of the reference range apart from HDL which represents a level below the lower threshold of its reference range.* ***CRP:*** *C-reactive protein;* ***BMI:*** *Body mass index;* ***FPG:*** *Fasting plasma glucose;* ***HDL:*** *High-density lipoprotein cholesterol;* ***VLDL:*** *Very low-density lipoprotein cholesterol;* ***HOMA-IR:*** *Homeostatic model assessment of insulin resistance;* ***HOMAB:*** *Homeostatic model of beta-cell function;* ***LDL:*** *low-density lipoprotein cholesterol;* ***TyG:*** *triglyceride-glucose index*

***Predictors of insulin resistance determined by HOMA-IR***

In a regression analysis to identify predictors of insulin resistance using HOMA-IR as the dependent variable in the entire sample irrespective of past COVID-19 status, age, education and triglycerides came out as the only predictors of insulin resistance (Table 3). Residing in Tamale decreased the prevalence of insulin resistance by 11% (PR: 0.89; CI: 0.81-0.99; P = 0.03; Table 3) compared to being a resident of Cape Coast in a univariate analysis. However, this reduction could not be sustained after adjusting for confounders (PR: 0.96; CI: 0.91-1.02; P = 0.23; Table 3**)** in a multivariate analysis although 79.09% (174/220) of Cape Coast residents and 70.69% (205/290) of Tamale residents were insulin resistant. Compared with age 20-29 years, participants in age group 30-39 and 50-59 years had respective increased prevalence of 18% (PR: 1.18; CI: 1.03-1.33; P = 0.01; Table 3) and 27% (PR: 1.27; CI: 1.07-1.50; P = 0.004; Table 3) in having insulin resistance with no such effect observed for those in age group 40-49 or 60 years and above. After controlling for confounders, only the participants in age group 50-59 years demonstrated 11% increase in prevalence of insulin resistance (PR: 1.11; CI: 1.02-1.21; P = 0.01; Table 3). The strength of the observed associations ranged from small (30-39 years group) to medium (50-59 years group). Having high glucose level did not demonstrate statistically significant association with insulin resistance although high triglycerides increased the prevalence by 5% (PR: 1.05; CI: 1.00-1.11; P = 0.04; Table 3) with medium strength of the observed association. Interestingly, the prevalence of insulin resistance increased by 13% (PR: 1.13; CI: 1.01-1.27; P = 0.03; Table 3) in participants with secondary education but decreased by 98% (PR: 0.02; CI: 0.002-0.14; P < 0.001; Table 3) in those with tertiary education in our setting. Indeed, across levels, the strength of the association between insulin resistance and formal education in general, was large in our context. Statistically, neither participants with high insulin levels (PR: 1.18; CI: 0.75-1.85; P = 0.46; Table 3) nor past

COVID-19 status (PR: 0.98; CI: 0.92-1.04; P = 0.56; Table 3) demonstrated increased prevalence of insulin resistance compared to their counterparts with normal levels of the parameter or without past COVID-19 status in our context.

**Table 3: Predictors of insulin resistance determined by HOMA-IR**

| Parameter | No insulin resistance, N (%) | Insulin resistance, N (%) | CPR (95% CI) | P-value | APR (95%CI) | P-value | Effect size (Cohen's d) |
|---|---|---|---|---|---|---|---|
| **Location** | | | | | | | |
| Cape Coast (ref.) | 46 (35.11) | 174 (45.91) | 1 | | 1 | | |
| Tamale | 85 (64.89) | 205 (54.09) | 0.89(0.81-0.99) | 0.03* | 0.96(0.91-1.02) | 0.23 | -0.24 |
| Gender | | | | | | | |
| Female (ref.) | 57 (43.51) | 190 (50.13) | 1 | | 1 | | |
| Male | 74 (56.49) | 189 (49.87) | 0.93(0.84-1.03) | 0.19 | 1.04(0.99-1.10) | 0.11 | -0.18 |
| **Age** | | | | | | | |
| 20-29 (ref) | 61 (46.57) | 128 (33.77) | 1 | | 1 | | |
| 30-39 | 33 (25.19) | 130 (34.30) | 1.18(1.03-1.33) | 0.01* | 1.03(0.96-1.10) | 0.35 | 0.29 |
| 40-49 | 22 (16.79) | 70 (18.47) | 1.12(0.97-1.31) | 0.15 | 1.06(0.98-1.15) | 0.2 | 0.21 |
| 50-59 | 5 (3.82) | 31 (8.18) | 1.27(1.07-1.50) | 0.004* | 1.11(1.02-1.21) | 0.01* | 0.64 |
| 60 and above | 10 (7.63) | 20 (5.28) | 0.98(0.75-1.29) | 0.91 | 0.97(0.84-1.12) | 0.71 | -0.09 |
| **Education** | | | | | | | |
| No education (ref.) | 1 (0.76) | 75 (19.79) | 1 | | 1 | | |
| Primary | 3 (2.29) | 20 (5.28) | 0.73(0.51-1.06) | 0.11 | 1.03(0.85-1.26) | 0.73 | -1.08 |
| Secondary | 9 (6.87) | 72 (18.99) | 0.83(0.69-1.00) | 0.06 | 1.13(1.01-1.27) | 0.03* | -1.17 |
| Tertiary | 118 (90.08) | 212 (55.94) | 0.95(0.83-1.08) | 0.44 | 0.02(0.002-0.14) | <0.001* | -2.16 |
| **Smoke** | | | | | | | |
| No (ref.) | 126 (96.18) | 363 (95.78) | 1 | | 1 | | |
| Yes | 5 (3.82) | 16 (4.22) | 1.02(0.80-1.31) | 0.84 | 0.96(0.83-1.11) | 0.61 | 0.07 |
| **Past COVID-19** | | | | | | | |
| No (ref.) | 74 (56.49) | 181 (47.76) | 1 | | 1 | | |
| Yes | 57 (43.51) | 198 (52.24) | 1.09(0.98-1.21) | 0.09 | 0.98(0.92-1.04) | 0.56 | 0.23 |
| **HOMA-B** | | | | | | | |
| Normal (ref.) | 7 (5.34) | 245 (64.64) | 1 | | 1 | | |
| High | 38 (29.01) | 61 (16.09) | 1.45(0.76-1.56) | 0.39 | 1.08(0.18-1.29) | 0.15 | -0.39 |
| Low | 86 (65.65) | 73 (19.26) | 0.99(0.89-1.20) | 0.67 | 0.98(0.70-1.14) | 0.23 | -1.65 |

| | | | | | | | |
|---|---|---|---|---|---|---|---|
| **Waist-to-hip ratio** | | | | | | | |
| Normal (ref.) | 71 (54.20) | 219 (57.78) | 1 | | 1 | | |
| High | 60 (45.80) | 160 (42.22) | 0.91 (0.61-1.36) | 0.63 | 0.86(0.57-1.31) | 0.49 | -0.08 |
| **Waist circumference** | | | | | | | |
| Normal (ref.) | 108 (82.44) | 321 (84.70) | 1 | | 1 | | |
| High | 23 (17.56) | 58 (15.30) | 0.96(0.82-1.11) | 0.59 | 0.93(0.85-1.02) | 0.14 | -0.13 |
| **BMI** | | | | | | | |
| Normal (ref.) | 100 (76.34) | 210 (55.41) | 1 | | 1 | | |
| Underweight | 10 (7.63) | 15 (3.96) | 0.91(0.64-1.30) | 0.62 | 1.01(0.79-1.31) | 0.87 | 0.18 |
| Overweight | 16 (12.21) | 115 (30.34) | 1.02(0.71-1.46) | 0.90 | 1.09(0.85-1.40) | 0.47 | 0.22 |
| Obese | 5 (3.82) | 39 (10.29) | 0.94(0.63-1.39) | 0.74 | 1.16(0.90-1.51) | 0.23 | 0.06 |
| **FPG** | | | | | | | |
| Normal (ref.) | 129 (92.37) | 363 (94.72) | 1 | | 1 | | |
| High | 2 (1.53) | 16 (4.22) | 2.67 (1.18-6.03) | 0.02* | 1.32(0.90-1.94) | 0.15 | 0.68 |
| **Insulin** | | | | | | | |
| Normal (ref.) | 130 (99.24) | 231 (60.95) | 1 | | 1 | | |
| High | 1 (0.76) | 148 (39.05) | 1.23(0.78-1.89 | 0.45 | 1.18(0.75-1.85) | 0.46 | 2.43 |
| **LDL** | | | | | | | |
| Normal (ref) | 82 (62.60) | 200 (52.77) | 1 | | 1 | | |
| High | 50 (37.40) | 179 (47.23) | 1.04 (0.93-1.17) | 0.48 | 0.96(0.89-1.02) | 0.24 | 0.07 |
| **HDL** | | | | | | | |
| Normal | 114 (87.02) | 336 (88.65) | 1 | | 1 | | |
| Low | 17 (12.98) | 43 (11.35) | 1.15 (0.63-2.09) | 0.65 | 1.14(0.61-2.13) | 0.67 | 0.07 |
| **VLDL** | | | | | | | |
| Normal (ref.) | 120 (91.60) | 347 (91.56) | 1 | | 1 | | |
| High | 11 (8.40) | 32 (8.44) | 1.00(0.83-1.20) | 0.96 | 0.97(0.91-1.04) | 0.43 | -0.02 |
| **Cholesterol** | | | | | | | |
| Normal (ref.) | 96 (73.28) | 274 (72.30) | 1 | | 1 | | |
| High | 35 (26.72) | 105 (27.70) | 1.01 (0.91-1.14) | 0.77 | 1.04(0.98-1.10) | 0.18 | 0.02 |
| **Triglyceride** | | | | | | | |
| Normal (ref.) | 103 (78.63) | 17 (4.49) | 1 | | 1 | | |
| High | 28 (21.37) | 362 (95.51) | 1.28(0.79-2.06) | 0.32 | 1.05(1.00-1.11) | 0.04* | 0.51 |
| **CRP** | | | | | | | |
| Normal (ref.) | 70 (53.44) | 182 (48.02) | 1 | | 1 | | |
| High | 61 (46.56) | 197 (51.19) | 1.05(0.95-1.17) | 0.32 | 0.95(0.88-1.03) | 0.24 | 0.13 |

**CRP:** C-reactive protein; **BMI:** Body mass index; **FPG:** Fasting plasma glucose; **HDL:** High-density lipoprotein cholesterol; **VLDL:** Very low-density lipoprotein cholesterol; **HOMA-IR:** Homeostatic model assessment of insulin resistance; **HOMA-B:** Homeostatic model of beta-cell function; **WHR:** Weight-to-height ratio; **N:** Number of participants; **COVID-19:** Coronavirus disease 2019; **CPR:** Crude prevalence ratio (univariate); **APR:** Adjusted prevalence ratio (multivariate); ***:** Significant p-value

***Predictors of insulin resistance determined by TyG***

In a similar analysis with TyG (triglyceride-glucose index) as the measure of insulin resistance, age, education and triglycerides (P < 0.05; Table 4) were the only parameters that could predict insulin resistance with the strength of the association ranging from small (education) to medium (age and triglycerides). Participant in age group 50-59 years showed a 12% increased prevalence of insulin resistance (PR: 1.12; CI: 1.02-1.21; P = 0.01; Table 4) with medium strength of the observed association. Similarly, those with tertiary level educational attainment had a 12% increased prevalence (PR: 1.12; CI: 1.01-1.26; P = 0.04; Table 4) of insulin resistance but the strength of the observed association was small. The prevalence of insulin resistance increased by only 6% (PR: 1.06; CI: 1.00-1.11; P = 0.04; Table 4) for participants with high triglycerides levels although the association demonstrated a medium strength. Interestingly, as shown in Table 4, high levels of beta-cell function (PR: 0.97; CI: 0.942-1.76; P = 0.8), FPG (PR: 1.32; CI: 0.91-1.94; P = 0.15), CRP (PR: 0.96; CI: 0.88-1.03; P = 0.24); insulin (PR: 1.16; CI: 0.74-1.83; P = 0.52), and past COVID-19 status (PR: 0.98; CI: 0.92-1.05; 0.56) did not reveal any statistically significant association with insulin resistance.

**Table 4: Predictors of insulin resistance determined by triglyceride-glucose (TyG) index**

| Parameter | No insulin resistance, N (%) | Insulin resistance, N (%) | CPR (95%CI) | P-value | APR (95%CI) | P-value | Effect size (Cohen's d) |
|---|---|---|---|---|---|---|---|
| **Location** | | | | | | | |
| Cape Coast (ref.) | 20 (37.74) | 200 (43.76) | 1 | | 1 | | |
| Tamale | 33 (62.26) | 257 (56.24) | 0.95(0.90-1.01) | 0.08 | 0.96(0.91-1.02) | 0.23 | -0.47 |
| **Sex** | | | | | | | |
| Female (ref.) | 30 (56.60) | 217 (47.48) | 1 | | 1 | | |
| Male | 23 (43.40) | 240 (52.52) | 1.02(0.97-1.09) | 0.30 | 1.05(0.91-1.10) | 0.11 | 0.27 |
| **Age** | | | | | | | |
| 20-29 (ref) | 22 (41.51) | 167 (36.54) | 1 | | 1 | | |
| 30-39 | 12 (22.64) | 151 (33.04) | 1.06(0.99-1.13) | 0.10 | 1.03(0.96-1.11) | 0.96 | 0.19 |
| 40-49 | 7 (13.21) | 85 (18.60) | 1.07(0.99-1.16) | 0.06 | 1.07(0.98-1.16) | 0.18 | 0.25 |
| 50-59 | 8 (15.09) | 28 (6.13) | 1.11(1.02-1.19) | 0.01* | 1.12(1.02-1.21) | 0.01* | 0.50 |
| 60 and above | 4 (7.55) | 26 (5.69) | 0.98(0.84-1.15) | 0.85 | 0.97(0.84-1.13) | 0.71 | -0.33 |
| **Education** | | | | | | | |
| No education (ref.) | 7 (13.21) | 69 (15.10) | 1 | | 1 | | |
| Primary | 7 (13.21) | 16 (3.50) | 1.02(0.84-1.23) | 0.87 | 1.03(0.85-1.26) | 0.73 | 0.03 |
| Secondary | 12 (22.64) | 69 (15.10) | 1.12(1.00-1.25) | 0.0.04* | 1.13(1.01-1.27) | 0.23 | 0.45 |
| Tertiary | 27 (50.94) | 303 (66.30) | 1.09(0.98-3.02) | 0.11 | 1.12(1.01-1.26) | 0.04* | 0.07 |

| | | | | | | | |
|---|---|---|---|---|---|---|---|
| **Smoke** | | | | | | | |
| No (ref.) | 51 (96.23) | 438 (95.84) | 1 | | 1 | | |
| Yes | 2 (3.77) | 19 (4.16) | 0.99(0.89-1.14) | 0.77 | 0.96(0.83-1.11) | 0.61 | -0.06 |
| **Past Covid-19** | | | | | | | |
| No (ref.) | 25 (47.17) | 230 (50.33) | 1 | | 1 | | |
| Yes | 28 (52.83) | 227 (49.67) | 1.00(0.95-1.06) | 0.87 | 0.98(0.92-1.05) | 0.56 | -0.33 |
| **HOMA-B** | | | | | | | |
| Normal (ref.) | 31 (58.49) | 221 (48.36) | 1 | | 1 | | |
| High | 10 (18.87) | 89 (19.47) | 1.11(0.99-1.24) | 0.06 | 0.97(0.942-1.76) | 0.8 | -0.49 |
| Low | 12 (22.64) | 147 (32.17) | 1.09(0.97-1.22) | 0.14 | 0.96(0.94-1.21) | 0.32 | -0.11 |
| **Waist-to-hip ratio** | | | | | | | |
| Normal (ref.) | 35 (66.04) | 261 (57.11) | 1 | | 1 | | |
| High | 18 (33.96) | 196 (42.89) | 1.12(0.98-1.09) | 0.20 | 0.92(0.91-1.21) | 0.61 | 0.01 |
| **Waist circumference** | | | | | | | |
| Normal (ref.) | 48 (90.57) | 381 (83.37) | 1 | | 1 | | |
| High | 5 (9.43) | 76 (16.63) | 1.03(0.96-1.10) | 0.38 | 0.93(0.85-1.02) | 0.14 | -0.68 |
| **BMI** | | | | | | | |
| Normal (ref.) | 15 (2.56) | 295 (64.55) | 1 | | 1 | | |
| Underweight | 14 (74.36) | 11 (2.41) | 0.99(0.78-1.25) | 0.93 | 1.02(0.80-1.31) | 0.88 | 0.2 |
| Overweight | 19 (17.95) | 109 (23.85) | 1.07(0.85-1.36) | 0.55 | 1.09(0.85-1.40) | 0.48 | -0.01 |
| Obese | 5 (5.13) | 42 (9.19) | 1.07(0.84-1.36) | 0.57 | 1.17(0.90-1.51) | 0.24 | 0.56 |
| **FPG** | | | | | | | |
| Normal (ref.) | 53 (92.45) | 439 (96.06) | 1 | | 1 | | |
| High | 0 (0.00) | 18 (3.94) | 1.42(0.93-2.15) | 0.10 | 1.32(0.91-1.94) | 0.15 | 0.47 |
| **Insulin** | | | | | | | |
| Normal (ref.) | 52 (98.11) | 309 (67.62) | 1 | | 1 | | |
| High | 1 (1.89) | 148 (32.38) | 1.21(0.68-2.13) | 0.65 | 1.16(0.74-1.83) | 0.52 | 1.21 |
| **LDL** | | | | | | | |
| Normal (ref) | 39 (73.59) | 243 (72.87) | 1 | | 1 | | |
| High | 14 (26.41) | 214 (27.13) | 1.03(0.97-1.09) | 0.36 | 0.96(0.89-1.03) | 0.25 | 0.22 |
| **HDL** | | | | | | | |
| Normal | 47 (88.68) | 402 (87.96) | 1 | | 1 | | |
| Low | 6 (11.32) | 55 (12.04) | 0.74 (0.30-1.14) | 0.48 | 0.45 (0.21-1.10) | 0.28 | -0.32 |
| **VLDL** | | | | | | | |
| Normal (ref.) | 42 (71.79) | 425 (93.00) | 1 | | 1 | | |
| High | 11 (28.21) | 32 (7.00) | 1.04(0.97-1.13) | 0.22 | 1.04(0.91-1.04) | 0.43 | 0.02 |
| **Total cholesterol** | | | | | | | |

| | | | | | | | |
|---|---|---|---|---|---|---|---|
| Normal (ref.) | 18 (10.26) | 352 (70.46) | 1 | | 1 | | |
| High | 35 (89.74) | 105 (29.54) | 1.07(1.03-1.13) | 0.002 | 1.04(0.98-1.11) | 0.18 | 0.68 |
| **Triglyceride** | | | | | | | |
| Normal (ref.) | 39 (73.59) | 344 (75.27) | 1 | | 1 | | |
| High | 14 (26.41) | 113 (24.73) | 1.07(1.02-1.12) | 0.03* | 1.06(1.00-1.11) | 0.04* | 0.63 |
| **CRP** | | | | | | | |
| Normal (ref.) | 19 (35.85) | 233 (50.98) | 1 | | 1 | | |
| High | 34 (64.15) | 224 (49.02) | 1.07(1.03-1.010) | <0.001 | 0.96(0.88-1.03) | 0.24 | -0.49 |

**CRP:** C-reactive protein; **BMI:** Body mass index; **FPG:** Fasting plasma glucose; **HDL:** High-density lipoprotein cholesterol; **VLDL:** Very low-density lipoprotein cholesterol; **HOMA-IR:** Homeostatic model assessment of insulin resistance; **HOMA-B:** Homeostatic model of beta-cell function; **WHR:** Weight-to-height ratio; **N:** Number of participants; **COVID-19:** Coronavirus disease 2019; **CPR:** Crude prevalence ratio (univariate); **APR:** Adjusted prevalence ratio (multivariate); ***:** Significant p-value

***Predictors of insulin resistance measured by HOMA-IR according to past COVID-19 status***

In a sub-group analysis based on past COVID-19 status, only the levels of fasting plasma glucose, insulin and total cholesterol could significantly (P < 0.05; Table 5) associate with insulin resistance in participants without past COVID-19 status. Participants with high levels of plasma glucose (PR: 2.06; 1.03-4.10; P = 0.03; Table 5) and insulin (PR: 1.57; CI: 1.38-1.79; P < 0.001; Table 5) exhibited 106% and 57% respective increased prevalence of insulin resistance compared with their counterparts with normal levels of the parameters. The strengths of the observed associations ranged from medium (insulin) to high (FPG). However, participants with high total cholesterol level showed a 24% reduction in prevalence of insulin resistance (PR: 0.76; CI: 0.61-0.95; P = 0.01; Table 5) with a small strength of the observed association. The seeming 19% reduction in prevalence of insulin resistance by residing in Tamale (PR: 0.81; CI: 0.70-0.95; P = 0.01) observed in the univariate analysis in participants without past COVID-19 status could not be sustained after controlling for confounders in the multivariate analysis (PR: 0.87; CI: 0.72-1.07; P = 0.19; Table 5). Compared to age 20-29 years, age 30-39 years had a 124% increased prevalence of insulin resistance (PR: 2.24, CI: 1.12-4.49; P = 0.02; Table 5) in participants without past COVID-19 status but this effect vanished after adjustment (APR: 1.13; CI: 0.94-1.30; P = 0.25; Table 5). Interestingly, none of the measured indices exhibited a statistically significant (P > 0.05; Table 5) association with insulin resistance in the participants with past COVID-19 status.

**Table 5: Predictors of insulin resistance measured by HOMA-IR according to past COVID-19 status**

| Parameter | PAST COVID-19 STATUS | | | | | NO PAST COVID-19 STATUS | | | | |
|---|---|---|---|---|---|---|---|---|---|---|
| | **CPR (95%CI)** | **P-value** | **APR (95%CI)** | **P-value** | **ES** | **CPR (95%CI)** | **P-value** | **APR (95%CI)** | **P-value** | **ES** |
| **Location** | | | | | | | | | | |
| Cape Coast **(ref.)** | 1 | | 1 | | | 1 | | 1 | | |
| Tamale | 0.96(0.84-1.10) | 0.63 | 0.99(0.87-1.13) | 0.95 | 0.20 | 0.81(0.70-0.95) | 0.01* | 0.87(0.72-1.07) | 0.19 | 0.35 |
| **Sex** | | | | | | | | | | |
| Females (ref.) | 1 | | 1 | | | 1 | | 1 | | |
| Males | 0.92(0.81-1.05) | 0.23 | 0.97(0.85-1.11) | 0.69 | 0.34 | 0.93(0.80-1.09) | 0.41 | 0.89(0.76-1.04) | 0.14 | 0.17 |
| **Age** | | | | | | | | | | |
| 20-29 **(**ref) | 1 | | 1 | | | 1 | | 1 | | |
| 30-39 | 1.50(0.75-3.02) | 0.25 | 1.10(0.93-1.30) | 0.38 | 0.10 | 2.24(1.12-4.49) | 0.02* | 1.13(0.94-1.30) | 0.25 | 0.39 |
| 40-49 | 1.10(0.93-1.33) | 0.30 | 1.08(0.90-1.30) | 0.36 | 0.24 | 1.42(0.65-3.11) | 0.38 | 1.10(0.91-1.33) | 0.34 | 0.17 |
| 50-59 | 1.18(0.92-1.52) | 0.18 | 1.11(0.84-1.47) | 0.84 | 0.10 | 3.61(0.99-13.00) | 0.05 | 1.18(0.92-1.52) | 0.18 | 0.78 |
| 60 and above | 1.10(0.83-1.46) | 0.49 | 1.02(0.77-1.35) | 0.88 | -0.07 | 0.65(0.22-1.94) | 0.44 | 1.10(0.83-1.46) | 0.49 | 0.49 |
| **Education** | | | | | | | | | | |
| No education (ref.) | 1 | | 1 | | | 1 | | 1 | | |
| Primary | 0.91(0.34-3.25) | 0.32 | 1.01(0.63-4.73) | 0.45 | 0.01 | 9.75(0.78-120.95) | 0.08 | 0.73(0.51-1.06) | 0.10 | 1.29 |
| Secondary | 0.30(0.11-1.82) | 0.51 | 0.46(0.21-2.34) | 0.32 | -0.43 | 4.10(0.44-38.42) | 0.22 | 0.83(0.68-1.00) | 0.06 | 0.79 |
| Tertiary | 0.28(0.17-2.13) | 0.21 | 0.33(0.18-3.00) | 0.12 | | 25.07(3.36-187.12 | 0.002* | 0.87(0.72-1.05) | 0.16 | 1.82 |
| **Smoke** | | | | | | | | | | |
| No **(ref.)** | 1 | | 1 | | | 1 | | 1 | | |
| Yes | 0.87(0.62-1.23) | 0.38 | 0.88(0.64-2.22) | 0.46 | -0.29 | 0.89(0.20-1.84) | 0.38 | 0.78(0.25-1.90) | 0.41 | -0.14 |
| **HOMA-B** | | | | | | | | | | |
| Normal (ref.) | 1 | | 1 | | | 1 | | 1 | | |
| High | 0.74(0.56-1.06) | 0.34 | 0.78(0.05-1.56) | 0.10 | -0.14 | 0.91(0.32-2.62) | 0.87 | 1.20(0.45-1.56) | 0.46 | 0.11 |
| Low | 0.56(0.4-1.24) | 0.64 | 0.06(0.03-1.10) | 0.13 | 0.11 | 0.12(0.04-1.35) | 0.56 | 0.56(0.06-1.11) | 0.34 | 0.01 |
| **WHR** | | | | | | | | | | |
| Normal **(ref.)** | 1 | | 1 | | | 1 | | 1 | | |
| High | 1.54(0.48-2.92) | 0.46 | 0.98(0.49-1.97) | 0.96 | -0.01 | 0.75(0.51-1.08) | 0.27 | 0.90(0.38-2.56) | 0.98 | -0.01 |
| **Waist circumference** | | | | | | | | | | |
| Normal (ref.) | 1 | | 1 | | | 1 | | 1 | | |
| High | 1.08(0.93-1.26) | 0.28 | 0.94(0.81-1.09) | 0.43 | 0.36 | 0.76(0.56-1.04) | 0.04* | 0.76(0.56-1.04) | 0.09 | -0.25 |
| **BMI** | | | | | | | | | | |

| | | | | | | | | | | |
|---|---|---|---|---|---|---|---|---|---|---|
| Normal **(ref.)** | 1 | | 1 | | | 1 | | 1 | | |
| Underweight | 0.94(0.53-1.68) | 0.84 | 0.92(0.54-1.57) | 0.77 | 0.38 | 0.89(0.56-1.39) | 0.61 | 0.91(0.58-1.44) | 0.71 | -0.23 |
| Overweight | 1.15(0.65-2.04) | 0.62 | 1.05(0.62-1.79) | 0.62 | 0.25 | 0.89(0.56-1.41) | 0.62 | 0.90(0.56-1.43) | 0.66 | -0.34 |
| Obese | 1.06(0.57-4.71) | 0.83 | 0.99(0.56-1.75) | 0.98 | 0.19 | 0.78(0.45-1.37) | 0.40 | 0.99(0.53-1.84) | 0.98 | -0.58 |
| **FPG** | | | | | | | | | | |
| Normal (ref) | 1 | | 1 | | | 1 | | 1 | | |
| High | 0.91(0.73-1.14) | 0.44 | 1.50(0.42-4.87) | 0.34 | 0.22 | 1.42(0.71-1.87) | 0.32 | 2.06(1.03-4.10) | 0.03* | 0.86 |
| **Insulin** | | | | | | | | | | |
| Normal (ref.) | **1** | | 1 | | | 1 | | 1 | | |
| High | 0.94(0.74-1.22) | 0.34 | 1.92(0.98-3.98) | 0.09 | 0.06 | 1.54(1.37-1.72) | <0.001* | 1.57(1.38-1.79) | <0.001* | 0.67 |
| **LDL** | | | | | | | | | | |
| Normal (ref.) | 1 | | 1 | | | 1 | | 1 | | |
| High | 0.94(0.80-1.10) | 0.48 | 1.08(0.87-2.32) | 0.17 | -0.02 | 1.15(0.98-1.35) | 0.08 | 1.23(0.98-1.55) | 0.06 | 0.14 |
| **HDL** | | | | | | | | | | |
| Normal | 1 | | 1 | | | | | | | |
| Low | 0.93(0.38-2.30) | 0.88 | 0.91(0.34-2.38) | 0.84 | -0.05 | 0.83(0.37-1.87) | 0.66 | 0.86(0.38-1.97) | 0.73 | -0.08 |
| **VLDL** | | | | | | | | | | |
| Normal (ref.) | 1 | | 1 | | | 1 | | 1 | | |
| High | 0.88(0.33-2.35) | 0.81 | 0.97(0.76-1.23) | 0.81 | 0.09 | 1.03(0.78-1.37) | 0.79 | 0.92(0.66-1.28) | 0.63 | 0.25 |
| **Cholesterol** | | | | | | | | | | |
| Normal **(ref.)** | 1 | | 1 | | | 1 | | 1 | | |
| High | 1.04(0.90-1.21) | 0.51 | 1.00(0.87-1.16) | 0.92 | 0.19 | 0.98(0.82-1.17) | 0.86 | 0.76(0.61-0.95) | 0.01* | -0.30 |
| **Triglyceride** | | | | | | | | | | |
| Normal **(ref.)** | **1** | | 1 | | | 1 | | 1 | | |
| High | 1.10(0.73-1.66) | 0.59 | 0.93(0.77-1.13) | 0.50 | 0.03 | 0.94(0.77-1.15) | 0.50 | 1.01(0.83-1.23) | 0.88 | 0.17 |
| **CRP** | | | | | | | | | | |
| Normal **(ref.)** | 1 | | 1 | | | 1 | | 1 | | |
| High | 1.08(0.94-1.25) | 0.21 | 1.18(0.90-1.54) | 0.21 | 0.25 | 0.98(0.83-1.15) | 0.85 | 0.96(0.82-1.12) | 0.65 | 0.04 |

**CRP:** C-reactive protein; **BMI:** Body mass index; **FPG:** Fasting plasma glucose; **HDL:** High-density lipoprotein cholesterol; **VLDL:** Very low-density lipoprotein cholesterol; **HOMA-IR:** Homeostatic model assessment of insulin resistance; **HOMA-B:** Homeostatic model of beta-cell function; **WHR:** Weight-to-height ratio; **COVID-19:** Coronavirus disease 2019; **CPR:** Crude prevalence ratio (univariate); **APR:** Adjusted prevalence ratio (multivariate); ***:** Significant p-value**; ES:** Effect size (Cohen's d)

***Predictors of insulin resistance measured by TyG according to past COVID-19 status***

In a sub-group analysis with TyG as the index for insulin resistance based on past COVID-19 status, age, BMI, triglycerides and CRP turned out as the statistically significant (P < 0.05; Table 6) predictors of insulin resistance in participants with past COVID-19 status. For participants without past COVID-19 status, age emerged as the sole predictor of insulin resistance in our setting (P < 0.05; Table 6). In general, the strength of the observed associations was small. Compared to age group 20-29 years, age 40-49 years demonstrated a 16% increased prevalence of insulin resistance (PR: 1.16; CI: 1.05-1.28; P = 0.003; Table 6) in participants with past COVID-19 status but in participants without past COVID-19 status, 11% increased prevalence of insulin resistance was observed (PR: 1.11; CI: 1.01-1.23; P = 0.03; Table 6) in age group 50-59 years. In the group with past COVID-19 status, participants with high triglycerides level compared with normal level showed a 9% increase in prevalence of insulin resistance (PR: 1.09; CI: 1.03-1.18; P = 0.01; Table 6). However, being underweight (PR: 0.86; CI: 0.77-0.95; P = 0.002; Table 6); overweight (PR: 0.88; CI: 0.78-0.99; P = 0.03; Table 6) or having high level of CRP (PR: 0.88; CI: 0.79-0.99; P = 0.03; Table 6), rather decreased the prevalence of insulin resistance compared with their counterparts with normal levels of these parameters in this group.

**Table 6: Predictors of insulin resistance measured by triglyceride-glucose index according to past COVID-19 status**

| | **PAST COVID-19 STATUS** | | | | | **NO PAST COVID-19 STATUS** | | | | |
|---|---|---|---|---|---|---|---|---|---|---|
| **Variable** | **CPR (95%CI)** | **P-value** | **APR (95%CI)** | **P-value** | **ES** | **CPR (95%CI)** | **P-value** | **APR (95%CI)** | **P-value** | **ES** |
| **Location** | | | | | | | | | | |
| Cape Coast **(**ref.) | 1 | | 1 | | | 1 | | 1 | | |
| Tamale | 0.93(0.87-1.00) | 0.1 | 0.93(0.85-1.00) | 0.06 | -0.79 | 0.64(0.23-1.76) | 0.49 | 1.00(0.89-1.13) | 0.97 | -0.06 |
| **Sex** | | | | | | | | | | |
| Females (ref.) | 1 | | 1 | | | 1 | | 1 | | |
| Males | 1.01(0.93-1.09) | 0.81 | 1.02(0.94-1.16) | 0.58 | 0.18 | 1.05(0.97-1.13) | 0.22 | 1.07(0.99-1.16) | 0.0.06 | 0.47 |
| **Age** | | | | | | | | | | |
| 20-29 **(ref)** | 1 | | 1 | | | 1 | | 1 | | |
| 30-39 | 1.07(0.97-1.18) | 0.21 | 1.04(0.95-1.16) | 0.38 | 0.39 | 1.05(00.96-1.16) | 0.30 | 1.03(0.94-1.14) | 0.54 | 0.06 |
| 40-49 | 1.15(1.06-1.24) | 0.001 | 1.16(1.05-1.28) | 0.003* | -0.03 | 0.99(0.87-1.14) | 0.94 | 0.98(0.86-1.11) | 0.73 | -0.39 |
| 50-59 | 1.06(0.90-1.26) | 0.46 | 1.09(0.93-1.29) | 0.27 | 0.38 | 1.13(1.05-1.21) | 0.001* | 1.11(1.01-1.23) | 0.03* | -0.01 |
| 60 and above | 0.91(0.70-1.20) | 0.53 | 0.91(0.70-1.19) | 0.49 | 0.21 | 1.05(0.90-1.23) | 0.50 | 0.98(0.85-1.13) | 0.82 | -0.03 |
| **Education** | | | | | | | | | | |

| | | | | | | | | | | |
|---|---|---|---|---|---|---|---|---|---|---|
| No education (ref.) | 1 | | 1 | | | 1 | | 1 | | |
| Primary | 0.95(0.78-4.18) | 0.15 | 0.98(0.70-4.00) | 0.11 | -0.01 | 1.02(0.84-1.23) | 0.87 | 1.03(0.85-1.25) | 0.76 | -0.08 |
| Secondary | 0.97(0.69-3.67) | 0.09 | 0.96(0.61-3.11) | 0.06 | -0.02 | 1.12(1.00-1.25) | 0.29 | 1.12(0.99-1.27) | 0.07 | 0.37 |
| Tertiary | 0.99(0.37-1.76) | 0.08 | 0.98(0.49-2.10) | 0.12 | -0.01 | 1.11(0.99-1.244) | 0.06 | 1.11(0.99-1.24) | 0.06 | 0.10 |
| **Smoke** | | | | | | | | | | |
| No **(ref.)** | 1 | | 1 | | | 1 | | 1 | | |
| Yes | 0.95(0.79-1.15) | 0.62 | 0.93(0.76-1.13) | 0.45 | -0.27 | 1.09(1.06-1.14) | <0.001* | 1.08(0.95-1.23) | 0.23 | |
| **HOMA-B** | | | | | | | | | | |
| Normal (ref.) | 1 | | 1 | | | 1 | | 1 | | |
| High | 0.98(0.88-2.13) | 0.12 | 0.87(0.80-1.59) | 0.18 | -0.48 | 0.22(0.10-1.69) | 0.12 | 0.34(0.21-1.20) | 0.20 | -0.86 |
| Low | 0.88(079-1.15) | 0.98 | 0.78(0.67-1.52) | 0.40 | | 0.58(0.20-1.73) | 0.33 | 0.51(0.15-1.64) | 0.28 | -0.39 |
| **WHR** | | | | | | | | | | |
| Normal **(ref.)** | 1 | | 1 | | | 1 | | 1 | | |
| High | 0.88(0.67-1.34) | 0.41 | 0.78(0.20-1.56) | 0.34 | -0.33 | 0.92(0.31-1.75) | 0.34 | 1.32(0.41-2.40) | 0.58 | 0.02 |
| **Waist circumference** | | | | | | | | | | |
| Normal (ref.) | 1 | | 1 | | | 1 | | | | |
| High | 0.99(0.90-1.09) | 0.89 | 0.90(0.78-1.03) | 0.12 | -0.65 | 1.08(0.99-1.16) | 0.05 | 0.96(0.86-1.07) | 0.47 | -0.37 |
| **BMI** | | | | | | | | | | |
| Normal (ref.) | 1 | | 1 | | | 1 | | 1 | | |
| Underweight | 0.87(0.83-0.94) | <0.001* | 0.86(0.77-0.95) | 0.002* | -0.03 | 1.09(0.70-1.70) | 0.69 | 1.15(0.73-1.79) | 0.55 | -0.63 |
| Overweight | 0.95(0.90-0.99) | 0.03* | 0.88(0.78-0.99) | 0.03* | 0.43 | 1.20(0.77-1.87) | 0.41 | 1.28(0.82-1.99) | 0.28 | 0.87 |
| Obese | 0.92(0.81-1.03) | 0.16 | 0.98(0.82-1.17) | 0.80 | 0.34 | 1.25(0.81-1.94) | 0.32 | 1.31(0.83-2.06) | 0.25 | -0.02 |
| **FPG** | | | | | | | | | | |
| Normal (ref.) | 1 | | 1 | | | 1 | | 1 | | |
| High | 1.33(0.76-2.35) | 0.32 | 1.31(0.83-2.05) | 0.24 | 0.75 | 1.19(0.55-2.62) | 0.46 | 1.17(0.57-2.34) | 0.67 | 1.11 |
| **Insulin** | | | | | | | | | | |
| Normal (ref.) | 1 | | 1 | | | 1 | | 1 | | |
| High | 1.26(0.71-2.22) | 0.42 | 1.22(0.78-1.89) | 0.39 | 0.79 | 1.02(0.93-1.11) | 0.72 | 1.00(0.91-1.09) | 0.99 | -0.03 |
| **LDL** | | | | | | | | | | |
| Normal (ref.) | 1 | | 1 | | | 1 | | 1 | | |
| High | 1.02(0.94-1.10) | 0.65 | 0.92(0.85-1.02) | 0.13 | 0.24 | 1.04(0.96-1.12) | 0.39 | 0.95(0.88-1.05) | 0.32 | 0.40 |
| **HDL** | | | | | | | | | | |
| Normal (ref.) | 1 | | 1 | | | 1 | | 1 | | |
| Low | 0.78(0.21-2.82) | 0.70 | 0.37(0.09-1.54) | 0.17 | -0.55 | 0.70(0.19-2.56) | 0.59 | 0.83(0.22-3.20) | 0.79 | -0.10 |

| | | | | | | | | | | |
|---|---|---|---|---|---|---|---|---|---|---|
| **VLDL** | | | | | | | | | | |
| Normal (ref.) | 1 | | 1 | | | 1 | | 1 | | |
| High | 1.10(1.06-1.15) | <0.001* | 0.99(0.93-1.05) | 0.71 | 0.01 | 0.97(0.82-1.15) | 0.75 | 0.96(0.81-1.13) | 0.62 | -0.54 |
| **Cholesterol** | | | | | | | | | | |
| Normal (ref.) | 1 | | 1 | | | 1 | | 1 | | |
| High | 1.06(0.99-1.14) | 0.09 | 1.02(0.92-1.10) | 0.49 | 0.47 | 1.09(1.02-1.17) | 0.01* | 1.10(0.98-1.24) | 0.08 | 1.01 |
| **Triglyceride** | | | | | | | | | | |
| Normal (ref.) | 1 | | 1 | | | 1 | | 1 | | |
| High | 1.13(1.07-1.18) | <0.001* | 1.09(1.03-1.18) | 0.01* | -0.01 | 1.02(0.93-1.11) | 0.69 | 1.00(0.91-1.10) | 0.63 | -0.01 |
| **CRP** | | | | | | | | | | |
| Normal (ref.) | 1 | | 1 | | | 1 | | 1 | | |
| High | 1.05(1.01-1.11) | 0.03* | 0.88(0.79-0.99) | 0.03* | -0.43 | 0.94(0.87-1.02) | 0.17 | 0.95(0.88-1.03) | 0.28 | -0.42 |

**CRP:** C-reactive protein; **BMI:** Body mass index; **FPG:** Fasting plasma glucose; **HDL:** High-density lipoprotein cholesterol; **VLDL:** Very low-density lipoprotein cholesterol; **HOMA-IR:** Homeostatic model assessment of insulin resistance; **HOMA-B:** Homeostatic model of beta-cell function; **WHR:** Weight-to-height ratio; **N:** Number of participants; **COVID-19:** Coronavirus disease 2019; **CPR:** Crude prevalence ratio (univariate); **APR:** Adjusted prevalence ratio (multivariate); ***:** Significant p-value**; ES:** Effect size (Cohen's d)

## DISCUSSION

Results from our analyses show that irrespective of the surrogate marker of insulin resistance or past COVID-19 status, age, educational level and triglycerides could significantly associate with insulin resistance.

In participants without past COVID-19 status, fasting plasma glucose, insulin and total cholesterol could significantly associate with insulin resistance measured by HOMA-IR. In these participants, high levels of plasma glucose and insulin exhibited 106% and 57% respective increased prevalence of insulin resistance compared with their counterparts with normal levels of the parameters. However, high total cholesterol level showed a 24% reduction in prevalence of insulin resistance. Irrespective of past COVID-19 status and residential location, 74.31% prevalence of insulin resistance was observed with HOMA-1R as the surrogate marker.

Using TyG as the index for insulin resistance, age, BMI, triglycerides and CRP were the significant predictors of insulin resistance in participants with past COVID-19 status. In these participants, being in age 40-49 years compared to age 20-29 years and having high triglycerides level compared to the normal level increased the prevalence of insulin resistance. However, being underweight or overweight compared to normal weight and having high level of CRP

compared to the normal level decreased the prevalence of insulin resistance. For participants without past COVID-19 status, age group 50-59 years compared with 20-29 years increased the prevalence of insulin resistance as measured by TyG. Irrespective of past COVID-19 status and residential location, TyG-determined insulin resistance prevalence was 89.61%.

Insulin resistance remains a cardinal risk factor for the development of T2DM. The gold standard for the assessment of insulin resistance is the glucose clamp technique developed by DeFronzo et al. [17]. However, the method is expensive, laborious and requires high level of technical expertise, making it unsuitable for application in large-sample studies and routine clinical practice. Therefore, various less expensive and simple techniques that rely on surrogate markers have been developed for effective assessment of insulin resistance. The homeostatic model developed by Matthews et al. [14] to assess insulin resistance (HOMA-IR) and beta-cell function (HOMAB) and the triglyceride-glucose index (TyG) [15] are some of such methods that have been validated against the gold standard and found to be appropriate for assessing insulin resistance under fasting steady state [18, 19]. Insulin resistance is affected by race and other environmental factors, which differ from place to place. In the current study, we sought to examine determinants of insulin resistance, assessed by two surrogate markers, in non-diabetic participants with past COVID-19 status compared with their counterparts without any past COVID-19 status in two regional capitals of Ghana. The insulin resistance prevalence of 74.31% and 89.61% as determined by HOMA-IR and TyG respectively in our sample are higher than 38% reported in Lebanon [20], 46.4% in Venezuela [21], and 60.2% in Bangladesh [22]. This extremely high prevalence of insulin resistance exemplifies the risk of development of T2DM in our setting under favourable conditions. Considering that insulin resistance cuts across the various weight groups in our setting, with majority (52.25%) of participants within the normal weight category, other factors beyond being overweight or obese could be accounting for our observation. Indeed, irrespective of the surrogate marker used or past COVID-19 status, the prevalence of insulin resistance could not be predicted by body mass index, waist circumference, waist-to-hip ratio, C-reactive protein, smoking and alcohol intake in our setting although high level of circulating triglycerides increased the observed prevalence. Our findings appear contrary to those of previous studies [23-29] that associated indices of obesity and inflammation with insulin resistance. However, the observed positive association of insulin resistance with triglycerides level irrespective of past COVID-19 status or surrogate marker used in the current study is in support of studies [25, 29] that associated insulin resistance with lipid metabolism in furtherance of the established

diabetogenic role of adiposity [30]. Indeed, white adipose tissue has long been implicated in the development of insulin resistance through production of adipocytokines and other lipid derivatives that impair insulin signaling [31-33].

A recent large-sample comprehensive epidemiological study which included 5340 participants and quantified 78 metabolic measures reported that non-diabetic individuals with insulin resistance are exposed to similar adverse metabolic environment in non-fasting state as their diabetic counterparts [34]. This observation implies that the metabolic response of the insulin resistant individual in the fed state may be even more important to the overall health than that of the fasting state used in various diagnostic decisions. Molecular and cellular evidence of IR show that the phenomenon results from altered phosphorylation in the insulin-signaling pathway in different cells and organs with overall adverse effects on carbohydrate, protein and lipid metabolism and associated health risk [35, 36]. Therefore, the failure of the above indices of obesity and inflammation to predict insulin resistance assessed under fasting state in our setting irrespective of past COVID-19 status or surrogate marker of insulin resistance does not imply that those biochemical processes are not involved at the molecular level with respect to the development of the condition. The difference in findings between the current study and the previous ones [23-29] could be ascribed to variations in participants characteristics. Indeed, none of these studies [23-29] involved individuals who had recovered from COVID-19. Indeed, sub-group analyses based on past COVID-19 status revealed a seeming protection against insulin resistance prevalence in both underweight and overweight participants with past COVID-19 status only. This finding portrays the complexity of the role of adiposity in the development of insulin resistance in our context.

However, the observation of insulin resistance across the various weight groups corroborates a number of previous studies [20-27] and implies that the development of insulin resistance probably precedes weight gain. Indeed, in spite of variations in mean or median levels of some of the measured biochemical indices between the two study groups, the observed levels were generally within the normal range, further supporting the notion that the development of insulin resistance does not necessarily require high levels of such indices. High levels of such indices may rather worsen an already existing condition. For instance, low-grade inflammation, is known to be associated with insulin resistance [37, 38] implying that a high grade version may aggravate the already established condition. Interestingly, the seeming promotion of insulin resistance by high levels of C-reactive protein, which could not be sustained when TyG was the surrogate marker, in the entire sample, irrespective of COVID-19 status, was rather sustained only in the sub-group analysis in participants with past COVID-19 status. This observation, together with the various predictors

of insulin resistance identified by the two surrogate markers points to the complexity of the phenomenon and its associated factors.

Interestingly, a seeming protection from HOMA-IR-determined insulin resistance by tertiary education in the entire participants irrespective of past COVID-19 status changed to facilitation with TyG index as the surrogate marker although age 50-59 years was identified as a facilitator of insulin resistance by both surrogate markers in our setting. Moreover, high insulin level could only be found to be an important promoter of HOMA-IR-determined insulin resistance in participants without prior COVID-19 status in our context. Our observations in respect of the association of insulin resistance with high insulin level and advanced age appear to support previous studies [39, 40]. High insulin level can be a consequence of insulin resistance but it can also be an independent primary defect indicative of an unfavourable metabolic health [41, 42]. High insulin level is influenced by several factors including age, race, genetics, adiposity, free fatty acid levels, insulin secretion and clearance and it is self-perpetuating [41, 43]. A cell-based study [44] has long demonstrated that chronic exposure of insulin receptors to high levels of insulin impairs insulin signaling with time through altered autophosphorylation without affecting the quantity of receptors. This finding, which has been corroborated by other studies in humans [41, 43] as well as the current one, suggests that once hyperinsulinemic-insulin resistance cycle is established, metabolic health is adversarially affected if allowed to persist. Although a recent study by Stephens et al. [40] associated metabolic health with age and higher education, the nature of the reported association, notably, for education, differs from that of the current study in the context of TyG-determined insulin resistance. In the current study, tertiary education was positively associated with TyG-determined insulin resistance as opposed to the previous study [40] that reported a negative relationship. However, in terms of HOMA-IR-determined insulin resistance, our findings corroborate those of Stephens et al. [40]. As reported in previous studies [45, 46], the attainment of a tertiary education qualification is virtually a pre-requisite for decent employment notably in the formal sector of the Ghanaian economy. Moreover, with the retirement age being 60 years in Ghana, age 50-59 years corresponds with the age range at which participants will likely be at the peak of their career where various incentives that promote job-related sedentariness is prevalent. Such a situation can create a conducive metabolic environment for the development of insulin resistance as observed in the current study.

Interestingly, with TyG index as the surrogate marker, a sub-group analyses showed that in participants with past COVID-19 status, apart from age, formal education and BMI that associated with the observed insulin resistance, high triglycerides level promoted but high CRP level protected against the phenomenon. Whereas the protection offered by high CRP level deviates from the expected facilitation role [37, 38], it further demonstrates the complexity of the relationship between CRP and the insulin resistance phenomenon.

Fasting plasma glucose (FPG) and beta-cell function (HOMA-B) are critical factors that are known to be associated with insulin resistance [47-49]. High FPG could be a consequence of insulin resistance or reduced beta-cell function or both although glucose-induced insulin resistance [47, 48] is possible, and can create a hyperglycaemia-insulin resistance cycle that requires an intervention to ensure a favourable metabolic health. Therefore, in the current study where insulin resistance is highly prevalent, participants with high fasting plasma glucose are expected to have a heightened risk of developing T2DM. High beta-cell function may demonstrate some protection against development of insulin resistance in the short-term but increases the diabetogenic risk in the long-term due to its probable unsustainable nature through exhaustion [49]. Irrespective of past COVID-19 status, neither FPG nor HOMA-B was associated with insulin resistance in our setting when TyG was used as the index for insulin resistance. However, with HOMA-IR as the surrogate marker, FPG in addition to insulin and total cholesterol levels associated with insulin resistance in participants without past COVID-19 status. This identification of different predictive variables of insulin resistance under different surrogate markers reflects the complexity of the phenomenon and its contributory factors [50].

Our study is not without limitations. Being a cross-sectional study, causal relationships could not be established. Also, detailed information on other lifestyle factors such as exercise and dietary information which can affect insulin resistance were not available. Above all, the use of fasting samples that are subject to fluctuations call for caution in undue extrapolation of our findings to populations of different characteristics. Therefore, longitudinal studies with wider coverage and larger sample size involving additional biomarkers that assess both fasting and postprandial insulin and glucose indices may be necessary to better understand the promoting factors of insulin resistance in our context. In spite of the above limitations, the current study has provided evidence of factors that affect the development of insulin resistance in participants with and without past COVID-19 status in our setting.

## CONCLUSION

The extremely high prevalence of insulin resistance in both groups of participants in addition to dyslipidemias and abnormal beta-cell function connotes increased diabetogenic risk. Irrespective of surrogate marker or past COVID-19 status, age, education and triglycerides levels associated with insulin resistance. In participants without prior COVID-19 status, FPG, insulin and total cholesterol associated with HOMA-IR-determined insulin resistance. However, with TyG index, age, BMI, triglycerides and CRP were the significant determinants of insulin resistance in participants with past COVID-19 status. The use of HOMA-IR and TyG as surrogate markers for insulin resistance allowed for the identification of a wide range of predictors of the condition in our context, which could not have been possible if only one surrogate marker had been used. Further studies are needed to unravel additional factors that influence the development of insulin resistance under fasting and postprandial states in apparently healthy adults in our setting even as health educational measures on lifestyle changes that improve insulin sensitivity are intensified.

### Authors' contribution

**ASB, LDK, CA, JAA and SA** contributed to conception, design, analysis and interpretation, drafting of the manuscript and critical revision for important intellectual content. **EAB** contributed to data analysis and interpretation, drafting of the manuscript and critical revision for important intellectual content. **JAO and RKA** were involved in the design, data collection, analysis and interpretation. All authors read and approved the final manuscript for publication.

### Declaration of interests

We declare that we have no known competing financial interests or personal relationships that could have appeared to influence the work reported in this paper.


### Acknowledgement

The authors are very grateful to the study participants for their unique contribution in ensuring the success of this research work. Additionally, we are grateful to the staff and management of the Cape Coast and Tamale Teaching Hospitals for allowing their facilities to be used as sites for the study. Above are we are thankful to the staff and management of the Public Health Reference Laboratory of the Tamale Teaching Hospital for their support in the conduct of the study.

## REFERENCES


1. Ghana Health Service. COVID-19: Ghana's outbreak response and management updates [cited 15th February 2024]. Available from www.ghs.gov.gh/covid19/
2. International Diabetes Federation. IDF diabetes atlas. 10th ed. Brussels: International Diabetes Federation; 2021.
3. Acquah S. Implications of COVID-19 pandemic on evolution of diabetes in malaria-endemic African region. J Diabetes Res. 2020;2020:8205261. doi:10.1155/2020/8205261.
4. d'Annunzio G, Maffeis C, Cherubini V, Rabbone I, Scaramuzza A, Schiaffini R, et al. Caring for children and adolescents with type 1 diabetes mellitus: Italian Society for Pediatric Endocrinology and Diabetology (ISPED) statements during COVID-19 pandemia. Diabetes Res Clin Pract 2020;168:108372. doi:10.101/j.diabres.2020.108372.
5. Rabbone I, Schiaffini R, Cherubini V, Maffeis C, Scaramuzza A; Diabetes Study Group of the Italian Society for Pediatric Endocrinology and Diabetes.: has COVID-19 delayed the diagnosis and worsened the presentation of type 1 diabetes in children? Diabetes Care. 2020;43(11): 2870–2872. doi: 10.2337/dc20-1321.
6. Qeadan F, Tingey B, Egbert J, Pezzolesi MG, Burge MR, Peterson KA, et al. The associations between COVID-19 diagnosis, type 1 diabetes, and the risk of diabetic ketoacidosis: a nationwide cohort from the US using the Cerner Real-World Data. PLoS One. 2022;17(4):e0266809. doi: 10.1371/journal.pone.0266809.
7. Nassar M, Nso N, Baraka B, Alfishawy M, Mohamed M, Nyabera A, et al. The association between COVID-19 and type 1 diabetes mellitus: a systematic review. Diabetes Metab Syndr. 2021;15(1):447–454. doi: 10.1016/j.dsx.2021.02.009.
8. Mastromauro C, Blasetti A, Primavera M, Ceglie L, Mohn A, Chiarelli F, et al. Peculiar characteristics of new-onset type 1 diabetes during COVID-19 pandemic. Ital J Pediatr. 2022;48(1):26. doi: 10.1186/s13052-022-01223-8.
9. Chen M, Zhu B, Chen D, Hu X, Xu X, Shen WJ, et al. COVID-19 may increase the risk of insulin resistance in adult patients without diabetes: a 6-month prospective study. Endocr Pract. 2021;27(8): 834-841. doi: 10.1016/j.eprac.2021.04.004.
10. Santos A, Magro DO, Evangelista-Poderoso R, Saad MJA. Diabetes, obesity, and insulin resistance in COVID-19: molecular interrelationship and therapeutic implications. Diabetol Metab Syndr. 2021;13(1):23. doi: 10.1186/s13098-021-00639-2.
11. Cohen DH, LeRoith D. Obesity, type 2 diabetes, and cancer: the insulin and IGF connection. Endocr-Relat. Cancer 2012;19(5): F27–F45. doi: 10.1530/ERC-11-0374.
12. Kim HY. Statistical notes for clinical researchers: sample size calculation 1. comparison of two independent sample means. Restor Dent Endod. 2016;41:74-78. doi: 10.5395/rde.2016.41.2.154.
13. Adatsi RK., Bockarie AS, Derkyi-Kwarteng L, Pappoe F, Nsiah P, Dankwa K, et al. Increased insulin resistance with reduced beta cell function in recovered COVID-19 Ghanaians. Endocri Metab Sci. 2023;13: 100150. doi: 10.1016/j.endmts.2023.100150.
14. Matthews DR, Hosker JP, Rudenski AS, Naylor BA, Treacher DF, Turner RC. Homeostasis model assessment: insulin resistance and $\beta$-cell function from fasting plasma glucose and insulin concentrations in man. Diabetologia 1985; 28(7): 412–419. doi: 10.1007/BF00280883.
15. Simental-Mendía LE, Rodríguez-Morán M, Guerrero-Romero F. The product of fasting glucose and triglycerides as surrogate for identifying insulin resistance in apparently healthy subjects. Metab Syndr Relat Disord 2008;6(4): 299-304. doi: 10.1089/met.2008.0034.
16. Expert Panel on Detection, Evaluation, and Treatment of High Blood Cholesterol in Adults. Executive summary of the third report of the National Cholesterol Education Program (NCEP) Expert Panel on Detection, Evaluation, and Treatment of High Blood Cholesterol in Adults (Adult Treatment Panel III). JAMA. 2001;285(19): 2486-2497. doi: 10.1001/jama.285.19.2486.
17. DeFronzo RA, Tobin JD, Andres R. Glucose clamp technique: a method for quantifying insulin secretion and resistance. Am J Physiol. 1979;237(3): E214-223. doi: 10.1152/ajpendo.1979.237.3.E214.
18. Guerrero-Romero F, Simental-Mendía LE, González-Ortiz M, Martínez-Abundis E, Ramos-Zavala MG, Hernández-González SO, et al. The product of triglycerides and glucose, a simple measure of insulin sensitivity. Comparison with the euglycemic-hyperinsulinemic clamp. J Clin Endocrinol Metab. 2010;95(7):3347-3351. doi: 10.1210/jc.2010-0288.

19. Luo P, Cao Y, Li P, Li W, Song Z, Fu Z, et al. TyG index performs better than HOMA-IR in Chinese type 2 diabetes mellitus with a BMI < 35 kg/m$^2$: a hyperglycemic clamp validated study. Medicina (Kaunas). 2022;58(7):876. doi: 10.3390/medicina58070876.
20. Fahed M, Abou Jaoudeh MG, Merhi S, Mosleh JMB, Ghadieh R, Al Hayek S, et al. Evaluation of risk factors for insulin resistance: a cross sectional study among employees at a private university in Lebanon. BMC Endocr Disord. 2020;20(1):85. doi: 10.1186/s12902-020-00558-9.
21. Bermudez V, Salazar J, Martínez MS, Chávez-Castillo M, Olivar LC, Calvo MJ, et al. Prevalence and associated factors of insulin resistance in adults from Maracaibo City, Venezuela. Adv Prev Med. 2016;2016:9405105. doi: 10.1155/2016/9405105.
22. Sejooti SS, Naher S, Hoque MM, Zaman MS, Aminur Rashid HM. Frequency of insulin resistance in nondiabetic adult Bangladeshi individuals of different obesity phenotypes. Diabetes Metab Syndr. 2019;13(1):62-67. doi: 10.1016/j.dsx.2018.08.022.
23. Ozdemir O. Evaluation of waist circumference, waist-hip ratio and waist-length ratio in insulin resistance: which is a powerful predictor? Ann of Med Res 2023;30(5):533–537.
24. Cho SH, Jeong SH, Shin J, Park S, Jang SI. Short-term smoking increases the risk of insulin resistance. Sci Rep. 2022;12(1):3550. doi: 10.1038/s41598-022-07626-1.
25. Elrayess MA, Rizk NM, Fadel AS, Kerkadi A. Prevalence and predictors of insulin resistance in non-obese healthy young females in Qatar. Int J Environ Res Public Health. 2020;17(14):5088. doi: 10.3390/ijerph17145088.
26. Kim GR, Choi DW, Nam CM, Jang SI, Park EC. Synergistic association of high-sensitivity C-reactive protein and body mass index with insulin resistance in non-diabetic adults. Sci Rep. 2020;10(1):18417. doi: 10.1038/s41598-020-75390-1.
27. Jamar G, Almeida FR, Gagliardi A, Sobral MR, Ping CT, Sperandio E, et al. Evaluation of waist-to-height ratio as a predictor of insulin resistance in non-diabetic obese individuals. A cross-sectional study. Sao Paulo Med J. 2017;135(5): 462-468. doi: 10.1590/1516-3180.2016.0358280417.
28. Oumer A, Ale A, Tariku Z, Hamza A, Abera L, Seifu A. Waist-to-hip circumference and waist-to-height ratio could strongly predict glycemic control than body mass index among adult patients with diabetes in Ethiopia: ROC analysis. PLoS One. 2022;17(11):e0273786. doi: 10.1371/journal.pone.0273786.
29. Chen W, Ding S, Tu J, Xiao G, Chen K, Zhang Y, Huang R, Liao Y. Association between the insulin resistance marker TyG index and subsequent adverse long-term cardiovascular events in young and middle-aged US adults based on obesity status. Lipids Health Dis. 2023 May 18;22(1):65. doi: 10.1186/s12944-023-01834-y.
30. Gastaldelli A, Gaggini M, DeFronzo RA. Role of adipose tissue insulin resistance in the natural history of type 2 diabetes: Results from the San Antonio Metabolism Study. Diabetes. 2017;66(4): 815-822. doi: 10.2337/db16-1167.
31. McArdle MA, Finucane OM, Connaughton RM, McMorrow AM, Roche HM. Mechanisms of obesity-induced inflammation and insulin resistance: insights into the emerging role of nutritional strategies. Front Endocrinol (Lausanne). 2013 ;4:52. doi: 10.3389/fendo.2013.00052.
32. Sethi JK, Vidal-Puig AJ. Thematic review series: adipocyte biology. Adipose tissue function and plasticity orchestrate nutritional adaptation. J Lipid Res. 2007;48(6): 1253-1262. doi: 10.1194/jlr.R700005-JLR200.
33. Gual P, Le Marchand-Brustel Y, Tanti JF. Positive and negative regulation of insulin signaling through IRS-1 phosphorylation. Biochimie. 2005;87(1):99-109. doi: 10.1016/j.biochi.2004.10.019.
34. Wang Q, Jokelainen J, Auvinen J, Puukka K, Keinanen-Kiukaanniemi S, Jarvelin MR, et al. Insulin resistance and systemic metabolic changes in oral glucose tolerance test in 5340 individuals: an interventional study. BMC Med 2019;17(1):217. doi: 10.1186/s12916-019-1440-4.
35. da Silva Rosa S, Nayak N, Caymo AM, Gordon JW. Mechanisms of muscle insulin resistance and the cross-talk with liver and adipose tissue. Physiol Rep. 2020;8:e14607. doi: 10.14814/phy2.14607.
36. Lee SH, Park SY, Choi CS. Insulin resistance: from mechanisms to therapeutic strategies. Diabetes Metab J. 2022;46(1):15-37. doi: 10.4093/dmj.2021.0280.
37. Kawai T., Autieri M.V., Scalia R. Adipose tissue inflammation and metabolic dysfunction in obesity. Am J Physiol Cell Physiol. 2021;320:C375–C391. doi: 10.1152/ajpcell.00379.2020.
38. Püschel GP, Klauder J, Henkel J. Macrophages, low-grade inflammation, insulin resistance and hyperinsulinemia: a mutual ambiguous relationship in the development of metabolic diseases. J Clin Med. 2022;11(15):4358. doi: 10.3390/jcm11154358.
39. Vaidya RA, Desai S, Moitra P, Salis S, Agashe S, Battalwar R, et al. Hyperinsulinemia: an early biomarker of metabolic dysfunction. Front Clin Diabetes Healthc. 2023;4:1159664. doi: 10.3389/fcdhc.2023.1159664

40. Stephens CR, Easton JF, Robles-Cabrera A, Fossion R, de la Cruz L, Martínez-Tapia R, et al. The impact of education and age on metabolic disorders. Front Public Health. 2020;8:180. doi: 10.3389/fpubh.2020.00180.
41. Thomas DD, Corkey BE, Istfan NW, Apovian CM. Hyperinsulinemia: an early indicator of metabolic dysfunction. J Endocr Soc. 2019;3(9): 1727-1747. doi: 10.1210/js.2019-00065.
42. Tricò D, Natali A, Arslanian S, Mari A, Ferrannini E. Identification, pathophysiology, and clinical implications of primary insulin hypersecretion in nondiabetic adults and adolescents. JCI Insight. 2018;3(24):e124912. doi: 10.1172/jci.insight.124912.
43. Fryk E, Olausson J, Mossberg K, Strindberg L, Schmelz M, Brogren H, et al. Hyperinsulinemia and insulin resistance in the obese may develop as part of a homeostatic response to elevated free fatty acids: A mechanistic case-control and a population-based cohort study. EBioMedicine. 2021;65:103264. doi: 10.1016/j.ebiom.2021.103264.
44. Catalano KJ, Maddux BA, Szary J, Youngren JF, Goldfine ID, Schaufele F. Insulin resistance induced by hyperinsulinemia coincides with a persistent alteration at the insulin receptor tyrosine kinase domain. PLoS One. 2014;9(9):e108693. doi: 10.1371/journal.pone.0108693.
45. Quadlin N, VanHeuvelen T, Ahearn CE. Higher education and high-wage gender inequality. Soc Sci Res. 2023 May;112:102873. doi: 10.1016/j.ssresearch.2023.102873.
46. Ali MS, Jalal H. Higher Education as a Predictor of Employment: The World of Work Perspective. Bull Educ Res. 2018; 40(2): 79-90.
47. Zhang W, Liu J, Tian L, Liu Q, Fu Y, Garvey WT. TRIB3 mediates glucose-induced insulin resistance via a mechanism that requires the hexosamine biosynthetic pathway. Diabetes. 2013;62(12): 4192–4200. doi: 10.2337/db13-0312.
48. Kawanaka K, Han D-H, Gao J, Nolte LA, Holloszy JO. Development of glucose-induced insulin resistance in muscle requires protein synthesis. J Biol Chem. 2001;276(23): 20101–20107. doi: 10.1074/jbc.M010599200.
49. Esser N, Utzschneider KM, Kahn SE. Early beta cell dysfunction vs insulin hypersecretion as the primary event in the pathogenesis of dysglycaemia. Diabetologia. 2020;63(10):2007-2021. doi: 10.1007/s00125-020-05245-x.
50. Detopoulou P, Demopoulos CA, Antonopoulou S. Micronutrients, phytochemicals and Mediterranean diet: A potential protective role against COVID-19 through modulation of PAF actions and metabolism.Nutrients. 2021; 13(2): 462. doi: 10.3390/nu13020462.

**RESPONSE TO REVIEWERS COMMENTS**

Please, the information in Arial font size 14 are the exact comments made by the esteemed Reviewers. We have presented our response in font size 12 of Times New Roman in all cases, please.

## Title

The title is "Prevalence and determinants of Insulin resistance in residents of two regional capitals in Ghana: An observational study".

I believe the title can be improved to reflect the study's aim of comparing insulin resistance prevalence and determinants among individuals exposed to Covid-19 and those not exposed. Since the focus is on analysing differences based on **COVID-19 exposure**, it would be good to reflect this aspect in the title.

Please, this suggestion has been duly noted and accepted. The title has therefore been modified to "Prevalence and Determinants of Insulin Resistance in Recovered COVID-19 and Uninfected Residents of Two Regional Capitals in Ghana: An Observational Study" to reflect the suggestion, please (page 1 of revised manuscript).

The title seems to imply that the study was community-based and conducted in two regional capitals in Ghana. Is this correct? However, the methods suggest that the study was hospital-based. Could you please clarify this discrepancy and ensure that the correct assertion is reflected consistently throughout the manuscript?

In hospital-based studies, participant recruitments, questionnaire administration and measurement of physical indices occur within the hospital environment. In our case, participants recruitments, questionnaire administration and measurement of physical indices mostly occurred outside the hospital terrain. However, hospital records for the recovered COVID-19 participants were relied upon for identification and invitation for recruitment after successful screening at a place of convenience for the participant. Moreover, the laboratory analyses were carried out in the hospitals. The control participants were recruited from the community. In this regard, we take the view that our study was largely a community-based type and have modified the write-up to reflect this, please.

## ABSTRACT

In the **background,** consider replacing ‘devastative’ with “devastating”.

What is the difference between COVID-19 exposure, COVID-19 infection and COVID-19 disease? The last statement in the abstract background states “The current study sought to compare the factors that determine insulin resistance in Ghanaians with and **without COVID-19 exposure**, resident in the Cape Coast and Tamale metropolitan areas”, while the last statement in the introduction section of the main manuscript states “the current study aims to identify determinants of insulin resistance in a Ghanaian population that has recovered completely from clinically mild cases of COVID-19 and their counterparts that never had a clinical history of the condition in two tertiary facilities”.

We could not find the word devastative in the background of the abstract. However, we have modified the abstract appropriately for improved clarity, please (page 2 of revised manuscript). Also, we have clarified the terminologies better to reflect those who had recovered from the COVID-19 condition compared with those without any history of the condition. For instance, the phrase ‘without COVID-19 exposure’ has been replaced with ‘without past COVID-19 status’. The use of the term ‘COVID-19 exposure’ has been avoided completely in the revised manuscript. We are hopeful that these modifications together with others have improved clarity to acceptable levels in the revised manuscript, please.

The authors did not clearly state the eligibility criteria in the methods section of the main manuscript. It appears that the term “**without COVID-19 exposure”** as stated in the abstract, is described in the methods section as “those **without a history of the COVID-19 infection who** originated from the same communities as their recovered counterparts but were **never diagnosed with the condition**”. However, this definition of “without a history of the COVID19 infection” inherently includes individuals who may have been **potentially exposed** but undiagnosed or **exposed** but asymptomatic. How did you address the potential issue of misclassification in this context?

Please, we have improved the clarity of the eligibility criteria in the main manuscript (pages 4 and 5 of revised manuscript). Our study focused on individuals who had recovered from previously diagnosed COVID-19 condition compared with those without any history of COVID-19 diagnosis. In Ghana, diagnosis of COVID-19 was made only after presenting with appropriate clinical signs and symptoms coupled with a positive COVID-19 PCR test irrespective of severity of associated

signs and symptoms. All others who did not meet these criteria were considered uninfected as far as COVID-19 is concerned. Therefore we have avoided the use of the terms COVID-19 exposure/exposed or unexposure/unexposed completely from the revised manuscript to eliminate the seeming confusion, please. With this approach, the possibility of misclassification is eliminated, please.

Did the authors also ascertain that this category of “without COVID-19 exposure” had not been in contact with either a confirmed or suspected or case exposed to a high-risk environment? If they did, please describe how this was ascertained. This is because in their criteria they considered only “history of COVID-19 infection” and “never diagnosed” as highlighted above. Please provide a clarification of these issues.

As has been earlier indicated, our focus was on those with a history of recovery from confirmed diagnosis of COVID-19 by clinical and laboratory evidence compared with those without any such history in the same community. We made this determination through questionnaire administration and relevant hospital records. We therefore did not ascertain prior contact with confirmed or suspected case, please. Moreover, conducting our study with participants who had recovered from COVID-19 condition for more than six months, we were convinced that the risk is low even if past contacts with active COVID-19 patients had been made. Above all, all participants had been vaccinated against the COVID-19 condition before enrollment on the study. With all these measures, we are of the respectful firm conviction that those with no history of the COVID-19 condition never had the condition since no verifiable evidence could be provided, please. We are, however, hopeful that, the modifications made in the revised manuscript have clearly addressed any ambiguity in the earlier version of our manuscript, please.

In the **methods**, please provide a brief description of the data collection methods and indicate the outcome variable and the independent variables. Additionally, briefly describe the conduct of data analysis to assess the prevalence and determinants, including the measures of association and level of statistical significance.

Please we have addressed these concerns in the body of the revised manuscript (pages 6 and 7). In the data analysis section of the previous and the revised version of the manuscript (page 9), such issues have been duly addressed, please. Because of journal limitation on the length of the abstract, all these issues could not be captured in the abstract, please.

In the **results**, please provide the names of the specific surrogate markers used along with their related prevalence and confidence intervals.

Authors stated that "Prevalence of insulin resistance ranging from **74.31%** to **89.61%** depending on the surrogate marker used coupled with low (**31.18%)** and high (**19.41%)** betacell function, were observed". I suggest the authors present the results of the specific surrogate markers rather than combining them in a range as above.

We have addressed this concern in the abstract and in the main document of the revised manuscript, please (pages 2, 11 and 12).

Please, as per the title, provide results on the prevalence of insulin resistance among those exposed to COVID-19 and those not exposed to COVID-19.

Please, this has been addressed in page 2 of the revised manuscript.

I think it would be more informative to break down the dyslipidemias individually, rather than combining the percentages into a range (8.31 % - 44.71%).

Please, this concern has been addressed in the abstract and other sections of the revised manuscript (pages 2, 11 and 12).

Please present the measures of association with their confidence intervals. If the outcome of interest is above 10%, is it appropriate to use Odds Ratios as a measure of association?

Please, all the relevant measures of association were presented with their 95% confidence intervals in the earlier version (Tables 3-6) and have been repeated in the revised manuscript (Tables 3-6; pages 13-21). In terms of the question about the prevalence threshold of 10% beyond which odds ratios become unsuitable for measures of association, we have noted a number of publications (Tamhane et al., 2016; Martinez et al., 2017) that have alluded to this view with the prevalence ratio suggested as a suitable alternative. To this end we have replaced the odds ratio with the prevalence ratio in the revised manuscript despite our belief that not much differences were observed.

**Conclusion:** Please review the conclusion.
Is there evidence from this study that "educating the adult populace to adopt lifestyle measures can reduce insulin resistance"?

Please, no direct evidence was provided in the current study to that effect. We made the proposition based on the high prevalence of insulin resistance observed in our work. We have therefore modified the conclusion in the revised manuscript (page 2) line with the suggestion, please.

## INTRODUCTION

In the last paragraph, on page 4, the authors stated the aim as "to identify determinants of insulin resistance using two surrogate markers in a Ghanaian population that had recovered completely from clinically mild cases of COVID-19 and their counterparts that never had a clinical history of the condition in two tertiary facilities".

I suggest authors maintain consistency in the use of terms, particularly the exposure variable related to COVID-19 status. The aim stated in the introduction differs from the one provided in the abstract. I think not having a clinical history of COVID-19 is not the same as not being exposed to Covid 19, as I indicated in the abstract comments. Please consider and clarify.

Please, appropriate clarifications have been made in the revised manuscript (pages 2 and 4) to ensure the needed consistency.

## METHODS

**Study site:** What was the rationale for choosing Cape Coast (Teaching Hospital (CCTH) and Tamale metropolis (Tamale Teaching Hospital (TTH), sites which have "differing geography, climate, ethnicity and dietary norms" as study sites?

The Cape Coast and Tamale sites were chosen to have an idea of regional variation, if any, of the impact of COVID-19 on the Ghanaian populace in terms of diabetogenic risk, please.

Please add a location map of the study sites.

Please, this has been done as suggested and labeled as figure 1 in the revised manuscript (page 5).

## Can you clearly state that the study was either a community-based study or a facility-based study?

Please, our study was a community-based type although we were guided by hospital data for recruitment of participants with past COVID-19 status. Also, the laboratory measurements were done at the hospitals. However, participants were not required to visit the hospital for samples to be taken, please.

## Describe the eligibility criteria and clearly outline the inclusion and exclusion criteria. Why did you select your participants to be above 20 years old and not adults 18 years and above?

Please, the study targeted the apparently healthy adult population in the two metropolis. The criteria for inclusion and exclusion have been clarified in the revised manuscript (pages 4 and 5). The selection of 20 years and above was informed by the minimum age of the participants with past COVID-19 status, please.

## You stated that the study population comprised participants "...... without underlying health conditions at the time of recruitment". Please describe how the screening was done to ensure that you met this criterion.

The screening for inclusion of participants was done by questionnaire administration. Participants were asked if they had been diagnosed of any chronic condition or treating an existing condition. Based on the responses, appropriate decisions were made to recruit or not after consent. Trained and experienced health professionals were employed in this exercise.

## Please describe how you defined "those with COVID-19 exposure" and "those without COVID-19 exposure' clearly.

We have clarified these terminologies in the revised manuscript, please. We have therefore avoided the use of the term COVID-19 exposure in the revised manuscript, please. However, what we meant by "those with COVID-19 exposure" was participants who had recovered from diagnosed COVID-19 condition. Those without COVID-19 exposure meant that such participants had never been diagnosed with the condition. As already indicated, the terms 'with past COVID-19 status' and 'without past COVID-19 status' which minimize ambiguity have rather been used in the revised manuscript, please.

From the write-up, it seems those with COVID-19 exposure included “The participants who had recovered from COVID-19 had been declared healed from the condition by a negative PCR test results coupled with a clinical determination at least eight months before recruitment and were residents of either Tamale or Cape Coast and their surrounding communities”. Is that correct?

Yes, it is correct, please.

Please state how you ascertained that clinical determination was done at least 8 months before recruitment.
Please, hospital records of the participants (those with past COVID-19 status only) were used for such determinations.

Also from the write-up, it seems those without COVID-19 exposure included “Those without a history of the COVID-19 infection originated from the same communities as their recovered counterparts but were never diagnosed of the condition”. Is that correct?

Yes, that is correct, please.

Please ensure you use the appropriate terminology. Is it exposure, infection or disease?
We were interested in disease, please. Therefore, appropriate modifications have been made in the revised document, please.

Please define your outcome variable clearly and how you measured the outcome variable. Describe how you determined the individuals with and without COVID-19 exposure.

Please, these have been clarified in the revised manuscript (pages 4-8).

**Sample size determination and participant selection:**
What was the outcome variable for this study?
How did you define P1 and P2?

Assuming the outcome variable of the study is the prevalence of insulin resistance, I would think that P1 represents the expected prevalence of insulin resistance among those with COVID-19 exposure, based on pilot studies or literature and P2 represents the expected prevalence of insulin resistance among those without COVID-19 exposure, also based on pilot studies or literature.

However, it seems P1 was taken as the estimated regional prevalence of COVID-19 and P2 as the estimated national prevalence of COVID-19. Is this correct?

What was the reason behind using the national prevalence of COVID-19 as P2?

Our study being a cross-sectional study, measured all variables at virtually the same time. In terms of our statistical analyses, insulin resistance was our outcome variable. However, in computing our sample size, we relied on the prevalence data of COVID-19 in the regional (P1) and the national (P2) levels as indicated on pages 5 and of the revised manuscript under the sub-heading "Determination of sample size". Being a comparative study, we are of the respective view that the correct formula was used, please. Using the prevalence values of COVID-19 enables us to calculate the appropriate number of respondents with past COVID-19 status which enabled us to determine the number for participants without past COVID-19 in a 1:1 ratio for the study. Thus, we took the view that since COVID-19 status is unique and can impact several variables that can be assessed in an individual depending on the objective of interest, its prevalence should be used in estimating our sample size to arrive at the appropriate numbers for our study.

Although we appreciate the view of our esteemed Reviewers in expecting insulin resistance prevalence to have been used in computing the sample size, we respectfully disagree with them in our context, please. Our esteemed Reviewers suggest that we should have conducted separate pilot studies to estimate prevalence of insulin resistance in each group of participants with and without past COVID-19 status in each study site and use the results to determine the eventual sample size. Therefore, we would have had to conduct four separate pilot studies in our case for the two metropolitan areas where our study was conducted. Respectfully, we find the suggested approach practically challenging on all fronts including costs in our context, please. Moreover, it is not the best approach for our study which seeks to contribute to the long-term health impact of the COVID-19 pandemic. Also, considering the complex nature of insulin resistance with its several surrogate markers for determination and several contributing factors, the suggested approach by our esteemed Reviewers appear impracticable, please. Additionally, judging from the findings of our work, where only two surrogate markers of insulin resistance were used with each surrogate marker giving a different prevalence figure for insulin resistance, deciding on which value to use for sample-size estimation will create another challenge for optimized use of resources. Above all, should a pilot study return such high prevalence values of insulin resistance as obtained in our setting, then almost the entire population may have to be studied, per the suggested approach to sample-size estimation by our esteemed Reviewers. However, with the current approach that relied on the prevalence values of COVID-19 at the regional and national levels for estimating the sample size, the above challenges were not encountered and our aim was achieved with the limited

resources available to us. We therefore implore our esteemed Reviewers to agree with our approach because it is appropriate for our study design and objective, under the given circumstances, please.

**Sampling procedure:**

Add a section on the sampling procedure and describe how the participants were sampled in the two teaching hospitals of the two capitals.

Please, we have addressed this concern under the sub-heading, "selection of study participants" in the revised manuscript (page 6).

**Demographic, blood pressure Anthropometry and Biochemical indices**

Please improve this section. I suggest you give it a subheading of "assessment of study variables". Describe how the study variables (outcome variable and independent variables) were assessed including how insulin resistance, COVID-19 exposure, exercise level, smoking status, alcohol status, employment status and other variables were assessed or measured. **In the data collection and measurement**, describe the data collection process including the tools used and instruments. For the instruments used, please state the country of manufacture and model number. Indicate if the data collection tools used were standardised.

Please, all these issues have been duly addressed in the revised manuscript (pages 6-8).

**<u>RESULTS</u>**

**In Table 2** description, please describe briefly the results of the different insulin resistance measures stratified by COVID-19 status instead of combining them as "the prevalence of insulin resistance ranged from 74.31 % to 89.61 % depending on the surrogate marker used". In Table 2. Please add a statistical test for the generation of p-values as a footnote to the table.

Please, this has been addressed in the revised manuscript (page 11).

**Figure 2: Prevalence of abnormal levels of blood-based indices.** Please provide a brief narrative of the prevalence of insulin resistance by the different measures in Figure 2.

Since the figure is about other indices apart from insulin resistance, the narrative has been provided for all the indices, please. The said figure is now figure 3 in the revised manuscript, please (page 12).

You can provide the prevalence of insulin resistance stratified by COVID-19 status.

This suggested has been carefully considered. However, because the other indices have not been stratified by past COVID-19 status, we have maintained it in the revised manuscript for consistency as was done in the earlier version of the manuscript, please. However, the prevalence of insulin resistance stratified by COVID-19 status has been provided on page 11 of the revised manuscript, please.

It is difficult to find the results for Figure 2 which is located at the end of the manuscript. Can you provide a narrative for Figure 2 after its subheading on page 9 if the journal guidelines indicate that the Figure should be at the end?

Please, this has been done as suggested.

Each description of the findings in the tables (1-6) should be preceded by an appropriate subheading to ensure clarity.

Please, this has been addressed in the revised manuscript (pages 9-22).

## DISCUSSION

Can you please discuss why you think participants from Tamale had significantly lower odds of insulin resistance compared to those in Cape Coast?

Please, with the use of prevalence ratio in the revised manuscript, the protective effect of Tamale residents against insulin resistance was observed in the revised manuscript.

## REFERENCES

Please ensure you include current references (10 years and below) since some of the references are very old.

We have carefully considered this suggestion by our esteemed Reviewers and we are of the respectful view that all the references including those older than 10 years are relevant as they correctly attribute the claims to the indicated authors, please. A careful look at the references older than 10 years shows that they are normally related to developed formula or concepts by the indicated authors. Since those formulas have be used in our work, it is our respectful view that those primary publications must be rightfully cited irrespective of the age, please. Assigning

citation credits to current publications that did not develop those formulas or concepts, in our respectful view, is inappropriate, please. Ultimately, we have a lot of current references below the 10-year threshold and would implore our esteemed Reviewers to kindly agree with us on this, please.

***References cited in our response***

Martinez BAF, Leotti VB, Silva GS, Nunes LN, Machado G, Corbellini LG Odds Ratio or Prevalence Ratio? An Overview of Reported Statistical Methods and Appropriateness of Interpretations in Cross-sectional Studies with Dichotomous Outcomes in Veterinary Medicine. *Front Vet Sci* 2017: 4(193). doi: 10.3389/fvets.2017.00193

Tamhane AR, Westfall AO, Burkholder GA, Cutter GR. Prevalence odds ratio versus prevalence ratio: choice comes with consequences. Stat Med. 2016 35(30):5730-5735. doi: 10.1002/sim.7059. Erratum in: Stat Med. 2017;36(23):3760. doi: 10.1002/sim.7375.
