# Supplementary material for: Prevalence and determinants of insulin resistance in recovered COVID-19 and uninfected residents of two regional capitals in Ghana: An observational study
Source: PLOS Glob Public Health. 2025 Apr 24;5(4):e0004506. doi: 10.1371/journal.pgph.0004506 (PMC12021235; doi:10.1371/journal.pgph.0004506)
Supplement: Attachment — Submitted filename: RESPONSE TO REVIEWERS COMMENTS.docx [file pgph.0004506.s002.docx]

Department of Medical Biochemistry

School of Medical Sciences

College of Health and Allied Sciences

University of Cape Coast

17^th^ June, 2024

The Editor

PLOS Global Public Health

Dear Sir,

**SUBMISSION OF REVISED MANUSCRIPT FOR CONSIDERATION**

In response to your decision for us to undertake a MINOR review of our manuscript titled
“*Prevalence and Determinants of Insulin Resistance in Residents of Two Regional Capitals in Ghana: An Observational Study*” with reference number PGPH-D-24-00529, we write to submit the revised manuscript, for your further action, please.

All the issues raised by our esteemed Reviewer have been carefully addressed, to the best of our ability. In few instances that we disagreed respectfully with our esteemed Reviewer, we have provided appropriate scientific explanations, which, we believe, will be found acceptable for further action.

The various changes made in the revised manuscript have been highlighted with yellow background for ease of reference, please. Additionally, the specific locations of the changes in the revised manuscript have been indicated in our detailed response to each of the comments made by our esteemed Reviewer, as attached, please.

We are, therefore, optimistic that our esteemed Reviewer and Editor would find our revised manuscript acceptable, without any further revision.

We are very much grateful to your team for the unique contributions that have greatly improved manuscript.

We are looking forward to your positive feedback in due course.

Yours faithfully,

Samuel Acquah (PhD)

**CORRESPONDING AUTHOR**

**RESPONSE TO REVIEWERS COMMENTS**

Please, the boldened information in Arial font size 14 are the exact comments made by the esteemed Reviewer. We have presented our response in font size 12 of Times New Roman in all cases, please.

**Reviewer #1: While the article provides valuable insights into the potential long-term health impacts of COVID-19, particularly regarding the development of type 2 diabetes mellitus (T2DM) and insulin resistance in the sub-Saharan African population, it has several limitations:**
1. **The use of convenience sampling and recruitment from only two healthcare facilities could introduce selection bias, as participants may not be representative of the general population. This could limit the external validity of the study.**

This concern by our esteemed Reviewer is well noted. However, in the context of our work, the concern is non-existent because we did not use convenience sampling in our recruitment, please. Rather, we used the simple random sampling technique for recruiting participants for the study. We made this point in our earlier submission and we have duly maintained it in the revised version on page 5 under the sub-heading, *Determination of sample size and selection of participants*.

The two health facilities used are teaching hospitals which serve as referral hospitals for various health facilities within the regions and nearby regions without such facilities. Therefore, the participants were representative of the two regions where the study was conducted, please. Selection bias due to non-representativeness of the general population is not likely in our case. To this end, the fear of limitation of the study’s external validity does not arise in our case, please.

2. **While the study aims to identify determinants of insulin resistance in individuals with a history of COVID-19 compared to those without, it does not explore potential mechanisms underlying the relationship between COVID-19 and insulin resistance.**

Although this observation by our esteemed Reviewer is well noted, we respectfully disagree, please. Some of the mechanisms underlying the relationship between insulin resistance and COVID-19 were indicated in the introductory and discussion sections of our manuscript (pages 3, 4 and 21, please). The known mechanisms involve the integrated stress response (ISR) system (Santos et al., 2021), inflammation, insulin synthesis and clearance (Chen et al., 2021), obesity (Cohen & LeRoith, 2012), etc, which have been duly alluded to in our current work. Indeed, we measured indices of obesity (BMI, WHR, WC), inflammation (CRP), lipid metabolism (lipid profile-LDL, HDL, total cholesterol, etc) and beta-cell function (HOMA-B). These indices are proxy to some of the mechanisms, please. In addition, we considered other demographic and lifestyle factors such as age, gender, alcohol intake, smoking status, formal education, and others, which are known to be important to the concept of insulin resistance. We employed the logistic regression analyses to explore the mechanisms, if any, underlying insulin resistance in our setting. In our multiple regression analyses, we controlled for several factors as possible in our attempt to identify any of the measured indices (predictors) as a statistically significant contributor to the observed insulin resistance. To this end, we believe that we actually explored the mechanisms underlying insulin resistance in our sample during the data analysis phase of our work.

In terms of our findings, we did not find any parameter apart from being resident in Tamale as a predictor of insulin resistance in the COVID-19 exposed group of our respondents. Since our currently available data does not single out any specific index, for this group of respondents, we situated our discussion in the current literature available on the subject matter. In the COVID-19 unexposed participants, tertiary education, age 50-59 years and high levels of fasting plasma glucose and insulin and being overweight increased the odds of developing insulin resistance although high beta-cell function and residing in Tamale were protective against development of the condition. Thus, in this group of respondents, the associated direct biological mechanism underlying the observed insulin resistance could be ascribed to weight gain.

Therefore, in terms of exploration of the underlying mechanism, we did explore through our data analytic approach but our results did not point to anything substantial apart from the town of resident, as far as the COVID-19 respondents/participants are concerned. For this reason we have called for further studies as expected to unravel the specific factor(s), if any, which are driving the development of insulin resistance in COVID-19 respondents in our context. For instance, Chen et al. (2021) speculated reduced insulin clearance as a probable mechanism but we did not measure insulin clearance in our work.

It is our respectful view that the absence of a clearly defined mechanism (predictor) of insulin resistance in our COVID-19 exposed group apart from the town of residence, should rather call for more research work in the area, which we have done. To this end, we are convinced that the lack of additional predictors of insulin resistance apart from residential location is not detrimental enough to negate the other findings, please. We, therefore, believe that, in the current circumstances, our results are worth sharing with the scientific community and hope our esteemed Reviewer agrees to us, please.

3. **The study's use of fasting samples may not capture postprandial changes in insulin sensitivity, which could affect the interpretation of results.**

The use of fasting samples is not new in this area of research although it is acknowledged that fasting samples are subject to fluctuations. However, our interpretations were based on the fasting samples and were never projected as postprandial ones in our write-up, please. Indeed, the index of insulin resistance (homeostatic model assessment of insulin resistance, HOMA-IR and the triglyceride-glucose index, TyG, used in our setting, were developed under fasting conditions (Matthews et al., 1985; Simental-Mendía et al., 2008) and are suitable for fasting samples. Thus, the use of these indices for assessment of insulin resistance has always been under fasting conditions as we did in line with several published works (Guerrero-Romero et al., 2010; Luo et al., 2022). Even in terms of lipid profile, the ATPIII-NCEP recommendations were based on fasting samples, making our fasting-based interpretation appropriate for our work.

However, postprandial assessment of insulin sensitivity is normally done with oral glucose tolerance test (OGTT) or the hyperglycaemia-euglycaemia clamp technique (DeFronzo et al., 1979), which is the gold standard. We have duly acknowledged the absence of postprandial indices as part of the limitations of the current study. To this end, we are convinced that our results have been interpreted correctly in accordance with the fasting conditions and hope that our esteemed Reviewer agrees with us. Therefore, there should be no problem with interpretation of our current results, please.

4. **The study lacks detailed information on lifestyle factors such as diet and exercise, which are known to influence insulin resistance.**

This view was acknowledged in our earlier submission as part of the limitations of the study including the study design and fasting parameters without postprandial ones (please, it can be found in the last paragraph of the discussion section on page 22 of revised manuscript). It is our respectful view that the various indices measured such as BMI, WHR, WC, CRP, LDL, HDL, total cholesterol, etc, which influence insulin resistance are also affected by the lifestyle factors. Therefore, the lifestyle factors such diet and exercise which detailed could not be examined in the current study, are partly reflected in our measured indices. Indeed, examining detailed information on diet and exercise would have enriched our current data and make it easier to explain our findings. However, in the absence of such detailed dietary and exercise information, we believe that our findings are still worth sharing with the scientific community, please. In subsequent studies, these factors can be explored, please.

5. **The study's reliance on cross-sectional data limits its ability to track changes in insulin resistance over time. Longitudinal studies would provide more robust evidence of the long-term effects of COVID-19 on insulin sensitivity.**

As already indicated, the cross-sectional nature of our study does not allow for a causal relationship to be studied and this fact was acknowledged as a limitation, please. However, cross-sectional studies like ours are necessary to provide the necessary proof-of-concept information that forms the basis for longitudinal studies to gather the requisite more robust evidence for long-term decision-making. It is in this context that the current study should be considered, please. Therefore, we are convinced that under the circumstance, the cross-sectional study design is appropriate since it has provided some indications of associations that must be further studied.

Since we did not set out to investigate the changes in insulin resistance over time, which is only feasible in longitudinal settings, we believe the suggestion could be considered in future studies as part of the recommended further studies, please. In such studies, the specific detailed lifestyle factors such as exercise and diet may be incorporated to identify the most appropriate physical activity and diet necessary for reduced insulin resistance in our context. In the absence of such studies in our context, we believe that the evidence provided by our cross-sectional study is in line with our study objective and worth sharing with the scientific community, please.

6. **While the study conducts statistical analyses to assess associations between variables, it does not explicitly address the possibility of type I or type II errors, which could affect the reliability of study findings. Additionally, the interpretation of statistical significance should consider clinical relevance and effect sizes.**

It has been duly acknowledged by several studies (Banerjee et al., 2009; Rothman, 2010; Smith, 2012; Kaur & Stoltzfus, 2017) that types I and II errors cannot be completely avoided or erased in any given study irrespective of the design but can only be minimized. In our case, we minimized type I error by our increased sample size from 424 to 510 participants. In terms of type II error, we used 80% power in our sample size estimation. Therefore, we addressed these errors even at the conception stage of our work, in line with published literature (Taylor & Muncer, 2000; Bezeau & Grave, 2001; Banerjee et al., 2009; Rothman, 2010; Smith, 2012; Gaskin & Happell, 2014; Kaur & Stoltzfus, 2017). To this end, our study is powered enough to reduce the risk of type II error as well as the type I error.

On the issue of clinical relevance, we are convinced that we have addressed it already in our work. Although our study involved participants who were not sick at the time of the study, a number of the indices used have proven clinical relevance. Therefore, clinical relevance is inherent in our study, please.

On the effect size (Chinn, 2000; Sánchez-Meca et al., 2003), we acknowledge that we did not include it in our earlier submission. Therefore, we have included it in the revised manuscript for tables 2-6 (pages 8-17), to provide the needed further information requested. Although it did not affect the major findings and discussions, we have modified the results section to include the strength of the associations found as indicated by the effect size, in accordance with the recommendation, please. From pages 8-16 of the revised manuscript, such minor modifications can be found, please.

**REFERENCES**

Banerjee A, Chitnis UB, Jadhav SL, Bhawalkar JS, Chaudhury S. Hypothesis testing, type I and type II errors. Ind Psychiatry J. 2009;18(2):127-31. doi: 10.4103/0972-6748.62274.

Bezeau S, Graves R. Statistical power and effect sizes of clinical neuropsychology research. J Clin Exp Neuropsychol. 2001;23(3):399-406.

Chen M, Zhu B, Chen D, Hu X, Xu X, Shen WJ, et al. COVID-19 may increase the risk of insulin resistance in adult patients without diabetes: a 6-month prospective study. Endocr Pract. 2021;27(8): 834-841. [doi: 10.1016/j.eprac.2021.04.004](https://doi.org/10.1016/j.eprac.2021.04.004).

Chinn S. A simple method for converting an odds ratio to effect size for use in meta-analysis. Stat Med. 2000;19(22): 3127–31. [doi:10.1002/1097-0258(20001130)19:22<3127::aid-sim784>3.0.co;2-m](https://doi.org/10.1002/1097-0258(20001130)19:22%3c3127::aid-sim784%3e3.0.co;2-m)

Cohen DH, LeRoith D. Obesity, type 2 diabetes, and cancer: the insulin and IGF connection. Endocr-Relat. Cancer 2012;19(5): F27–F45. [doi: 10.1530/ERC-11-0374](https://doi.org/10.1530/ERC-11-0374).

DeFronzo RA, Tobin JD, Andres R. Glucose clamp technique: a method for quantifying insulin secretion and resistance. Am J Physiol. 1979;237(3): E214-223. doi: 10.1152/ajpendo.1979.237.3.E214.

Expert Panel on Detection, Evaluation, and Treatment of High Blood Cholesterol in Adults. Executive summary of the third report of the National Cholesterol Education Program (NCEP) Expert Panel on Detection, Evaluation, and Treatment of High Blood Cholesterol in Adults (Adult Treatment Panel III). JAMA. 2001;285(19): 2486-2497. doi: 10.1001/jama.285.19.2486.

Gaskin CJ, Happell B. Power, effects, confidence, and significance: an investigation of statistical practices in nursing research. Int J Nurs Stud. 2014;51(5):795-806.

Guerrero-Romero F, Simental-Mendía LE, González-Ortiz M, Martínez-Abundis E, Ramos-Zavala MG, Hernández-González SO, et al. The product of triglycerides and glucose, a simple measure of insulin sensitivity. Comparison with the euglycemic-hyperinsulinemic clamp. J Clin Endocrinol Metab. 2010;95(7):3347-3351. doi: 10.1210/jc.2010-0288.

Kaur P, Stoltzfus, J^,^. Type I, II, and III statistical errors: A brief overview. International Journal of Acad Med. 2017;3(2):268-70. doi: 10.4103/IJAM.IJAM_92_17

Luo P, Cao Y, Li P, Li W, Song Z, Fu Z, et al. TyG index performs better than HOMA-IR in Chinese type 2 diabetes mellitus with a BMI < 35 kg/m^2^: a hyperglycemic clamp validated study. Medicina (Kaunas). 2022;58(7):876. doi: 10.3390/medicina58070876.

Matthews DR, Hosker JP, Rudenski AS, Naylor BA, Treacher DF, Turner RC. Homeostasis model assessment: insulin resistance and 𝛽-cell function from fasting plasma glucose and insulin concentrations in man. Diabetologia 1985; 28(7): 412–419. doi: 10.1007/BF00280883.

Rothman KJ. Curbing type I and type II errors. Eur J Epidemiol. 2010;25(4):223-4. doi: 10.1007/s10654-010-9437-5.

Sánchez-Meca, Julio, Fulgencio Marı́n-Martı́nez, and Salvador Chacón-Moscoso. Effect-Size Indices for Dichotomized Outcomes in Meta-Analysis. Psychol Methods.2003;8(4):448.

Santos A, Magro DO, Evangelista-Poderoso R, Saad MJA. Diabetes, obesity, and insulin resistance in COVID-19: molecular interrelationship and therapeutic implications. Diabetol Metab Syndr. 2021 Mar 1;13(1):23. doi: 10.1186/s13098-021-00639-2.

Simental-Mendía LE, Rodríguez-Morán M, Guerrero-Romero F. The product of fasting glucose and triglycerides as surrogate for identifying insulin resistance in apparently healthy subjects. Metab Syndr Relat Disord. 2008;6(4): 299-304. doi: 10.1089/met.2008.0034.

Smith CJ. Type I and Type II errors: what are they and why do they matter? Phlebology. 2012;27(4):199-200. doi:[10.1258/phleb.2012.012j04](https://doi.org/10.1258/phleb.2012.012j04)

Taylor S, Muncer S. Redressing the power and effect of significance. A new approach to an old problem: teaching statistics to nursing students. Nurse Educ Today, 2000;20(5):358–64. doi: 10.1054/nedt.2000.0429
