# Supplementary material for: Prevalence and determinants of insulin resistance in recovered COVID-19 and uninfected residents of two regional capitals in Ghana: An observational study
Source: PLOS Glob Public Health. 2025 Apr 24;5(4):e0004506. doi: 10.1371/journal.pgph.0004506 (PMC12021235; doi:10.1371/journal.pgph.0004506)
Supplement: Attachment — Submitted filename: RESPONSE TO REVIEWERS COMMENTS--PLOS ONE-13-02-25.docx [file pgph.0004506.s005.docx]

**RESPONSE TO REVIEWERS COMMENTS**

Please, the boldened information in Arial font size 14 are the exact comments made by the esteemed Reviewer. We have presented our response in font size 12 of Times New Roman in all cases, please.

**Several comments should be addressed.**

All the comments raised have been duly addressed, please.

**A general comment if that the results section is a bit confusing since several ways of ins resistance are considered. Results should be clear-cut (maybe choose one model).**

We have carefully considered this comment and responded appropriately by introducing appropriate modifications for improved clarity (pages 4, 7, 20 and 23 of the revised manuscript), please. We used two surrogate markers (HOMA-IR and TyG) for the assessment of insulin resistance in our context. Indeed, several other surrogate markers have been developed for the assessment of insulin resistance (Singh & Saxena, 2010; Najmunnissa et al., 2024; Mirjalili et al., 2024), due to various challenges associated with the gold-standard, the euglycemic-hyperinsulinemic clamp technique (DeFronzo et al., 1979). As expected, each of the surrogate markers has its advantages and disadvantages. Therefore, the use of more than one surrogate marker to assess insulin resistance in a given study in our view should be advantageous because of the complexity of the phenomenon and its relationship with several other factors. In our case, we used HOMA-IR and TyG as the indices for insulin resistance assessment. The use of the two surrogate markers allowed us to identify a wider range of factors that influence insulin resistance in our sample. The use of only one surrogate marker would not be able to identify all the factors/predictors, please.

For instance, HOMA-IR could not be predicted by the indices of anthropometry, lipid metabolism and inflammation used in the current study. However, the use of TyG index allowed an anthropometric index of obesity (BMI) to emerge as a predictor for insulin resistance in the entire sample irrespective of COVID-19 background, although a sub-group analysis demonstrated overweight as the culprit in respondents without a history of COVID-19 infection. Additionally, glucose level and beta-cell function (HOMA-B) were identified to be predictors of TyG-determined insulin resistance. This means that using only HOMA-IR in our context would have missed all these important contributors identified by the TyG index. HOMA-IR, on the other hand could be predicted by age, education, location (Tamale or Cape Coast), and insulin levels. One can clearly observe that the two surrogate markers used in our study complemented each other to give a broad range of factors that influence insulin resistance, per available literature (DeFronzo et al., 1979; Singh & Saxena, 2010; Li et al., 2022; Luo et al., 2022; Najmunnissa et al., 2024; Mirjalili et al., 2024; Arneth, 2024). Therefore, we are of the respectful view that our use of two surrogate markers instead of just one of them is more appropriate for our study in achieving our objective, please, and implore our esteemed Reviewers to agree with us in this regard.

Therefore, to eliminate the seeming confusion, we have modified relevant sections of the manuscript to improve clarity, please (pages 4, 7, 20 and 23 of the revised manuscript).

**The second paragraph of the introduction should be shorter (by half).
Insulin resistance, which signifies reduced sensitivity of cells to insulin cues, is a cardinal risk factor for the development of T2DM. Insulin resistance is influenced by several factors including infection, obesity, diet and other lifestyle factors. These sentences can be deleted.**

We have duly shortened this paragraph by deleting the suggested sentence, please (page 3 of revised manuscript). An additional sentence has also been deleted to meet the expectations of our esteemed Reviewer, please.

**Paragraph: Demographic, Blood Pressure, Anthropometry and Biochemical Indices
An experience nurse measured. Please change to “An experienced nurse measured”**

This has been done, please (page 6 of the revised manuscript).

**Table 1: It is not very clear which comparisons are made for the p-values displayed. Please provide an explanation under the Table.**

Please, this has been done (pages 6 and 7 of the revised manuscript).

**Table 2 and throughout the text: HDL, LDL and VLDL should be : HDL-cholesterol, LDL-cholesterol and VLDL- cholesterol.**

We have duly defined HDL (high-density lipoprotein cholesterol), LDL (low-density lipoprotein cholesterol) and VLDL (very low-density lipoprotein cholesterol) on pages 6 and 9 of the revised manuscript, please. Therefore, the subsequent use of the abbreviations alone in the text is acceptable in our respectful view, please. In Table 2 and all other sections (tables 3-6) where the abbreviations, HDL, LDL and VLDL have been used, the full meaning of each of the abbreviations has been provided beneath the table, please. Because of the definitions provided, we are of the respectful view that we do not need to attach cholesterol to each of the abbreviations again, please.

**It is not clear of one or several logistic regression models were applied. It is suggested to provide data on simple and more complex models. Moreover, some indices may not be used together in the same model (for example waist circ and WHR). It is strongly suggested to apply models with multiple explanatory variables at once to see if the effects of each variable are lessened in the presence of other variables.**

We have carefully considered this observation and associated suggestion by our esteemed Reviewer. We built two main regression models, the simple and the complex models, for the entire sample as well as the sub-groupings, for each of the surrogate markers of insulin resistance (HOMA-IR and TyG). We are of the respectful view that the exact expected information is what was provided in the earlier version of the manuscript, which have been maintained in the revised version, please.

We built our regression models (Pourhoseingholi et al., 2012; Sperandei, 2014; Szumilas, 2015; Kahlert et al., 2017), using the manual procedure. In our manuscript, the regression results are in tables 3-6. Each table contains the needed information on the simple model as well as the complex model. In Table 3 and Table 4, the first parts of the tables contain the results for the simple models whose associations were determined by the crude odds ratio (COR) with the second parts of the tables providing the needed information for the complex models being requested for by our esteemed Reviewer. The COR was obtained by looking at one variable at a time. The complex models adjusted for confounders and the odds ratio is designated as adjusted odds ratio (AOR) in our case. The adjusted model with its associated AOR provides a more accurate outcome of the effect of each variable in the presence of other variables on the outcome variable (HOMA-IR or TyG). In our case, the outcome variable is insulin resistance, which was measured by HOMA-IR and TyG.

In tables 5 and 6, similar analyses were carried out except that the respondents were divided into COVID-19 exposed and unexposed groups. In these tables for each study group, the first part designated the associations as COR (for the simple model) with the second part being the AOR (for the complex model). As expected, the simple model looked at the effect of one variable at a time (COR) on the outcome variable as the complex model examined effect of each variable in the presence of the others (AOR) on the outcome variable (HOMA-IR or TyG).

Therefore, each of the tables, Table 3-6, has the results for the simple and the complex models being referred to by our esteemed Reviewer, please. It is worthy of note that our use of both HOMA-IR and TyG index to assess insulin resistance in our context allowed for the identification of more predictors of insulin resistance in our sample which would have been missed if any one of them had been used alone, please.

Regarding the use of both waist circumference (WC) and waist-to-hip ratio (WHR) in our regression models, we are of the respectful view that there is nothing wrong with it, please. We acknowledge that both WC and WHR are measures of central obesity (Ahmad et al, 2016; Lam et al., 2015). However, none of them is able to perfectly identify all individuals that truly have the phenomenon of central obesity in a given population. In addition, the strengths of their associations differ with studies (Ahmad et al, 2016; Lam et al., 2015; Ozdemir, 2023). For example, individuals with relatively wide or narrow hips may be easily misclassified by WHR but WC may detect them appropriately for classification. On the other hand, WC may miss some individuals which may be detected by WHR. Their use in our regression models is therefore appropriate as they are independent risk factors for some obesity-related health conditions (Ahmad et al, 2016; Lam et al., 2015), please. Thus, the use of both WHR and WC in work ensured that we did not miss anyone who had central obesity in our context, please.

**Table 5: ; SE: Effect size do you mean: ES: Effect size**

Please, thus has been done (page 17, table 5 and page 19 table 6 of the revised manuscript).

**It would be also interesting to have data on COVID vaccination.**

As part of our inclusion criteria, each participant needed to have been vaccinated against COVID-19 before being enrolled for the study (page 5 of the revised manuscript, please). Therefore, all participants/respondents had been vaccinated against the COVID-19 virus (page 5 of revised manuscript). However, detailed data on the specific vaccines administered could not be obtained, please. This notwithstanding, we are of the respectful view that the absence of the detailed data on the specifics of the vaccine(s) does not invalidate our findings, please.

**The logistic regression models of both ways estimation of insulin resistance is confusing. You can choose one way and the other one can be moved to supplementary data.**

We have carefully considered this comment by our esteemed Reviewer and our respectful view is that all the results generated in our manuscript are necessary for effective comprehension of our work, please. Moreover, the number of tables and figures in our manuscript falls within the acceptable range of the journal, please. In this regard, we would like to implore our esteemed Reviewer to agree with us, please. Each table contains relevant information that enable us to achieve our set objectives, please.

The two surrogate markers (HOMA-IR and TyG) used to measure insulin resistance in our context were complementary to each other and helped us to identify a wider range of factors that influence insulin resistance according to available literature (DeFronzo et al., 1979; Singh & Saxena, 2010; Li et al., 2022; Najmunnissa et al., 2024; Mirjalili et al., 2024; Arneth, 2024), please. Moreover, the use of more than one surrogate marker in assessing insulin resistance in a given context is prevalent in literature (Wang et al., 2021; Luo et al., 2022; Zeng et al., 2022; Dundar et al., 2023).

However, we have modified our write-up (pages 4, 7, 20 and 23 of the revised manuscript) to improve the clarity to eliminate the perceived confusion, please. It is our hope that with these modifications and explanations, our esteemed Reviewer will find the revised manuscript to be clearer and more acceptable than the previous version, please.

**How do authors explain that living in Tamala was a protective factor against insulin resistance?**

This observation could be due to other environmental factors including physical activity, dietary and nutritional issues (page 22 of revised manuscript, please). However, we could not collect detailed information on these factors and have duly acknowledged it as part of the limitations that need to be addressed in future studies, please (page 22 of revised manuscript).

**Do we have any data regarding supplements intake? It is known that nutrition plays a role in COVID-19 by modulating several pathways of inflammation including PAF (**[**https://www.ncbi.nlm.nih.gov/pmc/articles/PMC7911163/**](https://www.ncbi.nlm.nih.gov/pmc/articles/PMC7911163/)**).**

We have duly acknowledged this reference in our discussion (page 22 of the revised manuscript), please. However, we could not collect detailed information on dietary, nutritional and other lifestyle factors. This has been duly acknowledge as part of the limitations of the current study, please.

**REFERENCES**

Ahmad N,  Adam SIM,  Nawi AM,  Hassan MR,  Ghazi HF. Abdominal obesity indicators: Waist circumference or waist-to-hip ratio in Malaysian adults population. Int J Prev Med. 2016;7:82. doi: [10.4103/2008-7802.183654](https://doi.org/10.4103%2F2008-7802.183654).

Arneth B. Mechanisms of Insulin Resistance in Patients with Obesity. Endocrines. 2024; 5(2):153-165. [doi.10.3390/endocrines5020011](https://doi.org/10.3390/endocrines5020011).

DeFronzo RA, Tobin JD, Andres R. Glucose clamp technique: a method for quantifying insulin secretion and resistance. Am J Physiol. 1979;237(3): E214-223. doi: 10.1152/ajpendo.1979.237.3.E214.

Dundar C, Terzi O, Arslan HN. Comparison of the ability of HOMA-IR, VAI, and TyG indexes to predict metabolic syndrome in children with obesity: a cross-sectional study. BMC Pediatr. 2023;23(1):74. doi: 10.1186/s12887-023-03892-8.

Kahlert J, Gribsholt SB, Gammelager H, Dekkers OM, Luta G. Control of confounding in the analysis phase – an overview for clinicians. Clin Epidemiol. 2017;9:195-204. [doi.10.2147/CLEP.S129886](https://doi.org/10.2147/CLEP.S129886).

Lam BC, Koh GC, Chen C, Wong MT, Fallows SJ. Comparison of body mass index (BMI), body adiposity index (BAI), waist circumference (WC), waist-to-hip ratio (WHR) and waist-to-height ratio (WHtR) as predictors of cardiovascular disease risk factors in an adult population in Singapore. PLoS One. 2015;10(4):e0122985. doi: 10.1371/journal.pone.0122985.

Li, M., Chi, X., Wang, Y. et al. Trends in insulin resistance: insights into mechanisms and therapeutic strategy. Sig Transduct Target Ther **7**, 216 (2022). doi.10.1038/s41392-022-01073-0.

Luo P, Cao Y, Li P, Li W, Song Z, Fu Z, Zhou H, Yi X, Zhu L, Zhu S. TyG index performs better than HOMA-IR in Chinese type 2 diabetes mellitus with a BMI < 35 kg/m2: A hyperglycemic clamp validated study. Medicina (Kaunas). 2022;58(7):876. doi: 10.3390/medicina58070876.

Mirjalili SR, Soltani S, Meybodi ZH et al. Which surrogate insulin resistance indices best predict coronary artery disease? A machine learning approach. Cardiovasc Diabetol. 2024;23(214). doi.10.1186/s12933-024-02306-y.

Najmunnissa, Guruswamy KM, Gowda J, Nataraj S M. Unveiling the Significance of Surrogate Markers of Insulin Resistance in Metabolic Health Assessment. Indian J Med Biochem. 2024;28(2):45–53.

Ozdemir O. Evaluation of waist circumference, waist-hip ratio and waist-length ratio in insulin resistance: which is a powerful predictor? Ann of Med Res 2023;30(5):533–537.

Pourhoseingholi MA, Baghestani AR, Vahedi M. How to control confounding effects by statistical analysis. Gastroenterol Hepatol Bed Bench. 2012;5(2):79-83.

Singh B, Saxena A. Surrogate markers of insulin resistance: A review. World J Diabetes. 2010;1(2):36-47. doi: 10.4239/wjd.v1.i2.36.

Sperandei S. Understanding logistic regression analysis. Biochem Med (Zagreb). 2014;24(1):12-18. doi: 10.11613/BM.2014.003.

Szumilas M. Explaining odds ratios. J Can Acad Child Adolesc Psychiatry. 2010;19(3):227-9. Erratum in: J Can Acad Child Adolesc Psychiatry. 2015;24(1):58.

Wang, S., Shi, J., Peng, Y. et al. Stronger association of triglyceride glucose index than the HOMA-IR with arterial stiffness in patients with type 2 diabetes: a real-world single-centre study. Cardiovasc Diabetol. 2021;20:82. doi.10.1186/s12933-021-01274-x.

Zeng X, Han D, Zhou H, Xue Y, Wang X, Zhan Q, Bai Y, Huang X, Zeng Q, Zhang H, Ma Z, Ren H, Xu D. Triglyceride-glucose index and homeostasis model assessment-insulin resistance in young adulthood and risk of incident congestive heart failure in midlife: The coronary artery risk development in young adults Study. Front Cardiovasc Med. 2022;9:944258. doi: 10.3389/fcvm.2022.944258.
