# Supplementary material for: Prevalence and determinants of insulin resistance in recovered COVID-19 and uninfected residents of two regional capitals in Ghana: An observational study
Source: PLOS Glob Public Health. 2025 Apr 24;5(4):e0004506. doi: 10.1371/journal.pgph.0004506 (PMC12021235; doi:10.1371/journal.pgph.0004506)
Supplement: Attachment — Submitted filename: Reviewer Comments PGPH-D-24-00529 DL.pdf [file pgph.0004506.s004.pdf]

PLOS Global Public Health

Prevalence and Determinants of Insulin Resistance in Residents of Two Regional Capitals in Ghana: An Observational Study

Authors

Ansumana Sandy Bockarie, Leonard Derkyi-Kwarteng, Jeffrey Amankona Obeng, Richard Kujo Adatsi, Ebenezer Aniakwaa-Bonsu, Charles Apprey, Jerry Ampofo Asiamah, Samuel Acquah

Reviewer Comments:

I thank the authors for this important paper on the "Prevalence and determinants of insulin resistance in residents of two regional capitals in Ghana: An observational study".
I hope the comments below will enhance the quality of the manuscript.

General comments.
1. It is a fairly good write-up that can be improved.
2. There are some typos and grammatical errors that could be corrected.
3. Please include line numbers in the manuscript for ease of reference.

Title

The title is "Prevalence and determinants of Insulin resistance in residents of two regional capitals in Ghana: An observational study".

I believe the title can be improved to reflect the study's aim of comparing insulin resistance prevalence and determinants among individuals exposed to Covid-19 and those not exposed. Since the focus is on analysing differences based on **COVID-19 exposure**, it would be good to reflect this aspect in the title.

The title seems to imply that the study was community-based and conducted in two regional capitals in Ghana. Is this correct? However, the methods suggest that the study was hospital-based. Could you please clarify this discrepancy and ensure that the correct assertion is reflected consistently throughout the manuscript?

ABSTRACT
In the **background,** consider replacing 'devastative' with "devastating".
What is the difference between COVID-19 exposure, COVID-19 infection and COVID-19 disease?
The last statement in the abstract background states "The current study sought to compare the factors that determine insulin resistance in Ghanaians with and **without COVID-19 exposure**,

resident in the Cape Coast and Tamale metropolitan areas", while the last statement in the introduction section of the main manuscript states "the current study aims to identify determinants of insulin resistance in a Ghanaian population that has recovered completely from clinically mild cases of COVID-19 and their counterparts that never had a clinical history of the condition in two tertiary facilities".

The authors did not clearly state the eligibility criteria in the methods section of the main manuscript. It appears that the term "**without COVID-19 exposure"** as stated in the abstract, is described in the methods section as "those **without a history of the COVID-19 infection who** originated from the same communities as their recovered counterparts but were **never diagnosed with the condition**". However, this definition of "without a history of the COVID-19 infection" inherently includes individuals who may have been **potentially exposed** but undiagnosed or **exposed** but asymptomatic. How did you address the potential issue of misclassification in this context?
Did the authors also ascertain that this category of "without COVID-19 exposure" had not been in contact with either a confirmed or suspected or case exposed to a high-risk environment? If they did, please describe how this was ascertained. This is because in their criteria they considered only "history of COVID-19 infection" and "never diagnosed" as highlighted above. Please provide a clarification of these issues.

In the **methods**, please provide a brief description of the data collection methods and indicate the outcome variable and the independent variables. Additionally, briefly describe the conduct of data analysis to assess the prevalence and determinants, including the measures of association and level of statistical significance.

In the **results**, please provide the names of the specific surrogate markers used along with their related prevalence and confidence intervals.
Authors stated that "Prevalence of insulin resistance ranging from **74.31%** to **89.61%** depending on the surrogate marker used coupled with low (**31.18%)** and high (**19.41%)** beta-cell function, were observed". I suggest the authors present the results of the specific surrogate markers rather than combining them in a range as above.
Please, as per the title, provide results on the prevalence of insulin resistance among those exposed to COVID-19 and those not exposed to COVID-19.

I think it would be more informative to break down the dyslipidemias individually, rather than combining the percentages into a range (8.31 % - 44.71%).
Please present the measures of association with their confidence intervals. If the outcome of interest is above 10%, is it appropriate to use Odds Ratios as a measure of association?
Conclusion: Please review the conclusion.
Is there evidence from this study that "educating the adult populace <u>to adopt lifestyle measures</u> can reduce insulin resistance"?

<u>INTRODUCTION</u>
In the last paragraph, on page 4, the authors stated the aim as "to identify determinants of insulin resistance using two surrogate markers in a Ghanaian population that had recovered completely from clinically mild cases of COVID-19 and their counterparts that never had a clinical history of the condition in two tertiary facilities".
I suggest authors maintain consistency in the use of terms, particularly the exposure variable related to COVID-19 status. The aim stated in the introduction differs from the one provided

in the abstract. I think not having a clinical history of COVID-19 is not the same as not being exposed to Covid 19, as I indicated in the abstract comments. Please consider and clarify.

METHODS

Study site: What was the rationale for choosing Cape Coast (Teaching Hospital (CCTH) and Tamale metropolis (Tamale Teaching Hospital (TTH), sites which have "differing geography, climate, ethnicity and dietary norms" as study sites?

Please add a location map of the study sites.

Can you clearly state that the study was either a community-based study or a facility-based study?

Describe the eligibility criteria and clearly outline the inclusion and exclusion criteria.

Why did you select your participants to be above 20 years old and not adults 18 years and above?

You stated that the study population comprised participants "...... without underlying health conditions at the time of recruitment". Please describe how the screening was done to ensure that you met this criterion.

Please describe how you defined "those with COVID-19 exposure" and "those without COVID-19 exposure' clearly.

From the write-up, it seems those with COVID-19 exposure included "The participants who had recovered from COVID-19 had been declared healed from the condition by a negative PCR test results coupled with a clinical determination at least eight months before recruitment and were residents of either Tamale or Cape Coast and their surrounding communities". Is that correct?

Please state how you ascertained that clinical determination was done at least 8 months before recruitment.

Also from the write-up, it seems those without COVID-19 exposure included "Those without a history of the COVID-19 infection originated from the same communities as their recovered counterparts but were never diagnosed of the condition". Is that correct?

Please ensure you use the appropriate terminology. Is it exposure, infection or disease?

Please define your outcome variable clearly and how you measured the outcome variable.

Describe how you determined the individuals with and without COVID-19 exposure.

Sample size determination and participant selection:

What was the outcome variable for this study?

How did you define P1 and P2?

Assuming the outcome variable of the study is the prevalence of insulin resistance, I would think that P1 represents the expected prevalence of insulin resistance among those with COVID-19 exposure, based on pilot studies or literature and P2 represents the expected prevalence of insulin resistance among those without COVID-19 exposure, also based on pilot studies or literature.

However, it seems P1 was taken as the estimated regional prevalence of COVID-19 and P2 as the estimated national prevalence of COVID-19. Is this correct?

What was the reason behind using the national prevalence of COVID-19 as P2?

Sampling procedure:

Add a section on the sampling procedure and describe how the participants were sampled in the two teaching hospitals of the two capitals.

Demographic, blood pressure Anthropometry and Biochemical indices

Please improve this section. I suggest you give it a subheading of "assessment of study variables". Describe how the study variables (outcome variable and independent variables)

were assessed including how insulin resistance, COVID-19 exposure, exercise level, smoking status, alcohol status, employment status and other variables were assessed or measured.
In the data collection and measurement, describe the data collection process including the tools used and instruments. For the instruments used, please state the country of manufacture and model number. Indicate if the data collection tools used were standardised.

RESULTS

In Table 2 description, please describe briefly the results of the different insulin resistance measures stratified by COVID-19 status instead of combining them as "the prevalence of insulin resistance ranged from 74.31 % to 89.61 % depending on the surrogate marker used".
In Table 2. Please add a statistical test for the generation of p-values as a footnote to the table.

Figure 2: Prevalence of abnormal levels of blood-based indices. Please provide a brief narrative of the prevalence of insulin resistance by the different measures in Figure 2.
You can provide the prevalence of insulin resistance stratified by COVID-19 status.
It is difficult to find the results for Figure 2 which is located at the end of the manuscript. Can you provide a narrative for Figure 2 after its subheading on page 9 if the journal guidelines indicate that the Figure should be at the end?

Each description of the findings in the tables (1-6) should be preceded by an appropriate subheading to ensure clarity.

DISCUSSION

Can you please discuss why you think participants from Tamale had significantly lower odds of insulin resistance compared to those in Cape Coast?

REFERENCES

Please ensure you include current references (10 years and below) since some of the references are very old.
